# Genomic islands of divergence and their consequences for the resolution of spatial structure in an exploited marine fish

**DOI:** 10.1111/eva.12026

**Published:** 2013-01-21

**Authors:** Ian R Bradbury, Sophie Hubert, Brent Higgins, Sharen Bowman, Tudor Borza, Ian G Paterson, Paul V R Snelgrove, Corey J Morris, Robert S Gregory, David Hardie, Jeffrey A Hutchings, Daniel E Ruzzante, Christopher T Taggart, Paul Bentzen

**Affiliations:** 1 Fisheries and Oceans CanadaSt. John's, NF, Canada; 2 Department of Biology, Marine Gene Probe Laboratory, Dalhousie UniversityHalifax, NS, Canada; 3 Ocean Sciences Center and Biology Department, Memorial University of NewfoundlandSt. John's, NF, Canada; 4 Atlantic Genome CenterHalifax, NS, Canada; 5 Department of Plant and Animal Sciences Faculty of Agriculture, Dalhousie UniversityTruro, NS, Canada; 6 Department of Oceanography, Dalhousie UniversityHalifax, NS, Canada

**Keywords:** Atlantic cod, divergent selection, genome scan, outlier loci, population genomics, single nucleotide polymorphism

## Abstract

As populations diverge, genomic regions associated with adaptation display elevated differentiation. These genomic islands of adaptive divergence can inform conservation efforts in exploited species, by refining the delineation of management units, and providing genomic tools for more precise and effective population monitoring and the successful assignment of individuals and products. We explored heterogeneity in genomic divergence and its impact on the resolution of spatial population structure in exploited populations of Atlantic cod, *Gadus morhua*, using genome wide expressed sequence derived single nucleotide polymorphisms in 466 individuals sampled across the range. Outlier tests identified elevated divergence at 5.2% of SNPs, consistent with directional selection in one-third of linkage groups. Genomic regions of elevated divergence ranged in size from a single position to several cM. Structuring at neutral loci was associated with geographic features, whereas outlier SNPs revealed genetic discontinuities in both the eastern and western Atlantic. This fine-scale geographic differentiation enhanced assignment to region of origin, and through the identification of adaptive diversity, fundamentally changes how these populations should be conserved. This work demonstrates the utility of genome scans for adaptive divergence in the delineation of stock structure, the traceability of individuals and products, and ultimately a role for population genomics in fisheries conservation.

## Introduction

The presence of adaptive diversity is a principle consideration in the management and conservation of exploited species (Fraser and Bernatchez 2001; Hilborn et al. 2003; Schindler et al. 2010). Despite its recognized importance, knowledge of adaptive diversity is presently lacking in many exploited species, often impeding management efforts. Recent studies examining genomewide variation among individuals and populations indicate variable levels of differentiation across the genome, referred to as ‘genomic islands of divergence’ (Wu 2001; Turner et al. 2005; Nosil et al. 2009). And although a suite of factors may influence the distribution and size of divergent regions including genetic conflict, genetic drift, mutation rates, and chromosomal structure, divergent selection and adaptation are often implicated (Nosil et al. 2009; Feder and Nosil 2010; Bierne et al. 2011). A link between divergent regions and adaption is supported by associations with previously identified QTL and annotated genes (e.g. Rogers and Bernatchez 2007; Via and West 2008), or environmental gradients (e.g. Nielsen et al. 2009b; Bradbury et al. 2010). Moreover, these genomic regions may be enriched for nonsynonymous substitutions (Hancock et al. 2011) or display parallel associations with independent habitats (Bradbury et al. 2010; Deagle et al. 2011). Genome scans for signatures of directional selection may thus provide direct insight into the presence of adaptive divergence often unobtainable with other methods and central to the successful management of wild populations.

The ability to resolve genomic regions associated with adaptation (e.g. Fournier-Level et al. 2011; Hancock et al. 2011) provides multiple opportunities for insight into the scales of ecological and evolutionary population dynamics, both directly applicable to management and conservation efforts (e.g. Schindler et al. 2010; Nielsen et al. 2012). First, as conservation units such as evolutionarily significant units (ESUs) are often defined as a population or group of populations possessing both demographic isolation and adaptive or ecological significance (Waples 1995; Fraser and Bernatchez 2001), examinations of genomic regions associated with adaptation can directly inform ESU designation (Funk et al. 2012). In conjunction with improved resolution of population structure, loci associated with signatures of adaptive divergence and increased population differentiation can enhance individual assignment success (e.g. Nielsen et al. 2012). For instance, gene-associated markers displaying signatures of directional selection provided unprecedented power to assign individuals and products to the population of origin in several commercially important European fish species (Nielsen et al. 2012). Finally, the identification of genomic regions associated with functional variation provides the opportunity to monitor the population responses to factors such as climate or harvest pressure (e.g. Schwartz et al. 2007). However, the tools to achieve these goals for many groups of commercially exploited marine fishes have thus far remained elusive, limited by apparent widespread genetic homogeneity (Hauser and Carvalho 2008).

Our study integrates SNP-based genome scans for elevated divergence in a marine fish, Atlantic cod (*Gadus morhua*), with linkage mapping information to demonstrate the utility of genome scans to resolve adaptive divergence and to inform management and conservation of exploited marine species. First, we use tests for selection and linkage information to explore the genomic distribution of outliers identified as potentially experiencing directional selection. Second, we examine the subsequent impact of adaptive variation on the resolution of spatial population structure and assignment success in Atlantic cod. We build on a previous study (Bradbury et al. 2010) that examined loci displaying evidence of environmentally associated selection in parallel with either side of the Atlantic in a subset of these loci (*n* = 40) and populations (*n* = 14). Here, we examine the distribution of divergence and its consequences for the resolution of spatial structure, providing critical insight into the geographic scale of population dynamics in exploited populations of Atlantic cod.

## Materials and methods

### Sample collection and location characteristics

**Figure 1:**
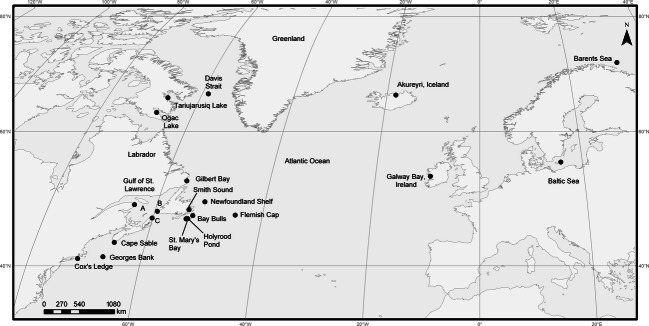
Map of sampling locations of Atlantic cod from across its geographic range. See Table S1 for further sample details.

We sampled individuals (*N* = 466) from 23 locations throughout the North Atlantic from 1996 to 2007 (See Fig. 1 for approximate locations, Table S1) during the course of scientific surveys or commercial harvest. With the exception of Arctic and northern locations where sampling was restricted to summer months, most fish were in spawning condition. Specific details regarding some samples and locations were published elsewhere (Taggart and Cook 1996; Hubert et al. 2009; Bradbury et al. 2010; Hubert et al. 2010) and a subset of these samples analyzed previously (Bradbury et al. 2010, 2011). We isolated DNA from these samples from ethanol-preserved fin clips using a modification of a previously published glass milk procedure (Elphinstone et al. 2003).

### SNP genotyping and linkage

Details on SNP development and genotyping were provided elsewhere (Bowman et al. 2010; Hubert et al. 2010). We chose 1536 SNPs (GenBank dbSNP under accession numbers ss131570222–ss131571915) for genotyping and assessed linkage using JoinMap4® (Van Ooijen 2006) and three families, including parents and F1 offspring (Borza et al. 2010). Linkage maps were generated using the group function of JoinMap4®, a LOD cutoff value of 5.0 or greater, and Haldane's mapping function. To allow the outlier loci to be mapped, we allowed JoinMap4® to force additional markers with a lower goodness of fit into the map, producing a map with 1295 SNPs (see Supporting Information for further details). Because previous work suggests that both *Pan*I and hemoglobin beta 1 in cod are under selection (Case et al. 2005; Andersen et al. 2009), we included SNPs associated with these genes for comparison on the linkage map (see Borza et al. 2010 for SNP details).

### Data analysis

Estimates of heterozygosity, population differentiation (*F*_ST_, both locus specific and global), and tests for Hardy–Weinberg equilibrium were performed using ARLEQUIN (Excoffier and Lischer 2010). For outlier detection, we used a Bayesian approach that directly estimates the posterior probability that a given locus is under selection by defining two alternative models, one with and one without the effect of selection. The respective posterior probabilities of each model are estimated using a reversible jump Markov chain Monte Carlo (MCMC) approach as implemented in the software BAYESCAN v2.01 (Foll and Gaggiotti 2008). Because geographic isolation or demographic structure may contribute to false positives (Foll and Gaggiotti 2008), we ran the analysis for the entire dataset (See Supporting Information) and then assessed population structure using principal coordinate analysis (PCoA) with initial significant outliers removed. The BAYESCAN analysis was then repeated on the large dominant cluster representing the western Atlantic using all loci. We compared the BAYESCAN results to an alternative method for outlier identification using a hierarchical island model (number of groups = 10) to generate the distribution of genetic variation within and among populations as implemented in ARLEQUIN (Excoffier and Lischer 2010).

Principle coordinate analysis was carried out using GenAlEx (vers.6.1; Peakall and Smouse 2006). Because each SNP likely represents a single gene and linkage disequilibrium may result from selection associated with adaptive divergence, we did not exclude loci that displayed correlations in allele frequency. We previously showed that linkage disequilibrium among outliers occurs even among chromosomes in this SNP panel (Bradbury et al. 2010). Distance among samples on the PCoA was calculated as the Euclidean distance among sample average values. We took geographic distance among samples as the shortest distance among sample locations along the continental shelves. We then used Bayesian clustering in BAPS (Corander and Marttinen 2006) to examine the number of groups (*K*) consistent with multilocus genetic data. This approach uses a stochastic optimization procedure rather than MCMC to identify the number of groups. We ran BAPS with the predefined number of clusters = 2–25 and replicated five times to ensure the stability of results. Assignment power of the SNP panel was evaluated using the proportion of successful identifications to the population of origin identified using GeneClass2.0 (Piry et al. 2004) and a subset of individuals (Anderson 2010). We used a Bayesian approach for assignment (Rannala and Mountain 1997) with resampling (Paetkau et al. 2004), and assignment success was evaluated for the western Atlantic using populations or groups identified in clustering analyses above and separately for all SNPs and only the neutral SNPs.

## Results

Although we attempted genotyping of the 23 population samples (466 individuals) for 1536 previously identified informative loci, the failure of some assays reduced successful loci to 1405. Observed (expected) heterozygosity overall was 0.307 (0.301) and ranged from 0.222 (0.220) in the Baltic Sea sample to 0.385 (0.360) for the Gulf of St. Lawrence and Georges Bank (See Table S1). The percentage of polymorphic loci in each sample varied from 69.0% in the Baltic to 99.3% in the Cape Sable sample (Table S1). Tests for departures from Hardy–Weinberg equilibrium indicated that approximately 1% of population/locus combinations (*n* = 33 720) were significant at α = 0.01. Locus-specific *F*_ST_ values were as high as 0.60 for some loci; the median and maximum pairwise population *F*_ST_ values were 0.13 and 0.50, respectively.

**Figure 2:**
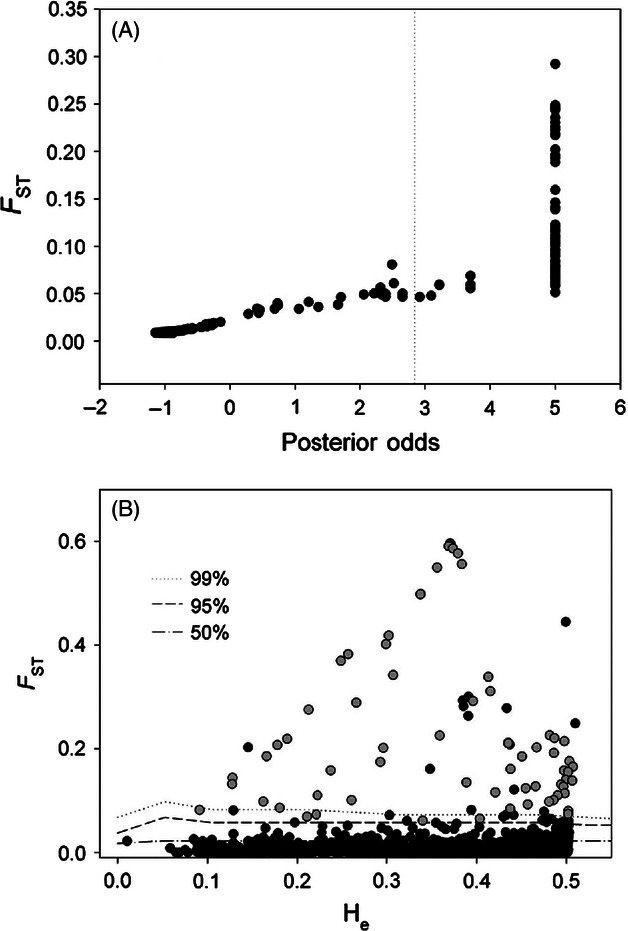
Tests for the presence of selection on SNPs genotyped in Atlantic cod from the western Atlantic. (A) Results of Bayesian tests for selection using BAYESCAN v2.01, and (B) results from a hierarchical island model-based test for selection, using ARLEQUIN v3.55. Gray dots represent outliers identified using BAYESCAN and outliers identified at a false discovery rate (FDR) of 1%. The dotted line in (A) represents the threshold for neutral loci, and in (B), the dashed and dotted lines represent the 50, 95, and 99 percentiles.

Tests for the signatures of selection identified multiple loci putatively experiencing directional selection. Using BAYESCAN and focusing on the western Atlantic, and excluding isolated populations (i.e. Arctic lakes and Gilbert Bay; see Materials and methods), we identified 73 loci potentially experiencing directional selection at a false discovery rate of 1% (Fig. 2A). In comparison, the hierarchical island model-based test implemented in ARLEQUIN (Excoffier et al. 2009) using all samples identified selection in 89 loci at α = 0.01 (Fig. 2B). Only six of the loci identified by BAYESCAN were not significant in ARLEQUIN. Given the similarity among the test results and lower types I and II errors in BAYESCAN (Narum and Hess 2012), we chose the loci identified using BAYESCAN for subsequent analysis.

**Figure 3:**
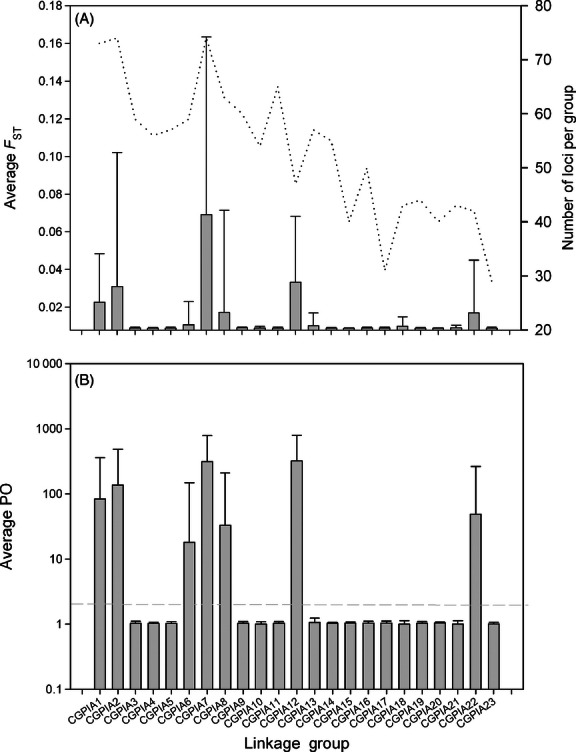
Average divergence (A) and posterior odds ratio (B) by linkage group (LG) (error bars represent standard deviation) for samples from the western Atlantic. Dashed line in (A) represents the number of loci per LG, and in (B) the threshold (posterior odds ratio) for significant tests for selection using BAYESCAN.

**Figure 4:**
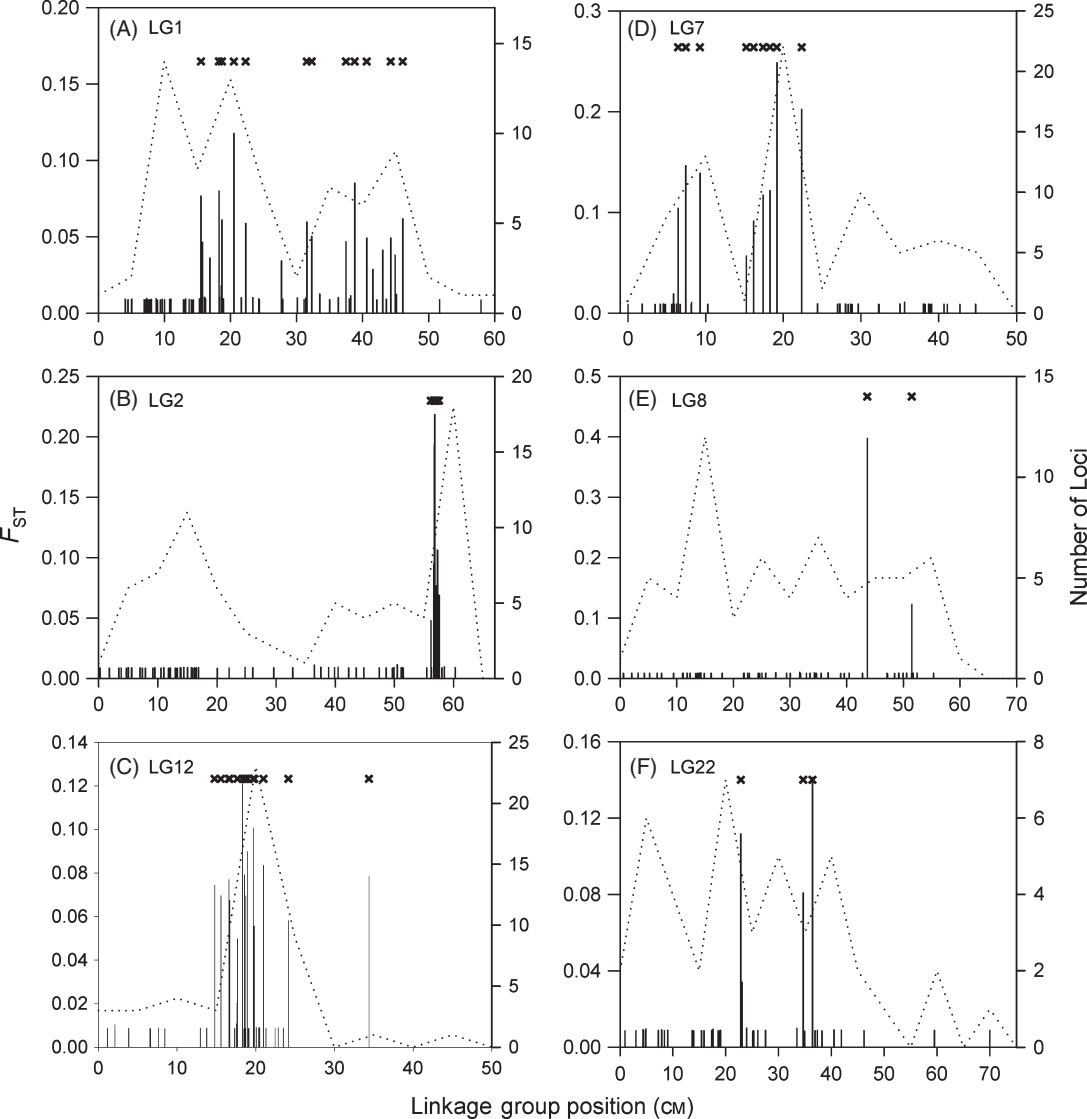
The distribution of genetic differentiation (*F*_ST_) across each of the linkage groups displaying significant tests for selection for western Atlantic samples. Dashed lines represent the number of loci per genomic region, and crossed line symbols the map positions which tested positive for selection using BAYESCAN. *Pan*I is located in LG1 at 31.5 cM and the hemoglobin β1 in LG2 at 36.5 and 38.8 cM. Note different *y*-axis scales in each plot.

The number of SNPs that mapped to particular linkage groups (LGs) among the 23 identified LGs ranged from fewer than 40 (LG17, LG23; Fig. 3A) to more than 70 (LG1, LG2, and LG7). Average *F*_ST_ per LG among nonisolated western populations varied by more than an order of magnitude (Fig. 3A) to as high as 0.06 (LG7), but the majority (65%) of values were low (<0.01). The proportion of outliers observed per LG differed significantly from random expectations (*G*-value: 149.40, df = 21, *P* < 0.001) with eight LGs containing loci identified as putatively experiencing selection (LG1, LG2, LG6, LG7, LG8, LG12, LG13, and LG22). Average posterior odds (BAYESCAN) per LG was above neutral expectations for the seven of the eight LGs that possessed loci identified as potentially experiencing selection (Fig. 3B); only LG13 was not elevated on average. Within each of the identified eight LGs, the numbers of outliers ranged from 1 (LG8 and LG13) to 25 (LG7), and multiple outlier SNPs were distributed along genomic regions ranging from 1.38 cM (LG2) to 30.57 cM (LG1). Interestingly, LG7 displayed an unusually high number of outlier loci, -19-, located at a single map position (19.12 cM; Fig. 4). In addition, peaks in the number of outlier loci at map locations for LGs 1, 2, 7, and 12 corresponded with peaks in the magnitude of differentiation observed, supporting a tendency for outliers to co-occur in genomic regions of high divergence.

**Figure 5:**
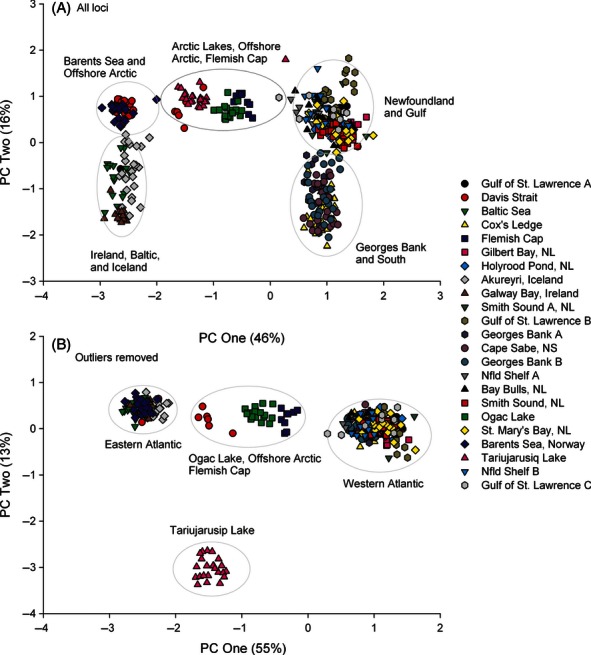
Principle coordinate analysis of SNPs from rangewide samples of Atlantic cod, using (A) all SNPs and (B) only neutral SNPs.

**Figure 6:**
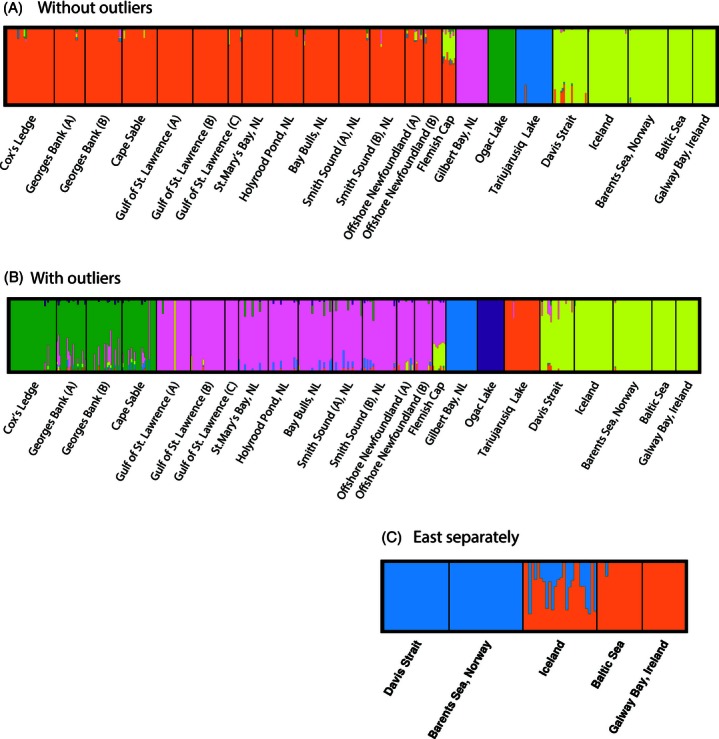
Bayesian clustering (BAPS) analysis of SNPs from rangewide samples of Atlantic cod using (A) only neutral SNPs, (B) all SNPs, and (C) all SNPs in the eastern Atlantic only.

Principle coordinate analysis (PCoA) using all SNPs produced a two-dimensional representation with the first two axes explaining 62% of the variance in the complete dataset (Fig. 5A). Five distinct groupings represent the north and south (PC2) components in the East/North and West Atlantic (PC1), as well as a cluster intermediate on PC1 containing the Arctic lakes, the Flemish Cap, and a portion of the offshore Arctic (Fig. 5A). Removal of outlier SNPs prior to PCoA resulted in the loss of fine-scale geographic separation (principally latitudinal structure) on PC2 (Fig. 5B). Dominant groupings represented the East (including some of the offshore Arctic Davis Strait samples), the West Atlantic south of Labrador, and isolated Arctic and northern locations (Fig. 5B). Euclidean distance calculated from the PCoA associated strongly with geographic distance for the complete dataset (*R*^2^ = 0.75, *P* < 0.001), but the removal of outlier SNPs from the analysis emphasized the influence of the three distinct groups (Figure S1A,B). Bayesian clusters in BAPS were consistent with the PCoA groups. Clustering after removing outliers revealed five clear groups consistent with the isolation of the Arctic lakes, Gilbert Bay, and East and West Atlantic (Fig. 6A). Clustering using the complete dataset revealed six discrete clusters, including five from the western Atlantic (Fig. 6B); however, the single eastern Atlantic group resolved into two groups when we analyzed this group in isolation (Fig. 6C). These clusters represent northern and southern components in the East and West Atlantic and the isolated Arctic Lakes and Gilbert Bay. Again a portion of the Davis Strait/offshore Arctic sample clustered with eastern samples. In both analyses, the Flemish Cap samples associated with other Newfoundland samples, although they also displayed a clear affinity to eastern Atlantic cod populations, which was evident in all individuals (Fig. 6). Assignment success was high with 100% of individuals assigned correctly to population or cluster of origin using either the nonoutlier or the complete SNP panel. The ratio of the likelihood of correct versus incorrect assignment was similar for isolated locations for the nonoutlier loci alone versus the complete panel, but was significantly higher (anova, *P*-value < 0.0001) for the panel with outlier loci included in the nonisolated populations.

## Discussion

The successful management of capture fisheries necessitates the representation of adaptive diversity in conservation units (Hilborn et al. 2003; Schindler et al. 2010), the ability to monitor populations for change (Schwartz et al. 2007), and the power to track individuals and products to identify illegal harvest (e.g. Nielsen et al. 2012). The tools to achieve these goals for many groups of commercially exploited marine fishes have thus far remained elusive, limited by apparent widespread genetic homogeneity. However, recent advances in characterizing genomewide adaptive population structuring can resolve unprecedented levels of diversity in marine species (e.g. Limborg et al. 2012) and will likely change how capture fisheries are managed (e.g. Funk et al. 2012). Our observations of several genomic regions of elevated divergence clustered across multiple LGs suggest nonrandom distribution of adaptive variation across the cod genome. Obvious geographic barriers define spatial structuring at neutral loci. The inclusion of outlier SNPs revealed additional barriers to gene flow within both the eastern and western Atlantic. The resolution of genomic signatures of adaptive diversity creates the opportunity for population genomic monitoring, because identified outliers link with ocean climate (Bradbury et al. 2010), and the tracking and assignment of individuals and products to region of origin in this species (e.g. Nielsen et al. 2012). Our results highlight the importance of resolving genomic regions of adaptive divergence for the management and conservation of exploited marine species.

Genome scans can potentially resolve adaptive genetic variation and inform management or conservation strategies in species, which are difficult if not impossible to study through other means (Funk et al. 2012). From a management perspective, the representation of adaptive variation among management units is critical to protecting adaptive diversity essential to a species persistence and stability (Hilborn et al. 2003; Schindler et al. 2010). Our observations of an adaptive cline in Atlantic cod in the region of the Scotian Shelf, which separated populations to the north and south, resulted in a revision of the number of conservation units in this species in Canadian waters (COSEWIC 2010). Similar observations of fine-scale adaptive diversity in other marine fish species such as Atlantic herring (e.g. Limborg et al. 2012) are beginning to accumulate. These observations support the hypothesis that local adaptive diversity in marine fishes may be common (Hauser and Carvalho 2008) and may therefore represent critical considerations in successful management strategies.

The utility of genomic resources for the monitoring of marine populations requires a clear understanding of the function of SNPs or identified genes. Although the functional relationships among these outlier genes remain undetermined, observations are consistent with a previous study identifying temperature associations in a subset of these outliers and populations. Bradbury et al. (2010) identified strong temperature associations on either side of the Atlantic in over half of these outliers (55%), including those clustered in LG7. Our results here, based on more extensive sampling, support spatial trends reported previously and the hypothesis that a large portion of the outliers we identify here link with temperature; however, the genes involved remain to be identified. Associated work identified a QTL for body weight located at map position 19.12 on LG7 (S. Bowman, personal communication). Again this map location is characterized by a large region of low recombination and contains 26% of the identified outliers. Associations among some of these outliers with body size or ocean temperature (Bradbury et al. 2010) suggest an opportunity for their use in the monitoring of population responses to size-selective harvest (e.g. Jakobsdóttir et al. 2011) or climate change; though, these applications require further evaluation. The recent completion of the Atlantic cod genome (Star et al. 2011) should help resolve several of these outstanding issues.

The observed signals of fine-scale geographic differentiation at these gene-associated, high-divergence SNPs also provide the opportunity for accurate assignment of individuals and products to populations or regions of origin. Our study indicates very high success of assignment to cluster, but also that inclusion of outlier loci significantly improved support for assignments. Nielsen et al. (2012) evaluated a similar panel of SNPs for assignment success in Atlantic cod in the eastern Atlantic and concluded that eight of the most divergent loci were required for accurate assignment. In future, similar minimal panels of maximum assignment power could be developed from current studies and tailored to specific regional management issues. Such applications would not have been possible using microsatellite (e.g. Bentzen et al. 1996)- or mtDNA (Carr and Marshall 2008)-based approaches in the western Atlantic because of low levels of spatial differentiation, highlighting the utility of genomewide SNP approaches. Admittedly, the geographic scale of structuring observed at our outlier SNPs in the western Atlantic is often larger than several current stock management units (COSEWIC 2010), and further demographic structuring likely exists within our clusters.

Overall, we identified 5.2% of our surveyed SNPs as outliers, a pattern consistent with past studies that indicated 5–10% of a genome may display signatures of selection (Nosil et al. 2009; Strasburg et al. 2012); though, individual studies reported values ranging from 0.5% (Miller et al. 2007) to approximately 25% (Vasemägi et al. 2005). Part of this variation can likely be explained by the marker type used, given that expressed sequence-based loci or candidate genes can display a higher propensity to test positive for selection (e.g. Vasemägi et al. 2005; Nielsen et al. 2009a). As such, our use of EST-associated SNPs might partly explain the relatively high number of outliers we detected. Despite our success in identifying regions associated with divergence, we likely missed genomic regions experiencing weak selection. The frequency and nature of outlier SNPs observed here might well be influenced by the fact that we obtained these SNPs from a region in the western Atlantic that fortuitously has both previously identified cod temperature haplotypes (Bradbury et al. 2010), but is otherwise limited in its representation of cod habitat and diversity. Had we developed the SNPs using a rangewide ascertainment panel, and we might well have discovered many SNPs associated with other types of selective forces. In addition, direct experimental tests of selection and finer-scale sequencing commonly identify widespread genomic divergence resulting from selection and speciation, often contrasting earlier works that identify only a few such regions (e.g. Michel et al. 2010; Hahn et al. 2012). Further examination will likely build on the regions of divergence identified here.

The roles ascertainment bias or temporal variation among samples may play in the observed spatial patterns, or potential assignment success remains unclear. Ascertainment bias is evident in the present dataset as a decline in diversity from west to east associated with distance from the location of the ascertainment panel (Bradbury et al. 2011). We evaluated the impact of ascertainment bias on the accuracy of individual assignment, and although significant declines in assignment power were visible with some subsets of SNPs, overall there are no reductions in power for the complete panel (Bradbury et al. 2011). Sampling in the eastern Atlantic was limited, however, and in conjunction with lower observed diversity of the SNPs in the east, suggests that we may have missed significant population structure. In addition, because the samples used in our study span a 14-year period, temporal variation in allele frequency may contribute to the patterns observed. However, recent work based on a subset of these SNPs observed temporal stability over 10-year period (Nielsen et al. 2012) in European samples. Although the contribution of temporal variation to the observed spatial trends cannot be discounted, given the length of the period (2–3 generations), and evidence of stability elsewhere, we infer minimal bias in relation to the spatial trends observed.

Clustering of divergent loci within genomic regions may be common (McGaugh and Noor 2012; Via 2012) and particularly prevalent in the regions of low recombination (Nosil and Feder 2012). Across the Atlantic cod genome, the distribution of genetic divergence was not random but clustered in several LGs and map locations within LGs. This clustering was most apparent in LG7 where multiple outlier SNPs clustered to a single map location. Undoubtedly, some of this clustering could be associated with genetic hitchhiking (Maynard-Smith and Haigh 1974). Similar outlier clustering was reported in the house mouse (*Mus musculus*) where divergent regions accounted for 7–8% of the genome and associated with eight genomic regions (Harr 2006). Among forms of the mosquito, *Anopheles gambiae,* divergence was associated with 1.2% of the genome and distributed among three genomic regions (Turner et al. 2005). Our observation of clustering among outliers is consistent with the hypothesis that mutations contributing to divergence should accumulate in the regions of genome already experiencing divergent selection (Navarro and Barton 2003; Kirkpatrick and Barton 2006; Nosil et al. 2009) or experiencing low recombination rates (McGaugh and Noor 2012; Nachman and Payseur 2012). Significant linkage disequilibrium among outliers may inflate spatial trends if compared SNPs are not independent. Although we cannot fully discount inflated trends here, we believe it unlikely given that several outliers mapped to different LGs or locations within a LG. Nonetheless, until a physical map is available or the SNPs successfully annotated we cannot rule out potential for bias.

Our spatial analysis revealed several patterns that extend beyond that of increased spatial isolation with outlier loci. First, several locations in the Arctic and the Flemish Cap sample appear genetically intermediate between the eastern and western Atlantic on the first axis of the principle coordinate analysis. Moreover, some of the offshore Arctic Davis Strait samples cluster with the eastern Atlantic. Both observations contradict a hypothesis of long standing vicariance and support a hypothesis of eastern Atlantic colonization in the Arctic or historic trans-Atlantic gene flow. However, formally testing this hypothesis remains challenging in light of the potential confounding influence of ascertainment bias. Nonetheless, individuals from the Flemish Cap display eastern Atlantic affinities in the BAPS analysis mirroring observations based on whole mtDNA genome analysis of Flemish Cap cod which share ancestral clades with Barents Sea cod (S.M. Carr and H.D. Marshall, personal communication). Previous studies report similar trans-Atlantic trends in genetic structure and the presence of eastern Atlantic genotypes in the northwest Atlantic for both marine and anadromous fishes. In Atlantic wolffish (*Anarhichas lupus*), which is widely distributed across the Atlantic, a dominant genetic break separates populations to the southwest and east of western Greenland with some eastern affiliations of populations along the northern Grand Banks off Newfoundland (McCusker and Bentzen 2010). Similarly in Atlantic salmon (*Salmo salar*), longstanding genetic isolation occurs in the south on either sides of the Atlantic, but again Newfoundland and Labrador consistently contain populations characterized by eastern Atlantic genotypes (King et al. 2007). Together, these observations support a hypothesis of past trans-Atlantic gene flow perhaps associated with warm interglacial periods allowing the colonization of Canadian Arctic marine and lake habitats from the eastern Atlantic. In conjunction with the observations of fine-scale adaptive divergence, our observations of potential trans-Atlantic gene flow provide novel insight into the past and future spatial dynamics of cod in the North Atlantic.

## Summary

Genomic regions associated with adaptation may directly inform conservation efforts in exploited marine species, by delineating management units, and providing genomic tools for population monitoring and individual assignment. We observed significant variation in the genomic and geographic scale of differentiation in Atlantic cod, using a panel of EST-associated SNPs. Genomic islands of divergence were associated with several LGs and in several instances clustered significantly, supporting the hypothesis that divergent regions co-occur in response to strong selection or low recombination. Outlier SNPs, likely associated with adaptive divergence, resolved fine-scale structure not evident at neutral loci and provide ample power for successful individual assignment to the region of origin. Geographic population differentiation in Atlantic cod appears to be distinguished by a few discrete islands of genomic divergence, although finer-scale sequence analysis and selection experiments are needed to further resolve the genomic scale of divergence. This work demonstrates the utility of genomic regions of adaptive divergence in the delineation of stock structure, traceability of individuals and products, and ultimately a role for population genomics in the conservation of marine fisheries.

## Data archiving statement

Details on SNP development and genotyping are available in the studies by Bowman et al. (2010) and Hubert et al. (2010). The SNPs analyzed in this study are available in GenBank dbSNP (accession numbers: ss131570222–ss131571915). Additional methods and results can be found as Supporting Information.

## Supporting Information

**Figure S1.** Euclidean distance among population average PCoA values of SNPs from range-wide samples of Atlantic cod using (A) all SNPs and (B) only neutral SNPs. **Table S1.** Details on sample locations and SNP summary statistics. **Table S2.** Pairwise *F*_ST_ values for all samples and all SNPs. **Table S3.** Pairwise *F*_ST_ values for all samples and all non-outlier SNPs. **Table S4.** Results of BAYESCAN analysis with all samples included. **Data S1.** Methods.

Data S2 LGMAP1.

Data S3 LGMAP2.

Data S4 Linkage maps 1 and 2.
