# Supplementary material for: Genomic islands of divergence and their consequences for the resolution of spatial structure in an exploited marine fish
Source: Evol Appl. 2013 Jan 21;6(3):450–61. doi: 10.1111/eva.12026 (PMC3673473; doi:10.1111/eva.12026)
Supplement: Supplementary file 1 — Figure S1. Euclidean distance among population average PCoA values of SNPs from range-wide samples of Atlantic cod using (A) all SNPs and (B) only neutral SNPs. Table S1. Details on sample locations and SNP summary statistics. Table S2. Pairwise FST values for all samples and all SNPs. Table S3. Pairwise FST values for all samples and all non-outlier SNPs. Table S4. Results of BAYESCAN analysis with all samples included. Data S1. Methods. [file eva0006-0450-sd1.pdf]

Genomic islands of divergence and their consequences for the resolution of spatial structure in an exploited marine fish

Ian R. Bradbury[1,4,5*], Sophie Hubert[2], Brent Higgins[2], Sharen Bowman[2], Tudor Borza[2,3], Ian G. Paterson[4], Paul V.R. Snelgrove[5], Corey J. Morris[1], Robert S. Gregory[1], David Hardie[4], Jeffrey A. Hutchings[4], Daniel E.Ruzzante[4], Christopher T. Taggart[6], Paul Bentzen[4]

[1]Department of Fisheries and Oceans, 80 East White Hills Road, St. John's, Newfoundland, Canada, A1C 5X1. [2]Atlantic Genome Center, Halifax, Nova Scotia, Canada, B3H3Z1, [3]Department of Plant and Animal Sciences, Faculty of Agriculture, Dalhousie University, Truro, NS, B2N 5E3, Canada, [4]Marine Gene Probe Laboratory, Department of Biology, Dalhousie University, 1359 Oxford Street, Halifax Nova Scotia, B3H4R2., [5]Ocean Sciences Center and Biology Department, Memorial University of Newfoundland, St. John's, NL A1C 5S7, P.O. Box 4200, CANADA. [6]Department of Oceanography, Dalhousie University, 1359 Oxford Street, Halifax Nova Scotia, B3H4R2 *To whom correspondence should be addressed. Email: ibradbur@me.ca

THE PDF INCLUDES:
METHODS
TABLES S1-S4
FIGURE S1

Methods

Sample Collection and Location Characteristics

Individuals were sampled at 23 locations from throughout the North Atlantic (Fig 1, Table S1) from 1996 to 2007. Sample sizes ranged from 15 to 26. Samples were collected as part of scientific surveys or commercial harvest and primarily targeted fish in spawning condition with the exception of Ogac Lake and Gilbert Bay where sampling was restricted to summer months. Specific details regarding some samples and locations are published elsewhere (e.g., Taggart and Cook 1996; Bradbury et al. 2009; Hubert et al. 2009, 2010). Although the potential for mixing of local stocks within our samples exists, the large geographic scale examined here and the nature of the trends observed should minimize the impact of small scale mixtures, but this cannot be discounted.

SNP Development and Linkage Mapping

The initial cDNA libraries were developed using 884 individuals (Bowman et al. 2010) focusing on SNPs with > 100 bp of flanking sequence. Of the 3072 putative SNPs identified, 2284 (~74%) were selected following screening, of which 1641 were informative (53%). Elsewhere we have argued that these SNPs were identified from sequencing ESTs averaging 800-1000bp in length from the 3' end and likely represent single genes (Bradbury et al. 2010). The only exception was a pair of SNPs (S1039a and S1039b) identified from a contig. The genetic linkage map was constructed using JoinMap4® (Van Ooijen 2006) and the three families described elsewhere (Bradbury et al 2010 and Borza et al 2010). Mapping was performed using a LOD cut-off value of 5.0 and Haldane's mapping function (for more details on mapping see Hubert et al 2010, Bradbury et al 2010 and Borza et al 2010). The map generated by the first round of calculations of the regression mapping algorithm included only 987 SNPs and contained 58% of

outliers identified using Bayescan (LGMAP1). A significantly higher number of outlier loci were mapped in the third round of mapping calculations where JoinMap®4 was allowed to force additional markers with a lower goodness-of-fit into the map (for further details and description of a similar map see Borza et al. 2010). This approach resulted in 1295 SNPs being mapped successfully (LGMAP2) and contained all outliers. Comparison of the two maps revealed little change in map position of the outlier SNPs which were in common (median change in position of 0.69 cM). Accordingly, LGMAP2, generated by the third round of mapping calculations, was used in all subsequent analyses.

Table S1. Details on sample locations and SNP summary statistics. See Methods for further information.

Location	Year	Previously analyzed	N	Ho	He	% Polymorphic
1. Cox's Ledge	2008	No	31	0.358	0.358	99.00%
2. Georges Bank (A)	2006	Bradbury et al. 2010	24	0.361	0.360	99.22%
3. Georges Bank (B)	2008	Bradbury et al. 2010	20	0.363	0.357	98.93%
4. Cape Sable, NS	2006	Bradbury et al. 2010	23	0.362	0.359	99.29%
5. Gulf of St. Lawrence (A)	1996	No	23	0.346	0.342	97.30%
6. Gulf of St. Lawrence (B)	1996	No	9	0.363	0.338	92.60%
7. Gulf of St. Lawrence (C)	2002	No	23	0.385	0.345	96.73%
8. Flemish Cap		No	9	0.313	0.305	87.40%
9. St. Mary's Bay, NL	2006	Bradbury et al. 2010	25	0.358	0.350	97.30%
10. Holyrood Pond, NL	2007	Bradbury et al. 2010	20	0.357	0.351	96.80%
11. Bay Bulls, NL	2007	Bradbury et al. 2010	23	0.350	0.353	97.22%
12. Smith Sound, NL (A)	2007	Bradbury et al. 2010	20	0.358	0.351	96.37%
13. Smith Sound B, NL (B)	2008	Bradbury et al. 2010	23	0.354	0.354	97.79%
14. Newfoundland Shelf (A)	2009	No	12	0.351	0.343	94.73%
15. Newfoundland Shelf (B)	2009	No	12	0.352	0.335	94.31%
16. Gilbert Bay, NL	2004	Bradbury et al. 2010	21	0.309	0.304	90.11%
17. Ogac Lake, Baffin Island	2004	Bradbury et al. 2010	18	0.253	0.253	76.23%
18. Tariujarusiq Lake, Baffin Island	2004	No	24	0.240	0.234	76.80%
19. Davis Strait	2009	No	23	0.264	0.258	87.40%
20. Barents Sea, Norway	2008	Bradbury et al. 2010	26	0.239	0.239	78.22%
21. Akureyri, Iceland	2008	Bradbury et al. 2010	26	0.258	0.258	85.05%
22. Baltic Sea	1996	Bradbury et al. 2010	16	0.222	0.220	69.40%
23. Galway Bay, Ireland	2008	Bradbury et al. 2010	15	0.242	0.236	74.73%

Table S2. Pairwise F_{ST} values for all samples and all SNPs. P-values above the diagonal and F_{ST} values below.

	1	2	3	4	5	6	7	8	9	10	11	12	13	14	15	16	17	18	19	20	21	22	23
1. Cox's Ledge		0.000	0.000	0.000	0.000	0.000	0.000	0.000	0.000	0.000	0.000	0.000	0.000	0.000	0.000	0.000	0.000	0.000	0.000	0.000	0.000	0.000	0.000
2. Georges Bank (A)	0.009		0.488	0.664	0.000	0.000	0.000	0.000	0.000	0.000	0.000	0.000	0.000	0.000	0.000	0.000	0.000	0.000	0.000	0.000	0.000	0.000	0.000
3. Georges Bank (B)	0.010	0.000		0.187	0.000	0.000	0.000	0.000	0.000	0.000	0.000	0.000	0.000	0.000	0.000	0.000	0.000	0.000	0.000	0.000	0.000	0.000	0.000
4. Cape Sable (NS)	0.007	0.000	0.001		0.000	0.000	0.000	0.000	0.000	0.000	0.000	0.000	0.000	0.000	0.000	0.000	0.000	0.000	0.000	0.000	0.000	0.000	0.000
5. Gulf of St. Lawrence (A)	0.052	0.034	0.040	0.042		0.190	0.305	0.001	0.002	0.013	0.000	0.003	0.000	0.117	0.156	0.000	0.000	0.000	0.000	0.000	0.000	0.000	0.000
6. Gulf of St. Lawrence (B)	0.054	0.037	0.042	0.045	0.002		0.275	0.000	0.000	0.000	0.000	0.000	0.000	0.000	0.013	0.000	0.000	0.000	0.000	0.000	0.000	0.000	0.000
7. Gulf of St. Lawrence (C)	0.048	0.033	0.037	0.040	0.002	0.001		0.000	0.000	0.000	0.000	0.000	0.000	0.013	0.008	0.000	0.000	0.000	0.000	0.000	0.000	0.000	0.000
8. St. Mary's Bay (NL)	0.044	0.021	0.025	0.028	0.010	0.010	0.009		0.287	0.862	0.084	0.221	0.000	0.000	0.000	0.000	0.000	0.000	0.000	0.000	0.000	0.000	0.000
9. Holyrood Pond (NL)	0.045	0.023	0.026	0.031	0.011	0.015	0.011	0.001		0.438	0.517	0.363	0.000	0.000	0.000	0.000	0.000	0.000	0.000	0.000	0.000	0.000	0.000
10. Bay Bulls (NL)	0.047	0.022	0.027	0.031	0.006	0.008	0.008	0.000	0.000		0.106	0.176	0.000	0.003	0.003	0.000	0.000	0.000	0.000	0.000	0.000	0.000	0.000
11. Smith Sound (NL)	0.044	0.021	0.024	0.028	0.015	0.018	0.015	0.002	0.000	0.001		0.040	0.000	0.000	0.000	0.000	0.000	0.000	0.000	0.000	0.000	0.000	0.000
12. Smith Sound (NL)	0.047	0.024	0.027	0.032	0.006	0.012	0.009	0.001	0.001	0.001	0.002		0.000	0.000	0.000	0.000	0.000	0.000	0.000	0.000	0.000	0.000	0.000
13. Gilbert Bay (NL)	0.100	0.080	0.085	0.086	0.080	0.078	0.074	0.064	0.059	0.061	0.058	0.065		0.000	0.000	0.000	0.000	0.000	0.000	0.000	0.000	0.000	0.000
14. Newfoundland Shelf (A)	0.047	0.034	0.038	0.042	0.003	0.010	0.007	0.010	0.010	0.006	0.013	0.008	0.074		0.290	0.000	0.000	0.000	0.000	0.000	0.000	0.000	0.000
15. Newfoundland Shelf (B)	0.052	0.036	0.040	0.045	0.002	0.004	0.008	0.013	0.016	0.007	0.019	0.007	0.084	0.001		0.000	0.000	0.000	0.000	0.000	0.000	0.000	0.000
16. Flemish Cap	0.086	0.069	0.076	0.078	0.036	0.041	0.040	0.053	0.054	0.040	0.058	0.046	0.122	0.035	0.029		0.000	0.000	0.000	0.000	0.000	0.000	0.000
17. Ogac Lake	0.161	0.142	0.147	0.155	0.145	0.146	0.137	0.132	0.131	0.130	0.135	0.133	0.193	0.140	0.147	0.149		0.000	0.000	0.000	0.000	0.000	0.000
18. Tariujarusiq Lake	0.159	0.145	0.151	0.157	0.136	0.132	0.126	0.141	0.145	0.131	0.146	0.135	0.211	0.124	0.124	0.063	0.168		0.000	0.000	0.000	0.000	0.000
19. Davis Strait	0.189	0.178	0.180	0.188	0.200	0.187	0.179	0.178	0.175	0.173	0.173	0.177	0.233	0.177	0.187	0.178	0.239	0.174		0.000	0.000	0.000	0.000
20. Akureyri, Iceland	0.141	0.126	0.132	0.135	0.168	0.164	0.152	0.153	0.153	0.146	0.152	0.151	0.214	0.152	0.158	0.109	0.170	0.042	0.184		0.000	0.000	0.000
21. Barents Sea, Norway	0.189	0.170	0.175	0.182	0.173	0.165	0.158	0.168	0.172	0.156	0.173	0.162	0.238	0.163	0.160	0.095	0.184	0.013	0.199	0.041		0.000	0.000
22. Baltic Sea	0.172	0.171	0.175	0.177	0.211	0.200	0.185	0.196	0.196	0.189	0.194	0.195	0.261	0.195	0.200	0.166	0.227	0.097	0.231	0.067	0.103		0.000
23. Galway Bay, Ireland	0.146	0.141	0.145	0.145	0.211	0.202	0.189	0.190	0.191	0.183	0.188	0.189	0.252	0.192	0.200	0.162	0.218	0.097	0.237	0.029	0.097	0.088	

Table S3. Pairwise F_{ST} values for all samples and all non-outlier SNPs. P-values above the diagonal and F_{ST} values below.

	1	2	3	4	5	6	7	8	9	10	11	12	13	14	15	16	17	18	19	20	21	22	23
1. Cox's Ledge		0.016	0.001	0.015	0.003	0.000	0.000	0.000	0.000	0.000	0.000	0.000	0.000	0.001	0.000	0.000	0.000	0.000	0.000	0.000	0.000	0.000	0.000
2. Georges Bank (A)	0.004		0.602	0.849	0.000	0.000	0.000	0.000	0.000	0.000	0.000	0.000	0.000	0.000	0.000	0.000	0.000	0.000	0.000	0.000	0.000	0.000	0.000
3. Georges Bank (B)	0.005	0.000		0.374	0.000	0.000	0.000	0.000	0.000	0.000	0.000	0.000	0.000	0.000	0.000	0.000	0.000	0.000	0.000	0.000	0.000	0.000	0.000
4. Cape Sable (NS)	0.004	0.000	0.000		0.000	0.000	0.000	0.000	0.000	0.000	0.000	0.000	0.000	0.000	0.000	0.000	0.000	0.000	0.000	0.000	0.000	0.000	0.000
5. Gulf of St. Lawrence (A)	0.015	0.010	0.012	0.012		0.255	0.282	0.024	0.114	0.097	0.005	0.076	0.000	0.147	0.301	0.000	0.000	0.000	0.000	0.000	0.000	0.000	0.000
6. Gulf of St. Lawrence (B)	0.016	0.012	0.014	0.014	0.001		0.437	0.000	0.000	0.000	0.000	0.000	0.000	0.000	0.100	0.000	0.000	0.000	0.000	0.000	0.000	0.000	0.000
7. Gulf of St. Lawrence (C)	0.015	0.012	0.013	0.014	0.002	0.000		0.001	0.000	0.000	0.000	0.000	0.000	0.016	0.026	0.000	0.000	0.000	0.000	0.000	0.000	0.000	0.000
8. St. Mary's Bay (NL)	0.012	0.005	0.007	0.007	0.004	0.005	0.006		0.366	0.921	0.162	0.285	0.000	0.000	0.000	0.000	0.000	0.000	0.000	0.000	0.000	0.000	0.000
9. Holyrood Pond (NL)	0.014	0.007	0.007	0.009	0.004	0.007	0.008	0.001		0.599	0.631	0.454	0.000	0.012	0.001	0.000	0.000	0.000	0.000	0.000	0.000	0.000	0.000
10. Bay Bulls (NL)	0.014	0.005	0.008	0.007	0.003	0.005	0.005	0.000	0.000		0.524	0.176	0.000	0.006	0.032	0.000	0.000	0.000	0.000	0.000	0.000	0.000	0.000
11. Smith Sound (NL)	0.014	0.006	0.007	0.007	0.005	0.009	0.010	0.001	0.000	0.000		0.376	0.000	0.000	0.000	0.000	0.000	0.000	0.000	0.000	0.000	0.000	0.000
12. Smith Sound (NL)	0.013	0.007	0.006	0.008	0.003	0.008	0.007	0.001	0.000	0.001	0.000		0.000	0.000	0.055	0.000	0.000	0.000	0.000	0.000	0.000	0.000	0.000
13. Gilbert Bay (NL)	0.069	0.064	0.068	0.064	0.068	0.066	0.067	0.063	0.059	0.057	0.058	0.061		0.000	0.000	0.000	0.000	0.000	0.000	0.000	0.000	0.000	0.000
14. Newfoundland Shelf (A)	0.012	0.012	0.013	0.014	0.002	0.010	0.007	0.007	0.006	0.005	0.007	0.006	0.065		0.711	0.000	0.000	0.000	0.000	0.000	0.000	0.000	0.000
15. Newfoundland Shelf (B)	0.015	0.012	0.014	0.015	0.001	0.002	0.006	0.007	0.008	0.003	0.010	0.003	0.072	0.000		0.000	0.000	0.000	0.000	0.000	0.000	0.000	0.000
16. Flemish Cap	0.039	0.034	0.037	0.037	0.027	0.031	0.027	0.036	0.033	0.027	0.035	0.031	0.096	0.024	0.021		0.000	0.000	0.000	0.000	0.000	0.000	0.000
17. Ogac Lake	0.128	0.122	0.124	0.129	0.129	0.128	0.123	0.121	0.121	0.122	0.125	0.122	0.182	0.127	0.132	0.134		0.000	0.000	0.000	0.000	0.000	0.000
18. Tariujarusiq Lake	0.103	0.101	0.104	0.107	0.109	0.107	0.099	0.111	0.110	0.104	0.112	0.105	0.172	0.095	0.100	0.052	0.150		0.000	0.000	0.000	0.000	0.000
19. Davis Strait	0.161	0.161	0.160	0.163	0.186	0.173	0.169	0.170	0.167	0.165	0.165	0.168	0.224	0.166	0.174	0.165	0.239	0.160		0.000	0.000	0.000	0.000
20. Akureyri, Iceland	0.108	0.103	0.105	0.108	0.127	0.124	0.115	0.119	0.118	0.114	0.118	0.115	0.178	0.112	0.120	0.075	0.155	0.015	0.166		0.000	0.000	0.000
21. Barents Sea, Norway	0.122	0.118	0.119	0.124	0.132	0.127	0.118	0.130	0.128	0.122	0.129	0.122	0.191	0.120	0.123	0.073	0.159	0.007	0.176	0.014		0.000	0.000
22. Baltic Sea	0.143	0.148	0.148	0.152	0.172	0.162	0.150	0.161	0.160	0.157	0.158	0.158	0.222	0.155	0.163	0.131	0.208	0.071	0.209	0.063	0.071		0.000
23. Galway Bay, Ireland	0.113	0.110	0.111	0.116	0.140	0.135	0.126	0.131	0.130	0.126	0.130	0.128	0.189	0.123	0.134	0.092	0.172	0.030	0.187	0.014	0.028	0.069	

Table S4. Results of BAYESCAN analysis with all samples included. Outliers identified with a FDR of 1%.

**Locus**	**log10(PO)**	F_{ST}
**cgpGmo_S1553a**	1000	0.57488
**cgpGmo_S1830**	1000	0.55475
**cgpGmo_S814a**	1000	0.5531
**cgpGmo_S152**	1000	0.55272
**cgpGmo_S1089**	1000	0.54629
**cgpGmo_S1039a**	1000	0.54412
**cgpGmo_S1183**	1000	0.54405
**cgpGmo_S2158**	1000	0.54276
**cgpGmo_S268**	1000	0.53285
**cgpGmo_S1039b**	1000	0.53192
**cgpGmo_S419**	1000	0.52775
**cgpGmo_S920**	1000	0.52774
**cgpGmo_S1810**	1000	0.52415
**cgpGmo_S1101a**	1000	0.51978
**cgpGmo_S1068**	1000	0.51305
**cgpGmo_S1986**	1000	0.49707
**cgpGmo_S674**	1000	0.48988
**cgpGmo_S1644**	1000	0.48592
**cgpGmo_S1497**	1000	0.46449
**cgpGmo_S1166**	1000	0.46404
**cgpGmo_S1874**	1000	0.46253
**cgpGmo_S1955**	1000	0.45884
**cgpGmo_S1205**	1000	0.44506
**cgpGmo_S2095**	1000	0.42932
**cgpGmo_S180b**	1000	0.41075
**cgpGmo_S1200**	1000	0.40829
**cgpGmo_S1867**	1000	0.40139
**cgpGmo_S1643**	1000	0.3992
**cgpGmo_S822a**	1000	0.39912
**cgpGmo_S1737**	1000	0.39826
**cgpGmo_S917**	1000	0.39212
**cgpGmo_S2019**	1000	0.38091
**cgpGmo_S1026**	1000	0.37877
**cgpGmo_S816a**	1000	0.3702
**cgpGmo_S1751**	1000	0.36801
**cgpGmo_S493**	1000	0.36585
**cgpGmo_S875b**	1000	0.36468
**cgpGmo_S876**	1000	0.363

**cgpGmo_S1853**	1000	0.35392
**cgpGmo_S57**	1000	0.35281
**cgpGmo_S405a**	1000	0.35205
**cgpGmo_S182**	1000	0.34884
**cgpGmo_S2277**	1000	0.3418
**cgpGmo_S2122**	1000	0.34124
**cgpGmo_S2104**	1000	0.33172
**cgpGmo_S852**	1000	0.33129
**cgpGmo_S1962**	1000	0.32491
**cgpGmo_S2082**	1000	0.31988
**cgpGmo_S536**	1000	0.31271
**cgpGmo_S184**	1000	0.31264
**cgpGmo_S985**	1000	0.31228
**cgpGmo_S116**	1000	0.30377
**cgpGmo_S510**	1000	0.30215
**cgpGmo_S1456**	1000	0.29915
**cgpGmo_S1543**	1000	0.29901
**cgpGmo_S1062**	1000	0.29897
**cgpGmo_S866**	1000	0.29728
**cgpGmo_S1467**	1000	0.29718
**cgpGmo_S1167**	1000	0.29703
**cgpGmo_S2186**	1000	0.29409
**cgpGmo_S603**	1000	0.29321
**cgpGmo_S755**	1000	0.29195
**cgpGmo_S1258a**	1000	0.29189
**cgpGmo_S2101**	1000	0.29127
**cgpGmo_S352**	1000	0.28934
**cgpGmo_S1046**	1000	0.28881
**cgpGmo_S975b**	1000	0.28862
**cgpGmo_S1703**	1000	0.28772
**cgpGmo_S174**	1000	0.28534
**cgpGmo_S1842**	1000	0.28187
**cgpGmo_S951b**	1000	0.28004
**cgpGmo_S248a**	1000	0.27993
**cgpGmo_S292b**	1000	0.27971
**cgpGmo_S2287**	1000	0.27859
**cgpGmo_S1905**	1000	0.27704
**cgpGmo_S183**	1000	0.27484
**cgpGmo_S1032**	1000	0.27348
**cgpGmo_S1242**	1000	0.27289
**cgpGmo_S584**	1000	0.27116
**cgpGmo_S1959**	1000	0.26735
**cgpGmo_S938**	1000	0.26733

**cgpGmo_S1945**	1000	0.2626
**cgpGmo_S986**	1000	0.26239
**cgpGmo_S1157**	1000	0.26214
**cgpGmo_S407**	1000	0.25994
**cgpGmo_S94**	1000	0.25929
**cgpGmo_S973**	1000	0.25907
**cgpGmo_S1209**	1000	0.25796
**cgpGmo_S1009**	1000	0.25637
**cgpGmo_S1111**	1000	0.25483
**cgpGmo_S636**	1000	0.25432
**cgpGmo_S1095**	1000	0.2538
**cgpGmo_S2015**	1000	0.25273
**cgpGmo_S1692**	1000	0.25065
**cgpGmo_S1938**	1000	0.24988
**cgpGmo_S342**	1000	0.24787
**cgpGmo_S1022**	1000	0.2467
**cgpGmo_S1712**	1000	0.24611
**cgpGmo_S49**	1000	0.24276
**cgpGmo_S1988**	1000	0.24258
**cgpGmo_S616**	1000	0.2416
**cgpGmo_S1896**	1000	0.24026
**cgpGmo_S1788**	1000	0.23863
**cgpGmo_S693**	1000	0.23692
**cgpGmo_S1474**	1000	0.23442
**cgpGmo_S727**	1000	0.23412
**cgpGmo_S390b**	1000	0.23158
**cgpGmo_S143**	1000	0.22442
**cgpGmo_S556**	3.6988	0.26032
**cgpGmo_S888**	3.6988	0.25002
**cgpGmo_S29**	3.6988	0.24
**cgpGmo_S2153**	3.6988	0.23709
**cgpGmo_S586**	3.6988	0.23598
**cgpGmo_S195**	3.6988	0.23456
**cgpGmo_S1538b**	3.6988	0.22975
**cgpGmo_S1294**	3.6988	0.22639
**cgpGmo_S430a**	3.3977	0.27325
**cgpGmo_S2126**	3.3977	0.23951
**cgpGmo_S207**	3.3977	0.23589
**cgpGmo_S688**	3.2215	0.27558
**cgpGmo_S68**	3.2215	0.23894
**cgpGmo_S1098**	3.2215	0.23304
**cgpGmo_S263**	3.2215	0.047243
**cgpGmo_S93**	3.2215	0.046124

**cgpGmo_S2200**	3.0965	0.25544
**cgpGmo_S1899**	3.0965	0.22037
**cgpGmo_S2242**	3.0965	0.2156
**cgpGmo_S1105**	3.0965	0.042351
**cgpGmo_S741**	2.9995	0.30231
**cgpGmo_S759**	2.9995	0.23394
**cgpGmo_S1706**	2.9995	0.23183
**cgpGmo_S196**	2.9995	0.22912
**cgpGmo_S1733**	2.9995	0.22169
**cgpGmo_S873**	2.9202	0.24186
**cgpGmo_S687**	2.8532	0.047513
**cgpGmo_S597**	2.7439	0.051932
**cgpGmo_S1483**	2.698	0.051868
**cgpGmo_S930**	2.6187	0.22971
**cgpGmo_S1011a**	2.5838	0.23957
**cgpGmo_S2171**	2.5838	0.051032
**cgpGmo_S283**	2.5515	0.24983
**cgpGmo_S474**	2.5515	0.24379
**cgpGmo_S1034**	2.5515	0.23093
**cgpGmo_S1202**	2.5515	0.21897
**cgpGmo_S1321**	2.5215	0.24629
**cgpGmo_S435**	2.5215	0.23921
**cgpGmo_S360**	2.5215	0.2276
**cgpGmo_S1906**	2.4934	0.22691
**cgpGmo_S1127**	2.4934	0.22179
**cgpGmo_S1721**	2.4934	0.050003
**cgpGmo_S1999**	2.467	0.23388
**cgpGmo_S1850**	2.467	0.054134
**cgpGmo_S282**	2.4185	0.22871
**cgpGmo_S1904**	2.4185	0.21467
**cgpGmo_S1435**	2.3961	0.22736
**cgpGmo_S793a**	2.3961	0.050755
**cgpGmo_S2209**	2.3352	0.056262
**cgpGmo_S1291**	2.3166	0.24621
**cgpGmo_S2288**	2.3166	0.23166
**cgpGmo_S982a**	2.2988	0.26188
**cgpGmo_S444**	2.2988	0.23749
**cgpGmo_S334**	2.2816	0.24931
**cgpGmo_S1279**	2.234	0.051598
**cgpGmo_S980**	2.2191	0.051908
**cgpGmo_S1801**	2.2048	0.23158
**cgpGmo_S594**	2.2048	0.053016
**cgpGmo_S1868**	2.1909	0.2493

**cgpGmo_S1646**	2.1909	0.053529
**cgpGmo_S1255b**	2.1775	0.23062
**cgpGmo_S291**	2.1644	0.2441

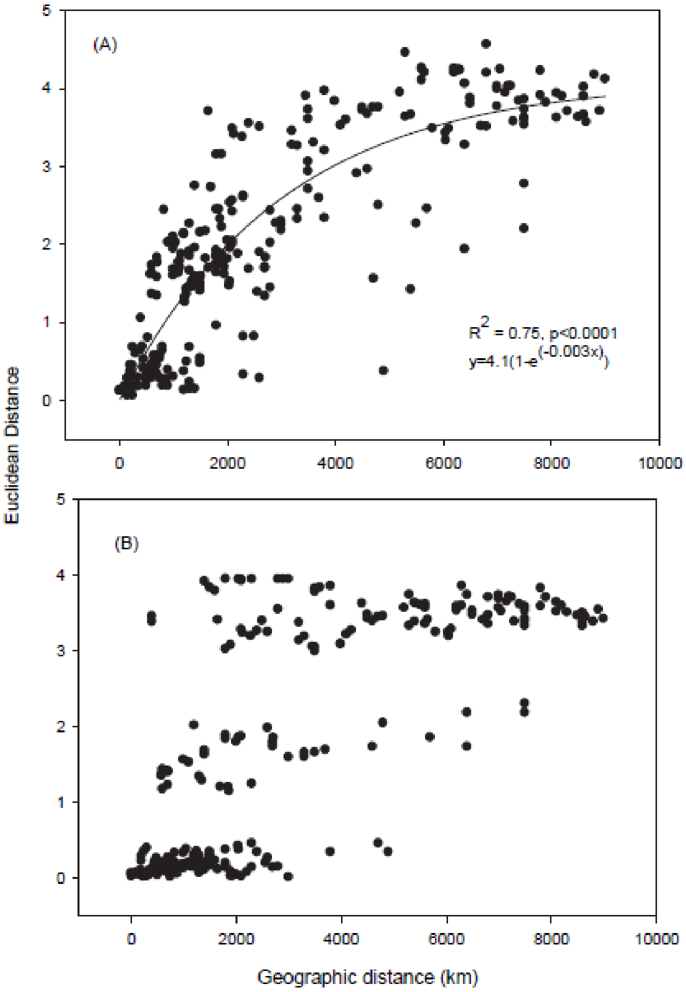

Figure S1. Euclidean distance among population average PCoA values of SNPs from range-wide samples of Atlantic cod using (A) all SNPs and (B) only neutral SNPs. See Figure 5 for PCoA and groups present.
