# Supplementary material for: Genomic islands of divergence and their consequences for the resolution of spatial structure in an exploited marine fish
Source: Evol Appl. 2013 Jan 21;6(3):450–61. doi: 10.1111/eva.12026 (PMC3673473; doi:10.1111/eva.12026)
Supplement: Data S2 — LGMAP1. [file eva0006-0450-sd2.pdf]

LGMAP1 Supplemental Information

CGPIA1

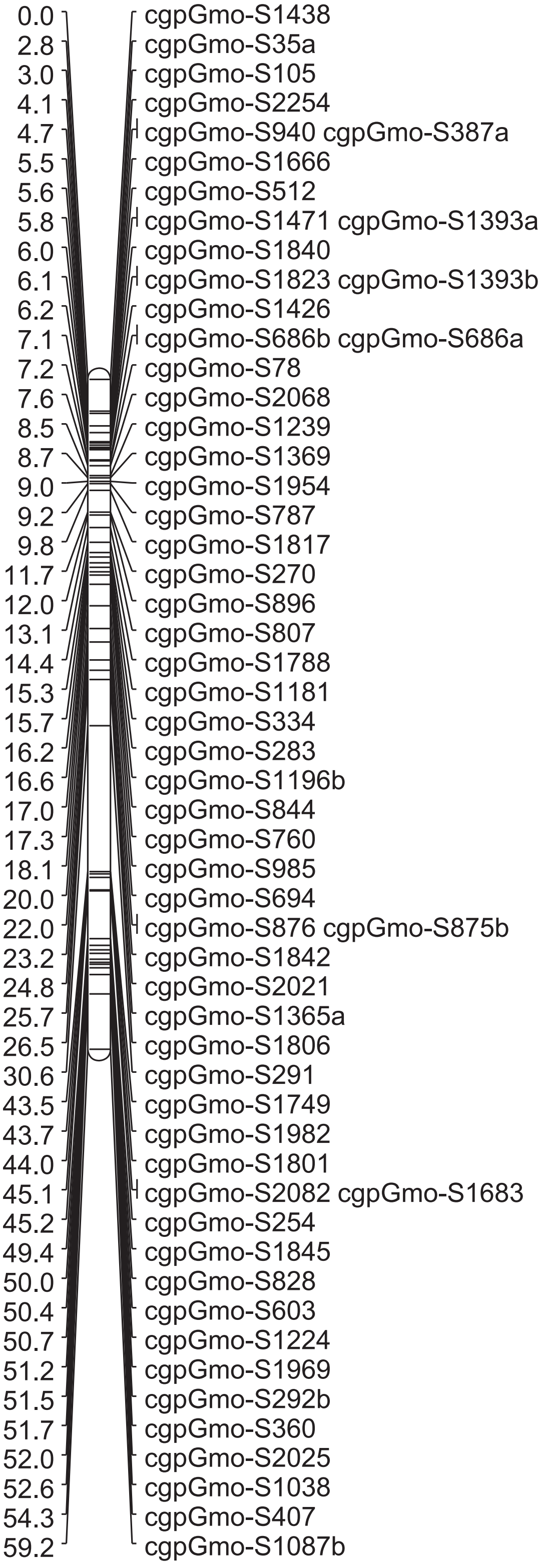

CGPIA2

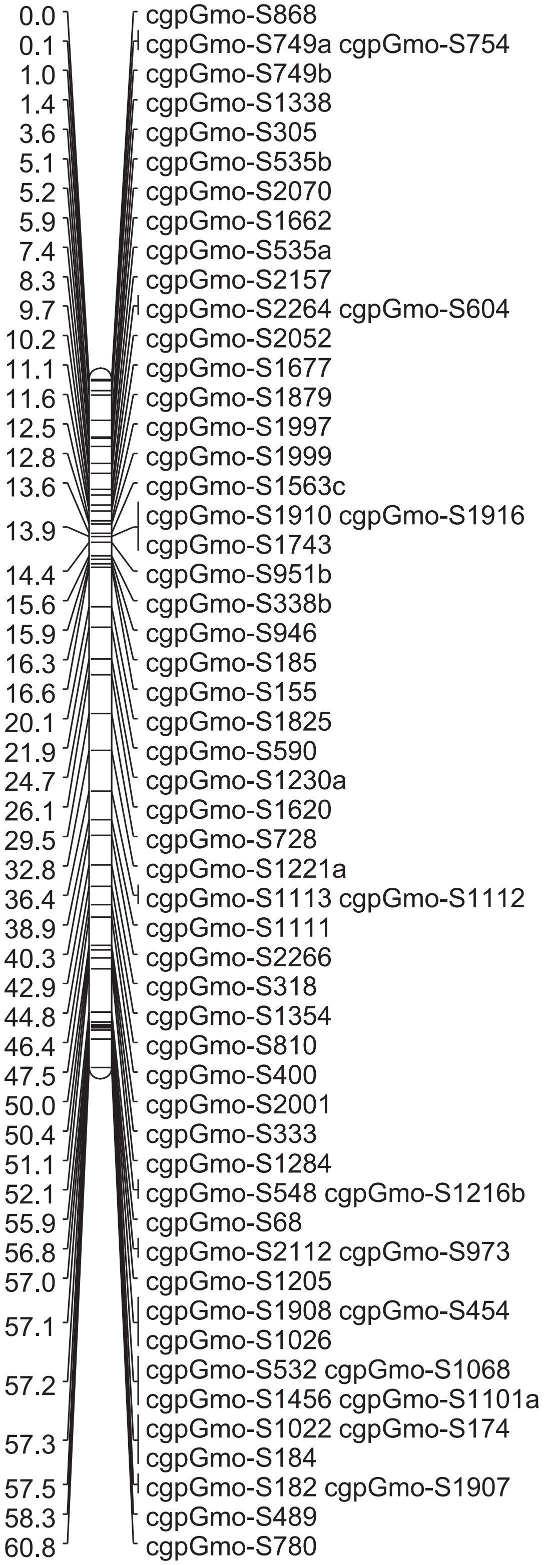

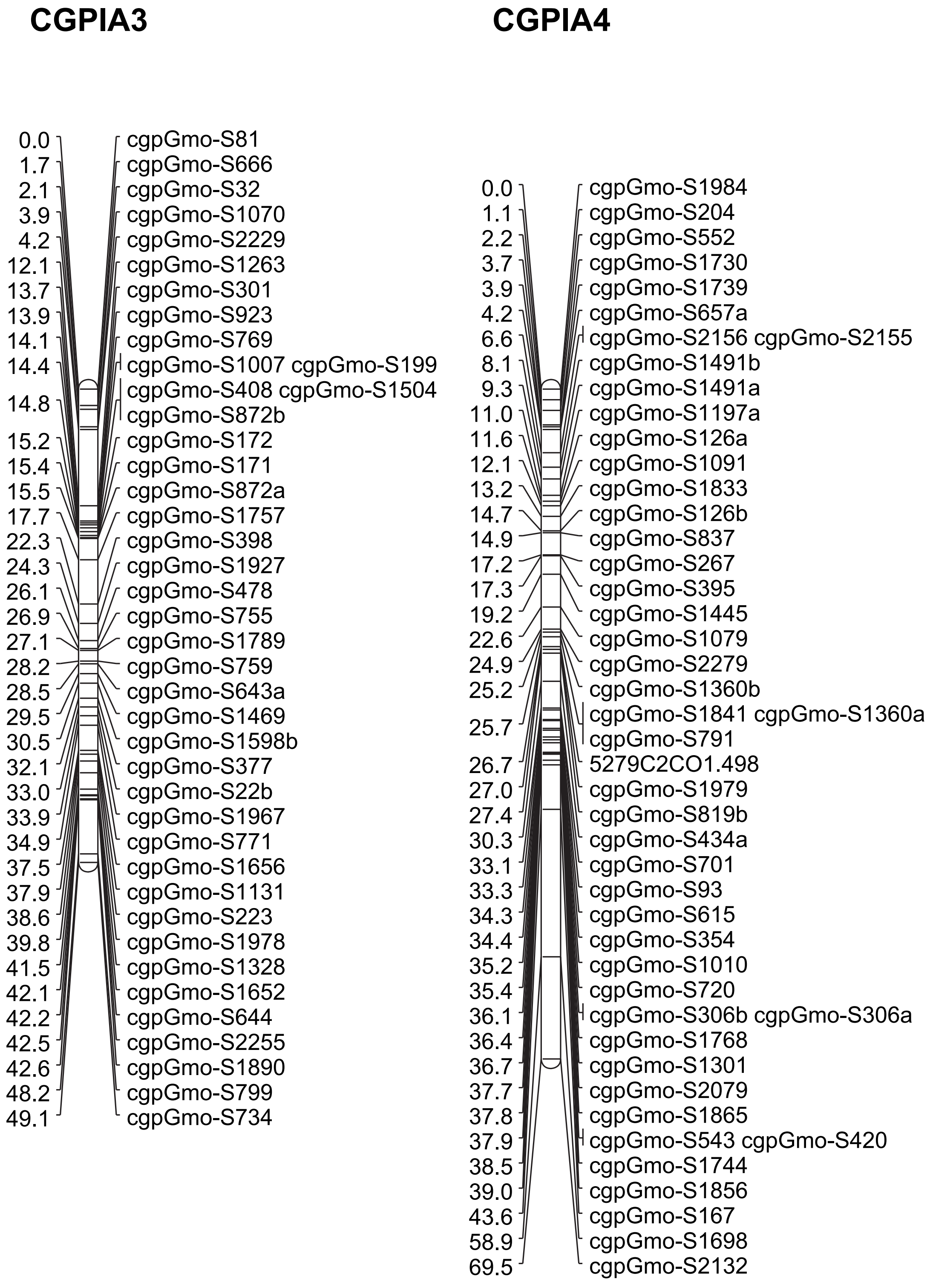
CGPIA3
0.0 cgpGmo-S81
1.7 cgpGmo-S666
2.1 cgpGmo-S32
3.9 cgpGmo-S1070
4.2 cgpGmo-S2229
12.1 cgpGmo-S1263
13.7 cgpGmo-S301
13.9 cgpGmo-S923
14.1 cgpGmo-S769
14.4 cgpGmo-S1007 cgpGmo-S199
14.8 cgpGmo-S408 cgpGmo-S1504
cgpGmo-S872b
15.2 cgpGmo-S172
15.4 cgpGmo-S171
15.5 cgpGmo-S872a
17.7 cgpGmo-S1757
22.3 cgpGmo-S398
24.3 cgpGmo-S1927
26.1 cgpGmo-S478
26.9 cgpGmo-S755
27.1 cgpGmo-S1789
28.2 cgpGmo-S759
28.5 cgpGmo-S643a
29.5 cgpGmo-S1469
30.5 cgpGmo-S1598b
32.1 cgpGmo-S377
33.0 cgpGmo-S22b
33.9 cgpGmo-S1967
34.9 cgpGmo-S771
37.5 cgpGmo-S1656
37.9 cgpGmo-S1131
38.6 cgpGmo-S223
39.8 cgpGmo-S1978
41.5 cgpGmo-S1328
42.1 cgpGmo-S1652
42.2 cgpGmo-S644
42.5 cgpGmo-S2255
42.6 cgpGmo-S1890
48.2 cgpGmo-S799
49.1 cgpGmo-S734
CGPIA4
0.0 cgpGmo-S1984
1.1 cgpGmo-S204
2.2 cgpGmo-S552
3.7 cgpGmo-S1730
3.9 cgpGmo-S1739
4.2 cgpGmo-S657a
6.6 cgpGmo-S2156 cgpGmo-S2155
8.1 cgpGmo-S1491b
9.3 cgpGmo-S1491a
11.0 cgpGmo-S1197a
11.6 cgpGmo-S126a
12.1 cgpGmo-S1091
13.2 cgpGmo-S1833
14.7 cgpGmo-S126b
14.9 cgpGmo-S837
17.2 cgpGmo-S267
17.3 cgpGmo-S395
19.2 cgpGmo-S1445
22.6 cgpGmo-S1079
24.9 cgpGmo-S2279
25.2 cgpGmo-S1360b
25.7 cgpGmo-S1841 cgpGmo-S1360a
cgpGmo-S791
26.7 5279C2CO1.498
27.0 cgpGmo-S1979
27.4 cgpGmo-S819b
30.3 cgpGmo-S434a
33.1 cgpGmo-S701
33.3 cgpGmo-S93
34.3 cgpGmo-S615
34.4 cgpGmo-S354
35.2 cgpGmo-S1010
35.4 cgpGmo-S720
36.1 cgpGmo-S306b cgpGmo-S306a
36.4 cgpGmo-S1768
36.7 cgpGmo-S1301
37.7 cgpGmo-S2079
37.8 cgpGmo-S1865
37.9 cgpGmo-S543 cgpGmo-S420
38.5 cgpGmo-S1744
39.0 cgpGmo-S1856
43.6 cgpGmo-S167
58.9 cgpGmo-S1698
69.5 cgpGmo-S2132

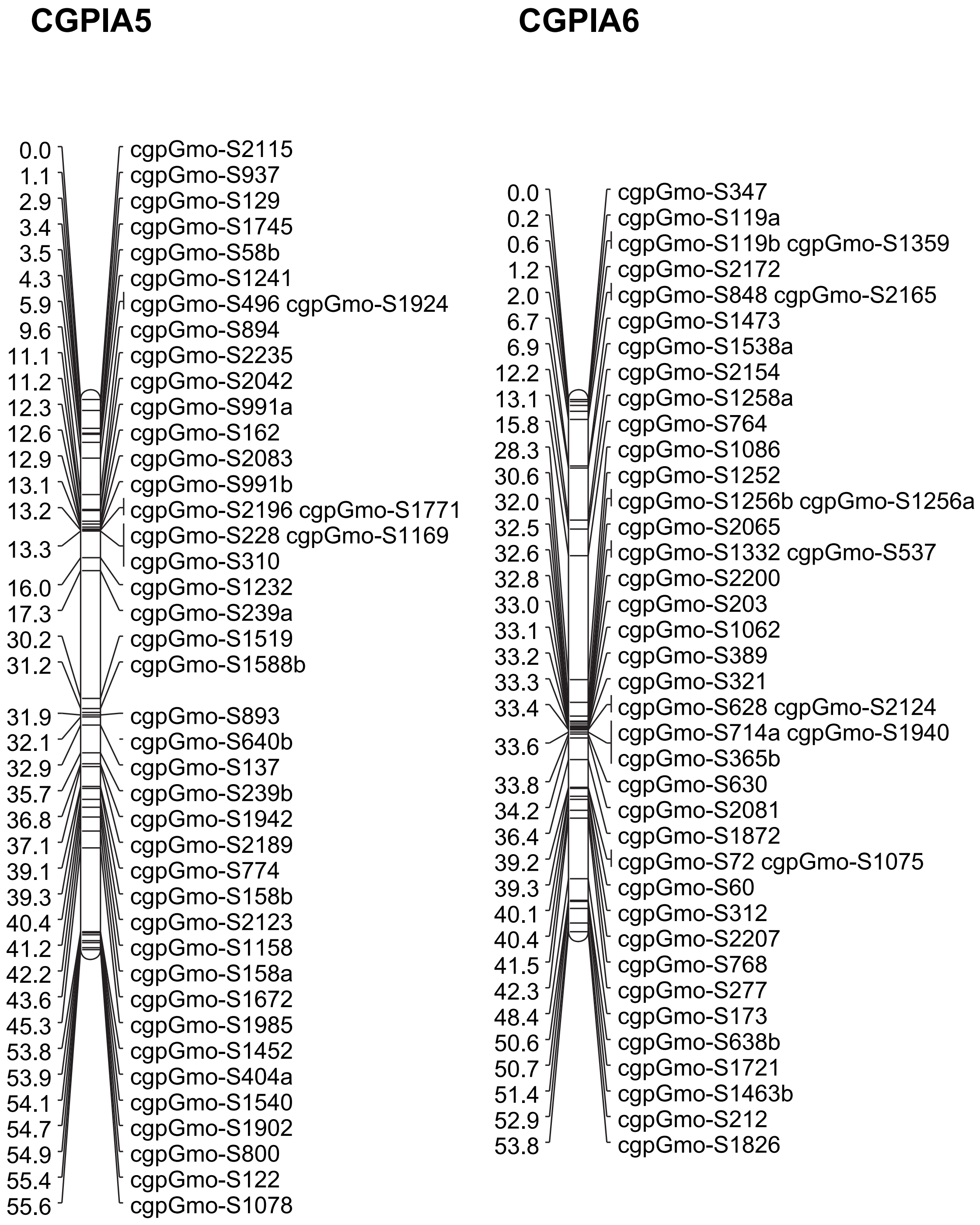
CGPIA5
0.0 cgpGmo-S2115
1.1 cgpGmo-S937
2.9 cgpGmo-S129
3.4 cgpGmo-S1745
3.5 cgpGmo-S58b
4.3 cgpGmo-S1241
5.9 cgpGmo-S496 cgpGmo-S1924
9.6 cgpGmo-S894
11.1 cgpGmo-S2235
11.2 cgpGmo-S2042
12.3 cgpGmo-S991a
12.6 cgpGmo-S162
12.9 cgpGmo-S2083
13.1 cgpGmo-S991b
13.2 cgpGmo-S2196 cgpGmo-S1771
13.3 cgpGmo-S228 cgpGmo-S1169
cgpGmo-S310
16.0 cgpGmo-S1232
17.3 cgpGmo-S239a
30.2 cgpGmo-S1519
31.2 cgpGmo-S1588b
31.9 cgpGmo-S893
32.1 cgpGmo-S640b
32.9 cgpGmo-S137
35.7 cgpGmo-S239b
36.8 cgpGmo-S1942
37.1 cgpGmo-S2189
39.1 cgpGmo-S774
39.3 cgpGmo-S158b
40.4 cgpGmo-S2123
41.2 cgpGmo-S1158
42.2 cgpGmo-S158a
43.6 cgpGmo-S1672
45.3 cgpGmo-S1985
53.8 cgpGmo-S1452
53.9 cgpGmo-S404a
54.1 cgpGmo-S1540
54.7 cgpGmo-S1902
54.9 cgpGmo-S800
55.4 cgpGmo-S122
55.6 cgpGmo-S1078
CGPIA6
0.0 cgpGmo-S347
0.2 cgpGmo-S119a
0.6 cgpGmo-S119b cgpGmo-S1359
1.2 cgpGmo-S2172
2.0 cgpGmo-S848 cgpGmo-S2165
6.7 cgpGmo-S1473
6.9 cgpGmo-S1538a
12.2 cgpGmo-S2154
13.1 cgpGmo-S1258a
15.8 cgpGmo-S764
28.3 cgpGmo-S1086
30.6 cgpGmo-S1252
32.0 cgpGmo-S1256b cgpGmo-S1256a
32.5 cgpGmo-S2065
32.6 cgpGmo-S1332 cgpGmo-S537
32.8 cgpGmo-S2200
33.0 cgpGmo-S203
33.1 cgpGmo-S1062
33.2 cgpGmo-S389
33.3 cgpGmo-S321
33.4 cgpGmo-S628 cgpGmo-S2124
33.6 cgpGmo-S714a cgpGmo-S1940
cgpGmo-S365b
33.8 cgpGmo-S630
34.2 cgpGmo-S2081
36.4 cgpGmo-S1872
39.2 cgpGmo-S72 cgpGmo-S1075
39.3 cgpGmo-S60
40.1 cgpGmo-S312
40.4 cgpGmo-S2207
41.5 cgpGmo-S768
42.3 cgpGmo-S277
48.4 cgpGmo-S173
50.6 cgpGmo-S638b
50.7 cgpGmo-S1721
51.4 cgpGmo-S1463b
52.9 cgpGmo-S212
53.8 cgpGmo-S1826

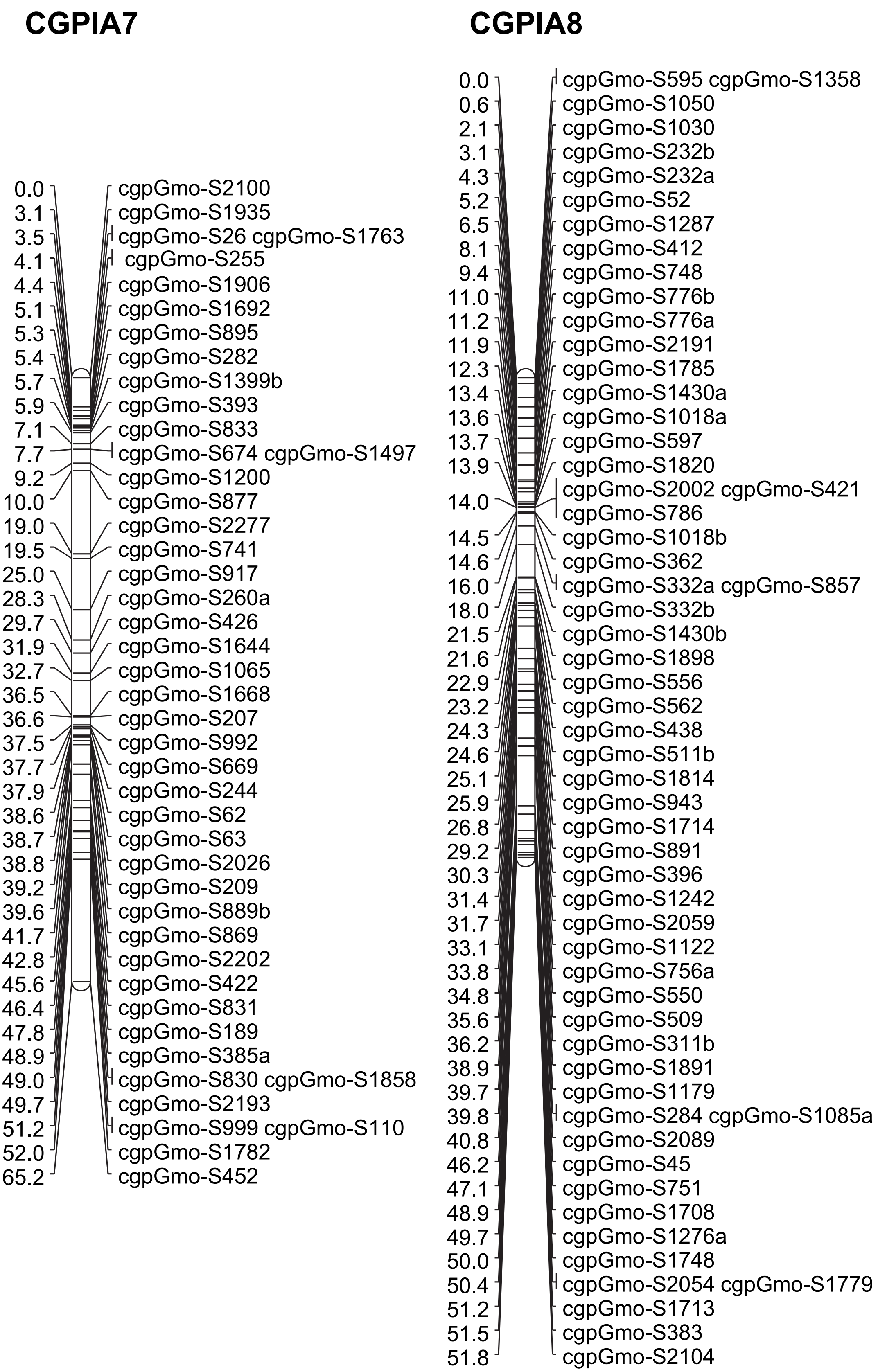
CGPIA7
0.0 cgpGmo-S2100
3.1 cgpGmo-S1935
3.5 cgpGmo-S26 cgpGmo-S1763
4.1 cgpGmo-S255
4.4 cgpGmo-S1906
5.1 cgpGmo-S1692
5.3 cgpGmo-S895
5.4 cgpGmo-S282
5.7 cgpGmo-S1399b
5.9 cgpGmo-S393
7.1 cgpGmo-S833
7.7 cgpGmo-S674 cgpGmo-S1497
9.2 cgpGmo-S1200
10.0 cgpGmo-S877
19.0 cgpGmo-S2277
19.5 cgpGmo-S741
25.0 cgpGmo-S917
28.3 cgpGmo-S260a
29.7 cgpGmo-S426
31.9 cgpGmo-S1644
32.7 cgpGmo-S1065
36.5 cgpGmo-S1668
36.6 cgpGmo-S207
37.5 cgpGmo-S992
37.7 cgpGmo-S669
37.9 cgpGmo-S244
38.6 cgpGmo-S62
38.7 cgpGmo-S63
38.8 cgpGmo-S2026
39.2 cgpGmo-S209
39.6 cgpGmo-S889b
41.7 cgpGmo-S869
42.8 cgpGmo-S2202
45.6 cgpGmo-S422
46.4 cgpGmo-S831
47.8 cgpGmo-S189
48.9 cgpGmo-S385a
49.0 cgpGmo-S830 cgpGmo-S1858
49.7 cgpGmo-S2193
51.2 cgpGmo-S999 cgpGmo-S110
52.0 cgpGmo-S1782
65.2 cgpGmo-S452
CGPIA8
0.0 cgpGmo-S595 cgpGmo-S1358
0.6 cgpGmo-S1050
2.1 cgpGmo-S1030
3.1 cgpGmo-S232b
4.3 cgpGmo-S232a
5.2 cgpGmo-S52
6.5 cgpGmo-S1287
8.1 cgpGmo-S412
9.4 cgpGmo-S748
11.0 cgpGmo-S776b
11.2 cgpGmo-S776a
11.9 cgpGmo-S2191
12.3 cgpGmo-S1785
13.4 cgpGmo-S1430a
13.6 cgpGmo-S1018a
13.7 cgpGmo-S597
13.9 cgpGmo-S1820
14.0 cgpGmo-S2002 cgpGmo-S421
cgpGmo-S786
14.5 cgpGmo-S1018b
14.6 cgpGmo-S362
16.0 cgpGmo-S332a cgpGmo-S857
18.0 cgpGmo-S332b
21.5 cgpGmo-S1430b
21.6 cgpGmo-S1898
22.9 cgpGmo-S556
23.2 cgpGmo-S562
24.3 cgpGmo-S438
24.6 cgpGmo-S511b
25.1 cgpGmo-S1814
25.9 cgpGmo-S943
26.8 cgpGmo-S1714
29.2 cgpGmo-S891
30.3 cgpGmo-S396
31.4 cgpGmo-S1242
31.7 cgpGmo-S2059
33.1 cgpGmo-S1122
33.8 cgpGmo-S756a
34.8 cgpGmo-S550
35.6 cgpGmo-S509
36.2 cgpGmo-S311b
38.9 cgpGmo-S1891
39.7 cgpGmo-S1179
39.8 cgpGmo-S284 cgpGmo-S1085a
40.8 cgpGmo-S2089
46.2 cgpGmo-S45
47.1 cgpGmo-S751
48.9 cgpGmo-S1708
49.7 cgpGmo-S1276a
50.0 cgpGmo-S1748
50.4 cgpGmo-S2054 cgpGmo-S1779
51.2 cgpGmo-S1713
51.5 cgpGmo-S383
51.8 cgpGmo-S2104

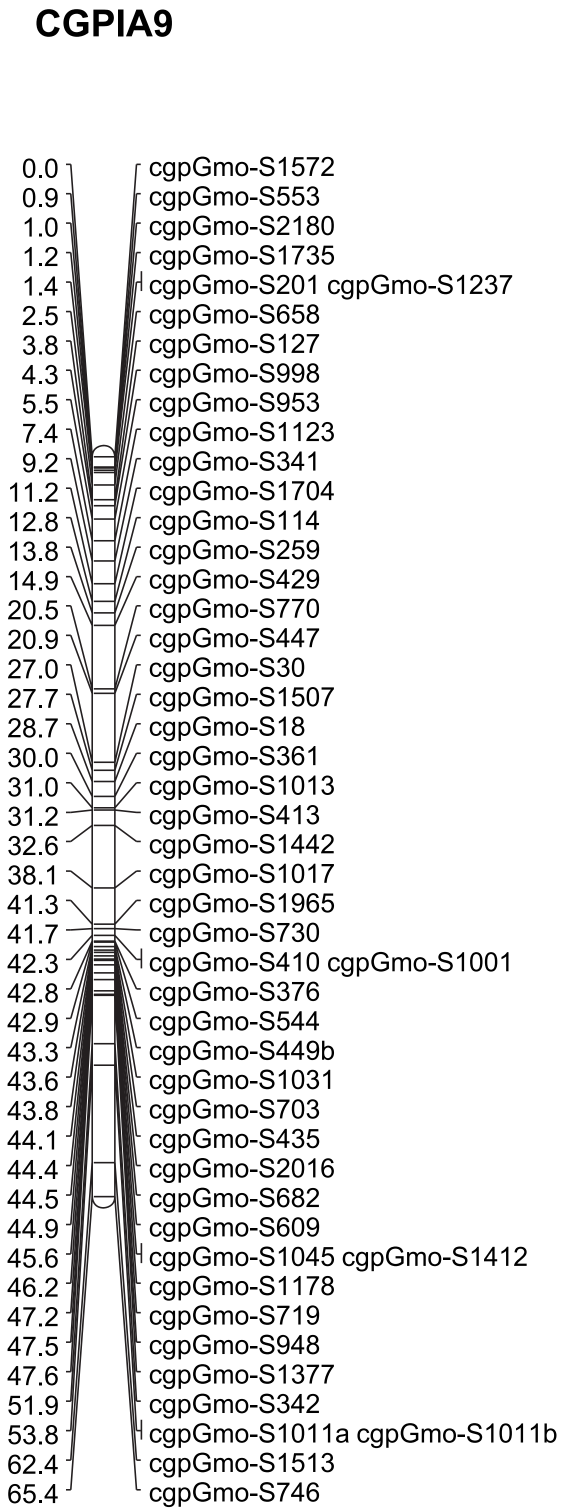
CGPIA9
0.0 cgpGmo-S1572
0.9 cgpGmo-S553
1.0 cgpGmo-S2180
1.2 cgpGmo-S1735
1.4 cgpGmo-S201 cgpGmo-S1237
2.5 cgpGmo-S658
3.8 cgpGmo-S127
4.3 cgpGmo-S998
5.5 cgpGmo-S953
7.4 cgpGmo-S1123
9.2 cgpGmo-S341
11.2 cgpGmo-S1704
12.8 cgpGmo-S114
13.8 cgpGmo-S259
14.9 cgpGmo-S429
20.5 cgpGmo-S770
20.9 cgpGmo-S447
27.0 cgpGmo-S30
27.7 cgpGmo-S1507
28.7 cgpGmo-S18
30.0 cgpGmo-S361
31.0 cgpGmo-S1013
31.2 cgpGmo-S413
32.6 cgpGmo-S1442
38.1 cgpGmo-S1017
41.3 cgpGmo-S1965
41.7 cgpGmo-S730
42.3 cgpGmo-S410 cgpGmo-S1001
42.8 cgpGmo-S376
42.9 cgpGmo-S544
43.3 cgpGmo-S449b
43.6 cgpGmo-S1031
43.8 cgpGmo-S703
44.1 cgpGmo-S435
44.4 cgpGmo-S2016
44.5 cgpGmo-S682
44.9 cgpGmo-S609
45.6 cgpGmo-S1045 cgpGmo-S1412
46.2 cgpGmo-S1178
47.2 cgpGmo-S719
47.5 cgpGmo-S948
47.6 cgpGmo-S1377
51.9 cgpGmo-S342
53.8 cgpGmo-S1011a cgpGmo-S1011b
62.4 cgpGmo-S1513
65.4 cgpGmo-S746

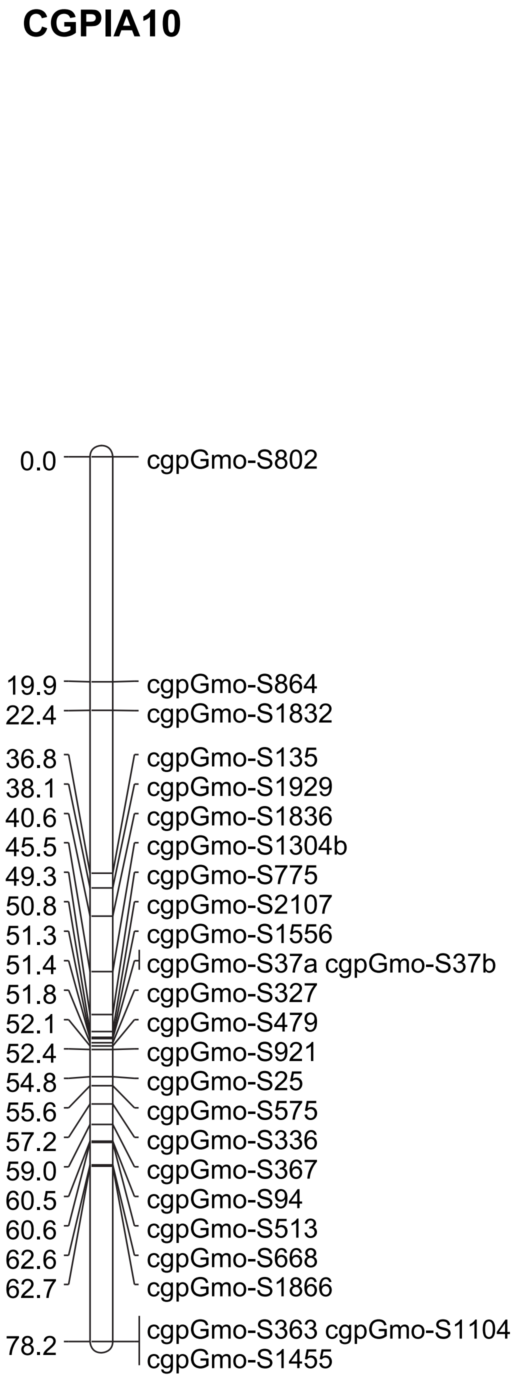
CGPIA10
0.0 cgpGmo-S802
19.9 cgpGmo-S864
22.4 cgpGmo-S1832
36.8 cgpGmo-S135
38.1 cgpGmo-S1929
40.6 cgpGmo-S1836
45.5 cgpGmo-S1304b
49.3 cgpGmo-S775
50.8 cgpGmo-S2107
51.3 cgpGmo-S1556
51.4 cgpGmo-S37a cgpGmo-S37b
51.8 cgpGmo-S327
52.1 cgpGmo-S479
52.4 cgpGmo-S921
54.8 cgpGmo-S25
55.6 cgpGmo-S575
57.2 cgpGmo-S336
59.0 cgpGmo-S367
60.5 cgpGmo-S94
60.6 cgpGmo-S513
62.6 cgpGmo-S668
62.7 cgpGmo-S1866
78.2 cgpGmo-S363 cgpGmo-S1104
cgpGmo-S1455

CGPIA11 CGPIA12

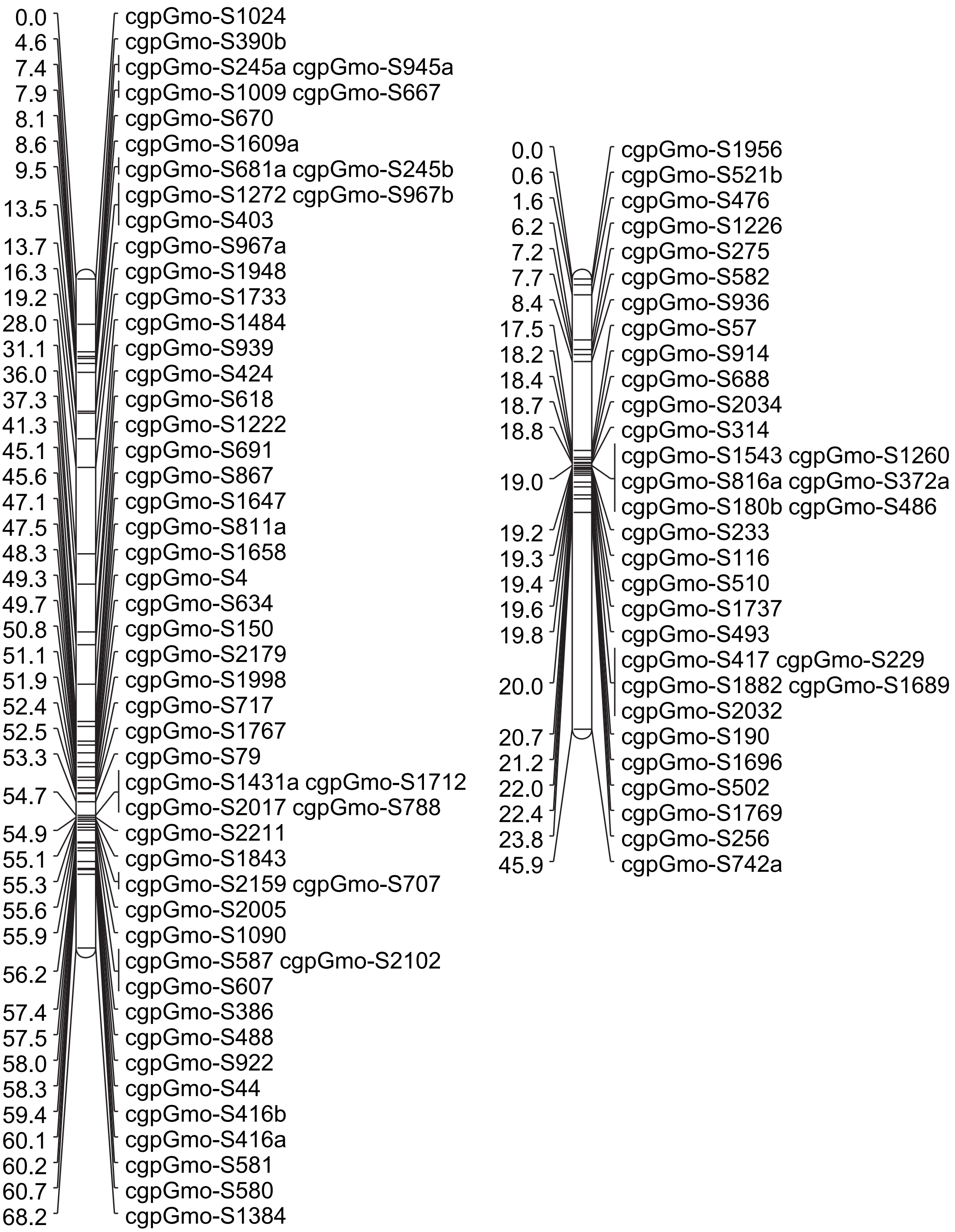

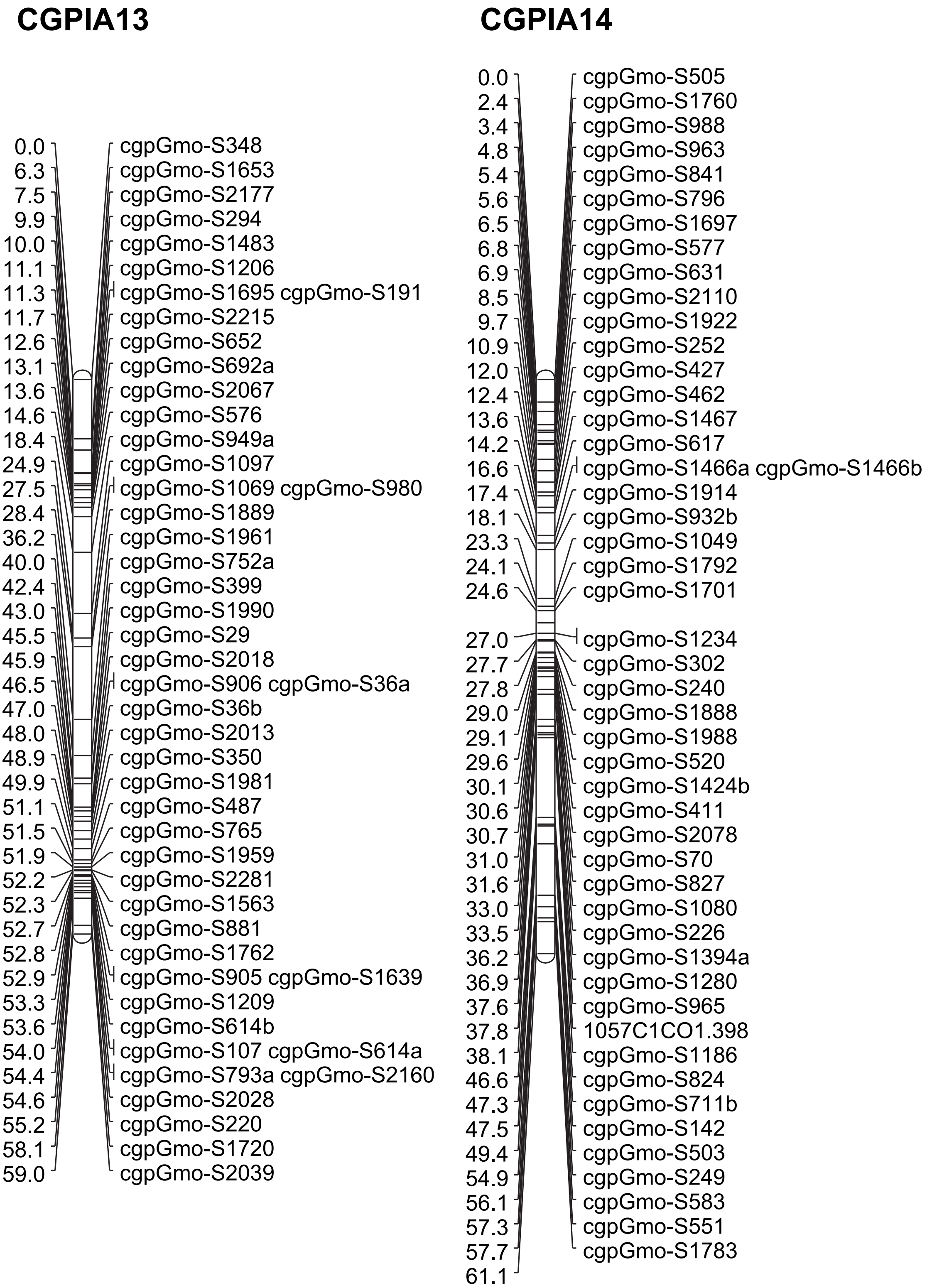

CGPIA13
0.0 cgpGmo-S348
6.3 cgpGmo-S1653
7.5 cgpGmo-S2177
9.9 cgpGmo-S294
10.0 cgpGmo-S1483
11.1 cgpGmo-S1206
11.3 cgpGmo-S1695 cgpGmo-S191
11.7 cgpGmo-S2215
12.6 cgpGmo-S652
13.1 cgpGmo-S692a
13.6 cgpGmo-S2067
14.6 cgpGmo-S576
18.4 cgpGmo-S949a
24.9 cgpGmo-S1097
27.5 cgpGmo-S1069 cgpGmo-S980
28.4 cgpGmo-S1889
36.2 cgpGmo-S1961
40.0 cgpGmo-S752a
42.4 cgpGmo-S399
43.0 cgpGmo-S1990
45.5 cgpGmo-S29
45.9 cgpGmo-S2018
46.5 cgpGmo-S906 cgpGmo-S36a
47.0 cgpGmo-S36b
48.0 cgpGmo-S2013
48.9 cgpGmo-S350
49.9 cgpGmo-S1981
51.1 cgpGmo-S487
51.5 cgpGmo-S765
51.9 cgpGmo-S1959
52.2 cgpGmo-S2281
52.3 cgpGmo-S1563
52.7 cgpGmo-S881
52.8 cgpGmo-S1762
52.9 cgpGmo-S905 cgpGmo-S1639
53.3 cgpGmo-S1209
53.6 cgpGmo-S614b
54.0 cgpGmo-S107 cgpGmo-S614a
54.4 cgpGmo-S793a cgpGmo-S2160
54.6 cgpGmo-S2028
55.2 cgpGmo-S220
58.1 cgpGmo-S1720
59.0 cgpGmo-S2039
CGPIA14
0.0 cgpGmo-S505
2.4 cgpGmo-S1760
3.4 cgpGmo-S988
4.8 cgpGmo-S963
5.4 cgpGmo-S841
5.6 cgpGmo-S796
6.5 cgpGmo-S1697
6.8 cgpGmo-S577
6.9 cgpGmo-S631
8.5 cgpGmo-S2110
9.7 cgpGmo-S1922
10.9 cgpGmo-S252
12.0 cgpGmo-S427
12.4 cgpGmo-S462
13.6 cgpGmo-S1467
14.2 cgpGmo-S617
16.6 cgpGmo-S1466a cgpGmo-S1466b
17.4 cgpGmo-S1914
18.1 cgpGmo-S932b
23.3 cgpGmo-S1049
24.1 cgpGmo-S1792
24.6 cgpGmo-S1701
27.0 cgpGmo-S1234
27.7 cgpGmo-S302
27.8 cgpGmo-S240
29.0 cgpGmo-S1888
29.1 cgpGmo-S1988
29.6 cgpGmo-S520
30.1 cgpGmo-S1424b
30.6 cgpGmo-S411
30.7 cgpGmo-S2078
31.0 cgpGmo-S70
31.6 cgpGmo-S827
33.0 cgpGmo-S1080
33.5 cgpGmo-S226
36.2 cgpGmo-S1394a
36.9 cgpGmo-S1280
37.6 cgpGmo-S965
37.8 1057C1CO1.398
38.1 cgpGmo-S1186
46.6 cgpGmo-S824
47.3 cgpGmo-S711b
47.5 cgpGmo-S142
49.4 cgpGmo-S503
54.9 cgpGmo-S249
56.1 cgpGmo-S583
57.3 cgpGmo-S551
57.7 cgpGmo-S1783
61.1

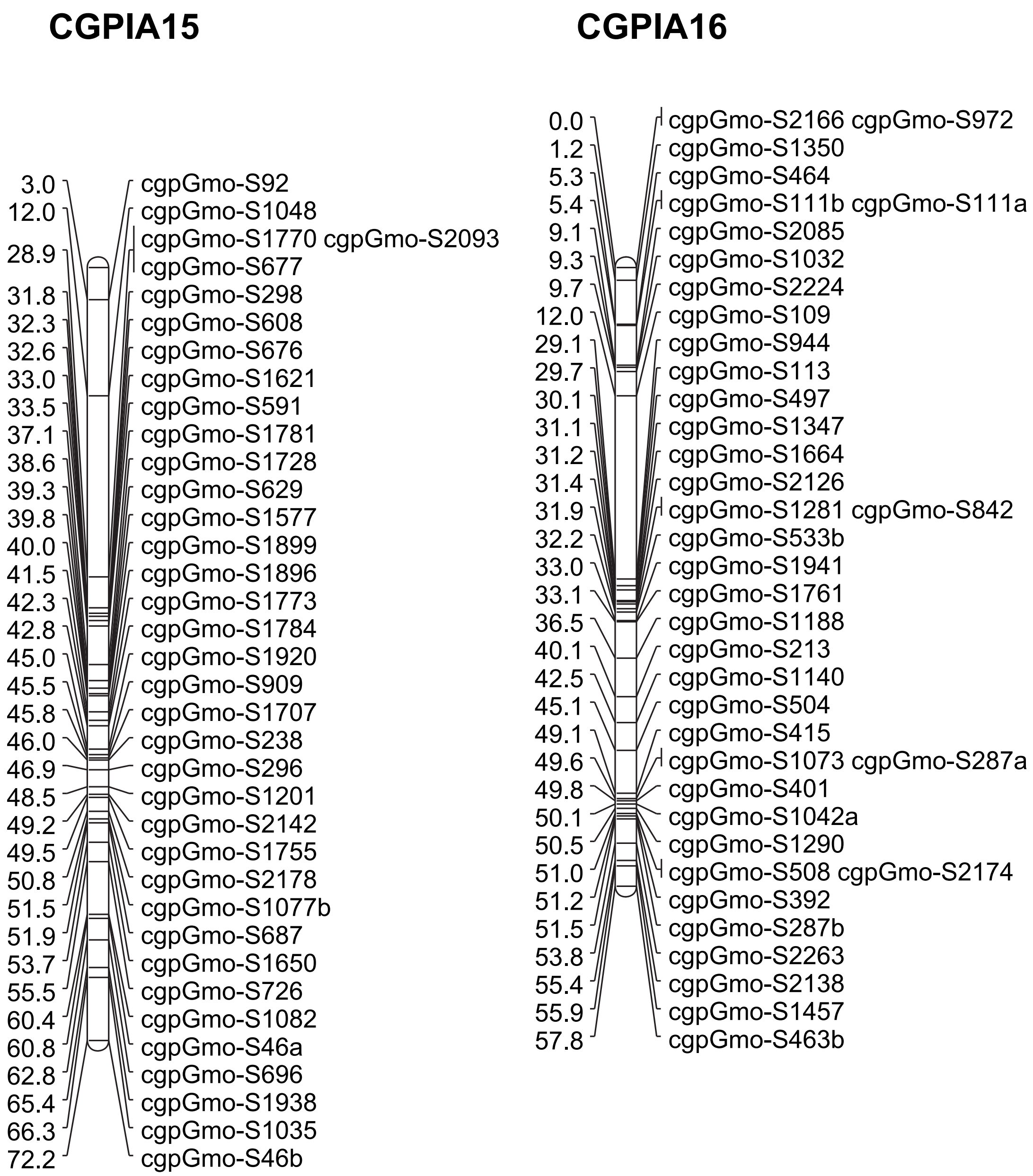
CGPIA15
3.0 cgpGmo-S92
12.0 cgpGmo-S1048
28.9 cgpGmo-S1770 cgpGmo-S2093
cgpGmo-S677
31.8 cgpGmo-S298
32.3 cgpGmo-S608
32.6 cgpGmo-S676
33.0 cgpGmo-S1621
33.5 cgpGmo-S591
37.1 cgpGmo-S1781
38.6 cgpGmo-S1728
39.3 cgpGmo-S629
39.8 cgpGmo-S1577
40.0 cgpGmo-S1899
41.5 cgpGmo-S1896
42.3 cgpGmo-S1773
42.8 cgpGmo-S1784
45.0 cgpGmo-S1920
45.5 cgpGmo-S909
45.8 cgpGmo-S1707
46.0 cgpGmo-S238
46.9 cgpGmo-S296
48.5 cgpGmo-S1201
49.2 cgpGmo-S2142
49.5 cgpGmo-S1755
50.8 cgpGmo-S2178
51.5 cgpGmo-S1077b
51.9 cgpGmo-S687
53.7 cgpGmo-S1650
55.5 cgpGmo-S726
60.4 cgpGmo-S1082
60.8 cgpGmo-S46a
62.8 cgpGmo-S696
65.4 cgpGmo-S1938
66.3 cgpGmo-S1035
72.2 cgpGmo-S46b
CGPIA16
0.0 cgpGmo-S2166 cgpGmo-S972
1.2 cgpGmo-S1350
5.3 cgpGmo-S464
5.4 cgpGmo-S111b cgpGmo-S111a
9.1 cgpGmo-S2085
9.3 cgpGmo-S1032
9.7 cgpGmo-S2224
12.0 cgpGmo-S109
29.1 cgpGmo-S944
29.7 cgpGmo-S113
30.1 cgpGmo-S497
31.1 cgpGmo-S1347
31.2 cgpGmo-S1664
31.4 cgpGmo-S2126
31.9 cgpGmo-S1281 cgpGmo-S842
32.2 cgpGmo-S533b
33.0 cgpGmo-S1941
33.1 cgpGmo-S1761
36.5 cgpGmo-S1188
40.1 cgpGmo-S213
42.5 cgpGmo-S1140
45.1 cgpGmo-S504
49.1 cgpGmo-S415
49.6 cgpGmo-S1073 cgpGmo-S287a
49.8 cgpGmo-S401
50.1 cgpGmo-S1042a
50.5 cgpGmo-S1290
51.0 cgpGmo-S508 cgpGmo-S2174
51.2 cgpGmo-S392
51.5 cgpGmo-S287b
53.8 cgpGmo-S2263
55.4 cgpGmo-S2138
55.9 cgpGmo-S1457
57.8 cgpGmo-S463b

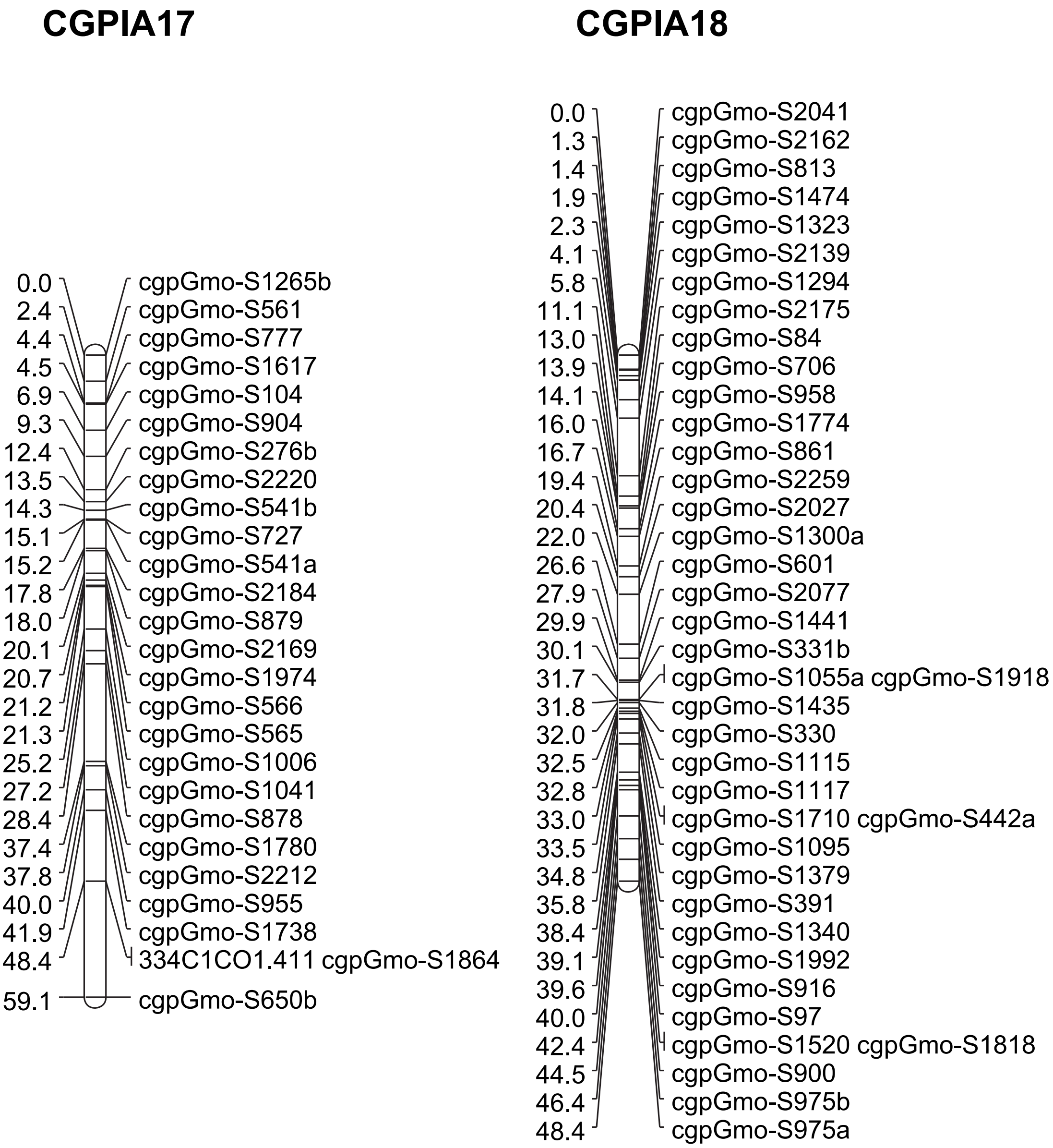
CGPIA17
0.0 cgpGmo-S1265b
2.4 cgpGmo-S561
4.4 cgpGmo-S777
4.5 cgpGmo-S1617
6.9 cgpGmo-S104
9.3 cgpGmo-S904
12.4 cgpGmo-S276b
13.5 cgpGmo-S2220
14.3 cgpGmo-S541b
15.1 cgpGmo-S727
15.2 cgpGmo-S541a
17.8 cgpGmo-S2184
18.0 cgpGmo-S879
20.1 cgpGmo-S2169
20.7 cgpGmo-S1974
21.2 cgpGmo-S566
21.3 cgpGmo-S565
25.2 cgpGmo-S1006
27.2 cgpGmo-S1041
28.4 cgpGmo-S878
37.4 cgpGmo-S1780
37.8 cgpGmo-S2212
40.0 cgpGmo-S955
41.9 cgpGmo-S1738
48.4 334C1CO1.411 cgpGmo-S1864
59.1 cgpGmo-S650b
CGPIA18
0.0 cgpGmo-S2041
1.3 cgpGmo-S2162
1.4 cgpGmo-S813
1.9 cgpGmo-S1474
2.3 cgpGmo-S1323
4.1 cgpGmo-S2139
5.8 cgpGmo-S1294
11.1 cgpGmo-S2175
13.0 cgpGmo-S84
13.9 cgpGmo-S706
14.1 cgpGmo-S958
16.0 cgpGmo-S1774
16.7 cgpGmo-S861
19.4 cgpGmo-S2259
20.4 cgpGmo-S2027
22.0 cgpGmo-S1300a
26.6 cgpGmo-S601
27.9 cgpGmo-S2077
29.9 cgpGmo-S1441
30.1 cgpGmo-S331b
31.7 cgpGmo-S1055a cgpGmo-S1918
31.8 cgpGmo-S1435
32.0 cgpGmo-S330
32.5 cgpGmo-S1115
32.8 cgpGmo-S1117
33.0 cgpGmo-S1710 cgpGmo-S442a
33.5 cgpGmo-S1095
34.8 cgpGmo-S1379
35.8 cgpGmo-S391
38.4 cgpGmo-S1340
39.1 cgpGmo-S1992
39.6 cgpGmo-S916
40.0 cgpGmo-S97
42.4 cgpGmo-S1520 cgpGmo-S1818
44.5 cgpGmo-S900
46.4 cgpGmo-S975b
48.4 cgpGmo-S975a

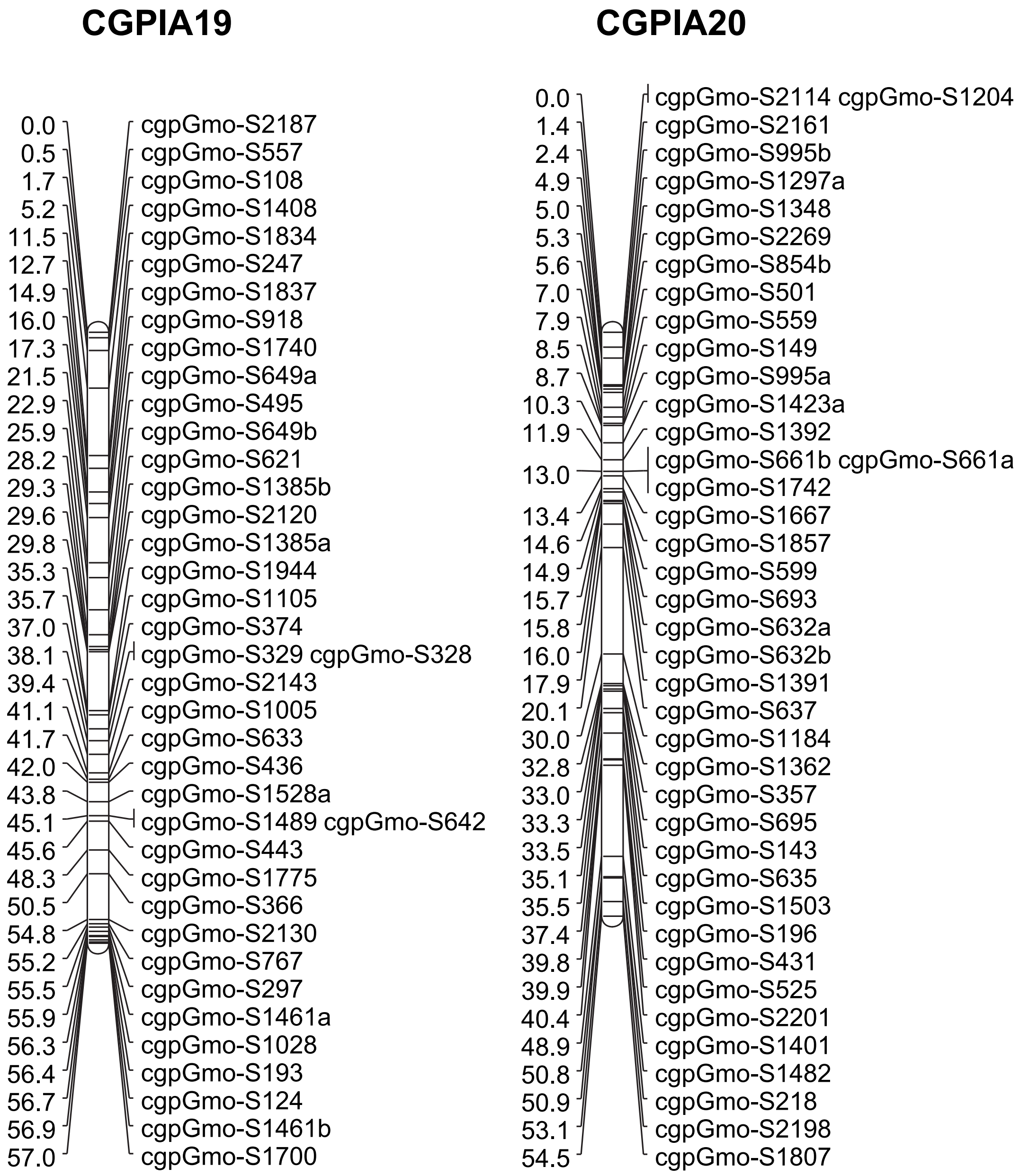
CGPIA19
0.0 cgpGmo-S2187
0.5 cgpGmo-S557
1.7 cgpGmo-S108
5.2 cgpGmo-S1408
11.5 cgpGmo-S1834
12.7 cgpGmo-S247
14.9 cgpGmo-S1837
16.0 cgpGmo-S918
17.3 cgpGmo-S1740
21.5 cgpGmo-S649a
22.9 cgpGmo-S495
25.9 cgpGmo-S649b
28.2 cgpGmo-S621
29.3 cgpGmo-S1385b
29.6 cgpGmo-S2120
29.8 cgpGmo-S1385a
35.3 cgpGmo-S1944
35.7 cgpGmo-S1105
37.0 cgpGmo-S374
38.1 cgpGmo-S329 cgpGmo-S328
39.4 cgpGmo-S2143
41.1 cgpGmo-S1005
41.7 cgpGmo-S633
42.0 cgpGmo-S436
43.8 cgpGmo-S1528a
45.1 cgpGmo-S1489 cgpGmo-S642
45.6 cgpGmo-S443
48.3 cgpGmo-S1775
50.5 cgpGmo-S366
54.8 cgpGmo-S2130
55.2 cgpGmo-S767
55.5 cgpGmo-S297
55.9 cgpGmo-S1461a
56.3 cgpGmo-S1028
56.4 cgpGmo-S193
56.7 cgpGmo-S124
56.9 cgpGmo-S1461b
57.0 cgpGmo-S1700
CGPIA20
0.0 cgpGmo-S2114 cgpGmo-S1204
1.4 cgpGmo-S2161
2.4 cgpGmo-S995b
4.9 cgpGmo-S1297a
5.0 cgpGmo-S1348
5.3 cgpGmo-S2269
5.6 cgpGmo-S854b
7.0 cgpGmo-S501
7.9 cgpGmo-S559
8.5 cgpGmo-S149
8.7 cgpGmo-S995a
10.3 cgpGmo-S1423a
11.9 cgpGmo-S1392
13.0 cgpGmo-S661b cgpGmo-S661a
cgpGmo-S1742
13.4 cgpGmo-S1667
14.6 cgpGmo-S1857
14.9 cgpGmo-S599
15.7 cgpGmo-S693
15.8 cgpGmo-S632a
16.0 cgpGmo-S632b
17.9 cgpGmo-S1391
20.1 cgpGmo-S637
30.0 cgpGmo-S1184
32.8 cgpGmo-S1362
33.0 cgpGmo-S357
33.3 cgpGmo-S695
33.5 cgpGmo-S143
35.1 cgpGmo-S635
35.5 cgpGmo-S1503
37.4 cgpGmo-S196
39.8 cgpGmo-S431
39.9 cgpGmo-S525
40.4 cgpGmo-S2201
48.9 cgpGmo-S1401
50.8 cgpGmo-S1482
50.9 cgpGmo-S218
53.1 cgpGmo-S2198
54.5 cgpGmo-S1807

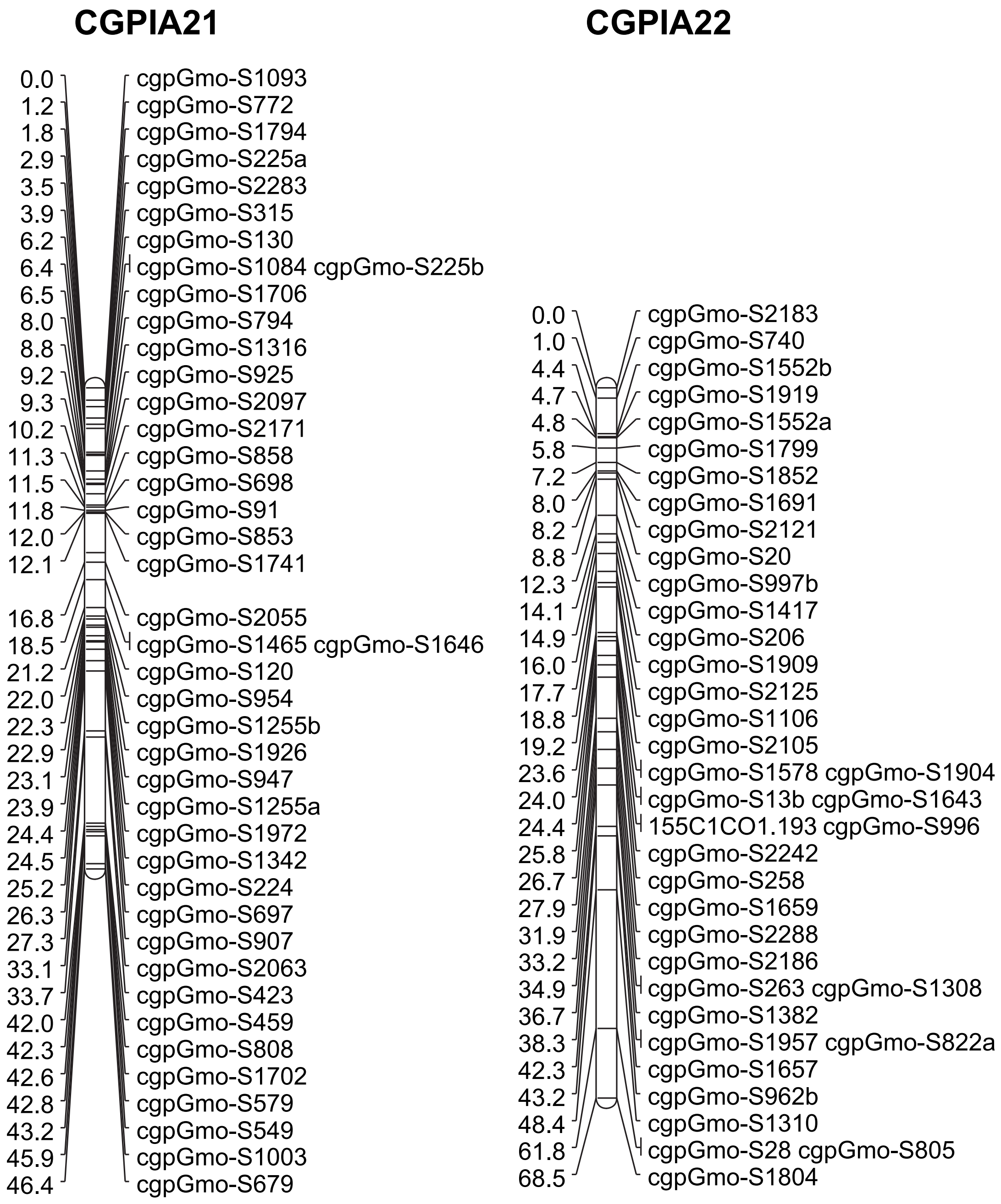

CGPIA21
0.0 cgpGmo-S1093
1.2 cgpGmo-S772
1.8 cgpGmo-S1794
2.9 cgpGmo-S225a
3.5 cgpGmo-S2283
3.9 cgpGmo-S315
6.2 cgpGmo-S130
6.4 cgpGmo-S1084 cgpGmo-S225b
6.5 cgpGmo-S1706
8.0 cgpGmo-S794
8.8 cgpGmo-S1316
9.2 cgpGmo-S925
9.3 cgpGmo-S2097
10.2 cgpGmo-S2171
11.3 cgpGmo-S858
11.5 cgpGmo-S698
11.8 cgpGmo-S91
12.0 cgpGmo-S853
12.1 cgpGmo-S1741
16.8 cgpGmo-S2055
18.5 cgpGmo-S1465 cgpGmo-S1646
21.2 cgpGmo-S120
22.0 cgpGmo-S954
22.3 cgpGmo-S1255b
22.9 cgpGmo-S1926
23.1 cgpGmo-S947
23.9 cgpGmo-S1255a
24.4 cgpGmo-S1972
24.5 cgpGmo-S1342
25.2 cgpGmo-S224
26.3 cgpGmo-S697
27.3 cgpGmo-S907
33.1 cgpGmo-S2063
33.7 cgpGmo-S423
42.0 cgpGmo-S459
42.3 cgpGmo-S808
42.6 cgpGmo-S1702
42.8 cgpGmo-S579
43.2 cgpGmo-S549
45.9 cgpGmo-S1003
46.4 cgpGmo-S679
CGPIA22
0.0 cgpGmo-S2183
1.0 cgpGmo-S740
4.4 cgpGmo-S1552b
4.7 cgpGmo-S1919
4.8 cgpGmo-S1552a
5.8 cgpGmo-S1799
7.2 cgpGmo-S1852
8.0 cgpGmo-S1691
8.2 cgpGmo-S2121
8.8 cgpGmo-S20
12.3 cgpGmo-S997b
14.1 cgpGmo-S1417
14.9 cgpGmo-S206
16.0 cgpGmo-S1909
17.7 cgpGmo-S2125
18.8 cgpGmo-S1106
19.2 cgpGmo-S2105
23.6 cgpGmo-S1578 cgpGmo-S1904
24.0 cgpGmo-S13b cgpGmo-S1643
24.4 155C1CO1.193 cgpGmo-S996
25.8 cgpGmo-S2242
26.7 cgpGmo-S258
27.9 cgpGmo-S1659
31.9 cgpGmo-S2288
33.2 cgpGmo-S2186
34.9 cgpGmo-S263 cgpGmo-S1308
36.7 cgpGmo-S1382
38.3 cgpGmo-S1957 cgpGmo-S822a
42.3 cgpGmo-S1657
43.2 cgpGmo-S962b
48.4 cgpGmo-S1310
61.8 cgpGmo-S28 cgpGmo-S805
68.5 cgpGmo-S1804

CGPIA23

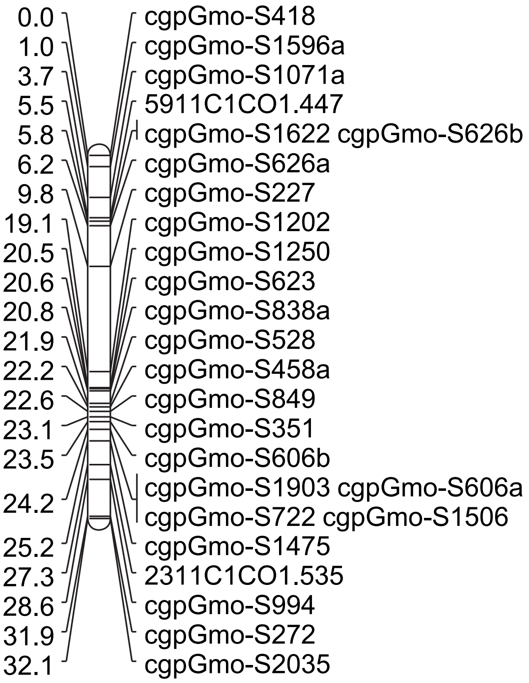
0.0 cgpGmo-S418
1.0 cgpGmo-S1596a
3.7 cgpGmo-S1071a
5.5 5911C1CO1.447
5.8 cgpGmo-S1622 cgpGmo-S626b
6.2 cgpGmo-S626a
9.8 cgpGmo-S227
19.1 cgpGmo-S1202
20.5 cgpGmo-S1250
20.6 cgpGmo-S623
20.8 cgpGmo-S838a
21.9 cgpGmo-S528
22.2 cgpGmo-S458a
22.6 cgpGmo-S849
23.1 cgpGmo-S351
23.5 cgpGmo-S606b
24.2 cgpGmo-S1903 cgpGmo-S606a
cgpGmo-S722 cgpGmo-S1506
25.2 cgpGmo-S1475
27.3 2311C1CO1.535
28.6 cgpGmo-S994
31.9 cgpGmo-S272
32.1 cgpGmo-S2035
