# Supplementary material for: Genomic islands of divergence and their consequences for the resolution of spatial structure in an exploited marine fish
Source: Evol Appl. 2013 Jan 21;6(3):450–61. doi: 10.1111/eva.12026 (PMC3673473; doi:10.1111/eva.12026)
Supplement: Data S3 — LGMAP2. [file eva0006-0450-sd3.pdf]

LGMAP 2 - Supplemental Information

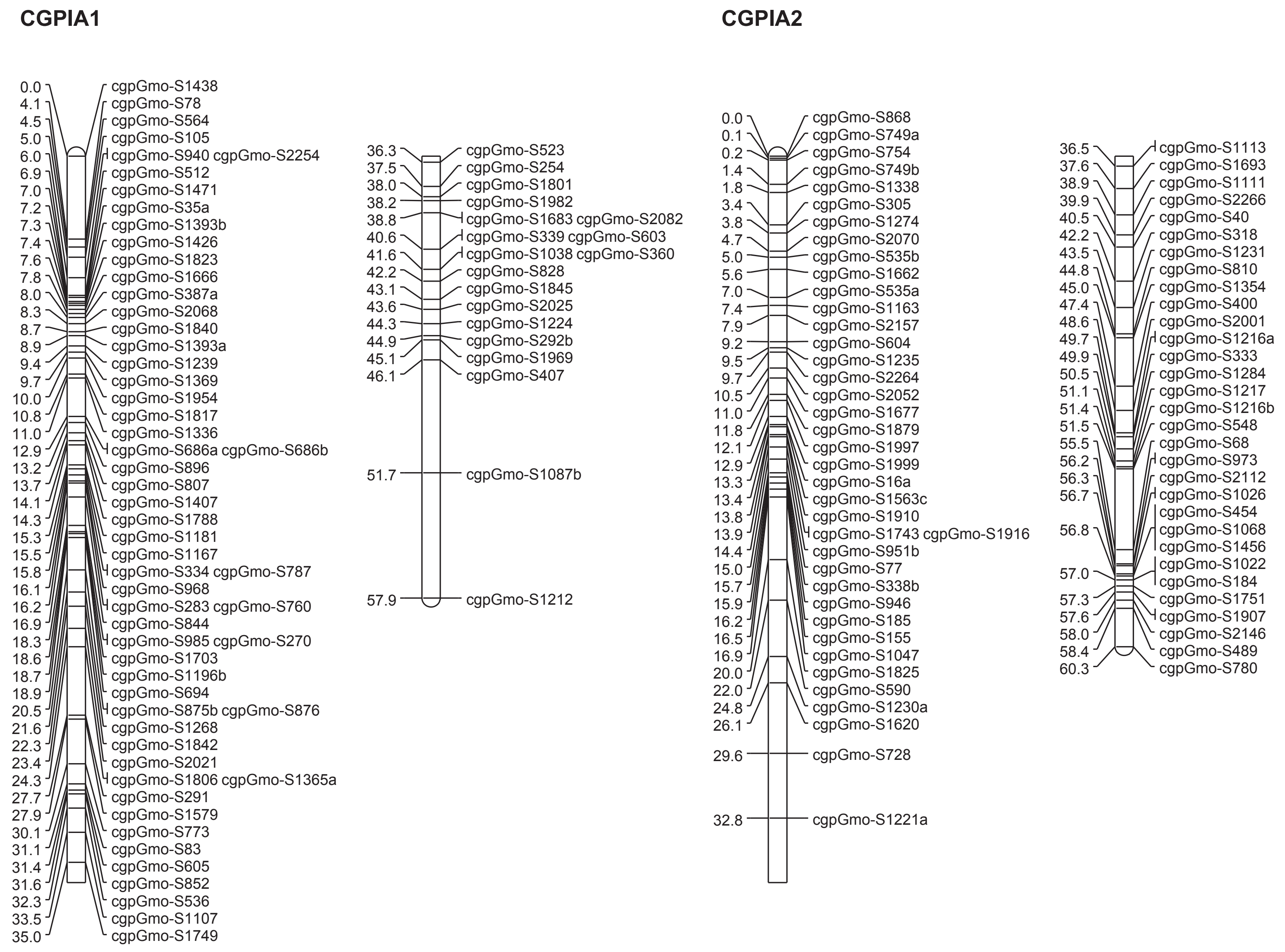

LGMAP 2

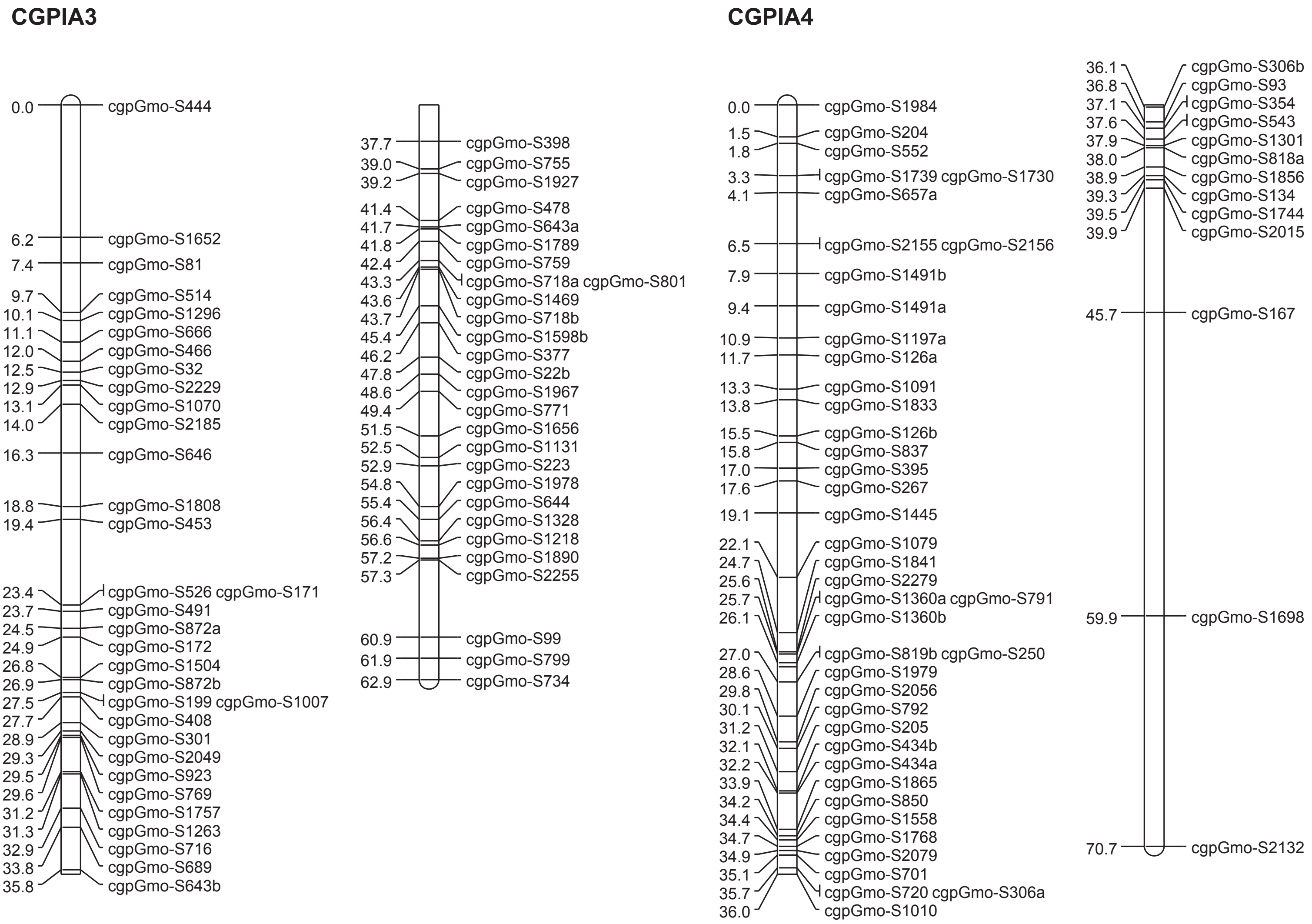
CGPIA3
0.0 cgpGmo-S444
6.2 cgpGmo-S1652
7.4 cgpGmo-S81
9.7 cgpGmo-S514
10.1 cgpGmo-S1296
11.1 cgpGmo-S666
12.0 cgpGmo-S466
12.5 cgpGmo-S32
12.9 cgpGmo-S2229
13.1 cgpGmo-S1070
14.0 cgpGmo-S2185
16.3 cgpGmo-S646
18.8 cgpGmo-S1808
19.4 cgpGmo-S453
23.4 cgpGmo-S526 cgpGmo-S171
23.7 cgpGmo-S491
24.5 cgpGmo-S872a
24.9 cgpGmo-S172
26.8 cgpGmo-S1504
26.9 cgpGmo-S872b
27.5 cgpGmo-S199 cgpGmo-S1007
27.7 cgpGmo-S408
28.9 cgpGmo-S301
29.3 cgpGmo-S2049
29.5 cgpGmo-S923
29.6 cgpGmo-S769
31.2 cgpGmo-S1757
31.3 cgpGmo-S1263
32.9 cgpGmo-S716
33.8 cgpGmo-S689
35.8 cgpGmo-S643b
37.7 cgpGmo-S398
39.0 cgpGmo-S755
39.2 cgpGmo-S1927
41.4 cgpGmo-S478
41.7 cgpGmo-S643a
41.8 cgpGmo-S1789
42.4 cgpGmo-S759
43.3 cgpGmo-S718a cgpGmo-S801
43.6 cgpGmo-S1469
43.7 cgpGmo-S718b
45.4 cgpGmo-S1598b
46.2 cgpGmo-S377
47.8 cgpGmo-S22b
48.6 cgpGmo-S1967
49.4 cgpGmo-S771
51.5 cgpGmo-S1656
52.5 cgpGmo-S1131
52.9 cgpGmo-S223
54.8 cgpGmo-S1978
55.4 cgpGmo-S644
56.4 cgpGmo-S1328
56.6 cgpGmo-S1218
57.2 cgpGmo-S1890
57.3 cgpGmo-S2255
60.9 cgpGmo-S99
61.9 cgpGmo-S799
62.9 cgpGmo-S734
CGPIA4
0.0 cgpGmo-S1984
1.5 cgpGmo-S204
1.8 cgpGmo-S552
3.3 cgpGmo-S1739 cgpGmo-S1730
4.1 cgpGmo-S657a
6.5 cgpGmo-S2155 cgpGmo-S2156
7.9 cgpGmo-S1491b
9.4 cgpGmo-S1491a
10.9 cgpGmo-S1197a
11.7 cgpGmo-S126a
13.3 cgpGmo-S1091
13.8 cgpGmo-S1833
15.5 cgpGmo-S126b
15.8 cgpGmo-S837
17.0 cgpGmo-S395
17.6 cgpGmo-S267
19.1 cgpGmo-S1445
22.1 cgpGmo-S1079
24.7 cgpGmo-S1841
25.6 cgpGmo-S2279
25.7 cgpGmo-S1360a cgpGmo-S791
26.1 cgpGmo-S1360b
27.0 cgpGmo-S819b cgpGmo-S250
28.6 cgpGmo-S1979
29.8 cgpGmo-S2056
30.1 cgpGmo-S792
31.2 cgpGmo-S205
32.1 cgpGmo-S434b
32.2 cgpGmo-S434a
33.9 cgpGmo-S1865
34.2 cgpGmo-S850
34.4 cgpGmo-S1558
34.7 cgpGmo-S1768
34.9 cgpGmo-S2079
35.1 cgpGmo-S701
35.7 cgpGmo-S720 cgpGmo-S306a
36.0 cgpGmo-S1010
36.1 cgpGmo-S306b
36.8 cgpGmo-S93
37.1 cgpGmo-S354
37.6 cgpGmo-S543
37.9 cgpGmo-S1301
38.0 cgpGmo-S818a
38.9 cgpGmo-S1856
39.3 cgpGmo-S134
39.5 cgpGmo-S1744
39.9 cgpGmo-S2015
45.7 cgpGmo-S167
59.9 cgpGmo-S1698
70.7 cgpGmo-S2132

LGMAP 2

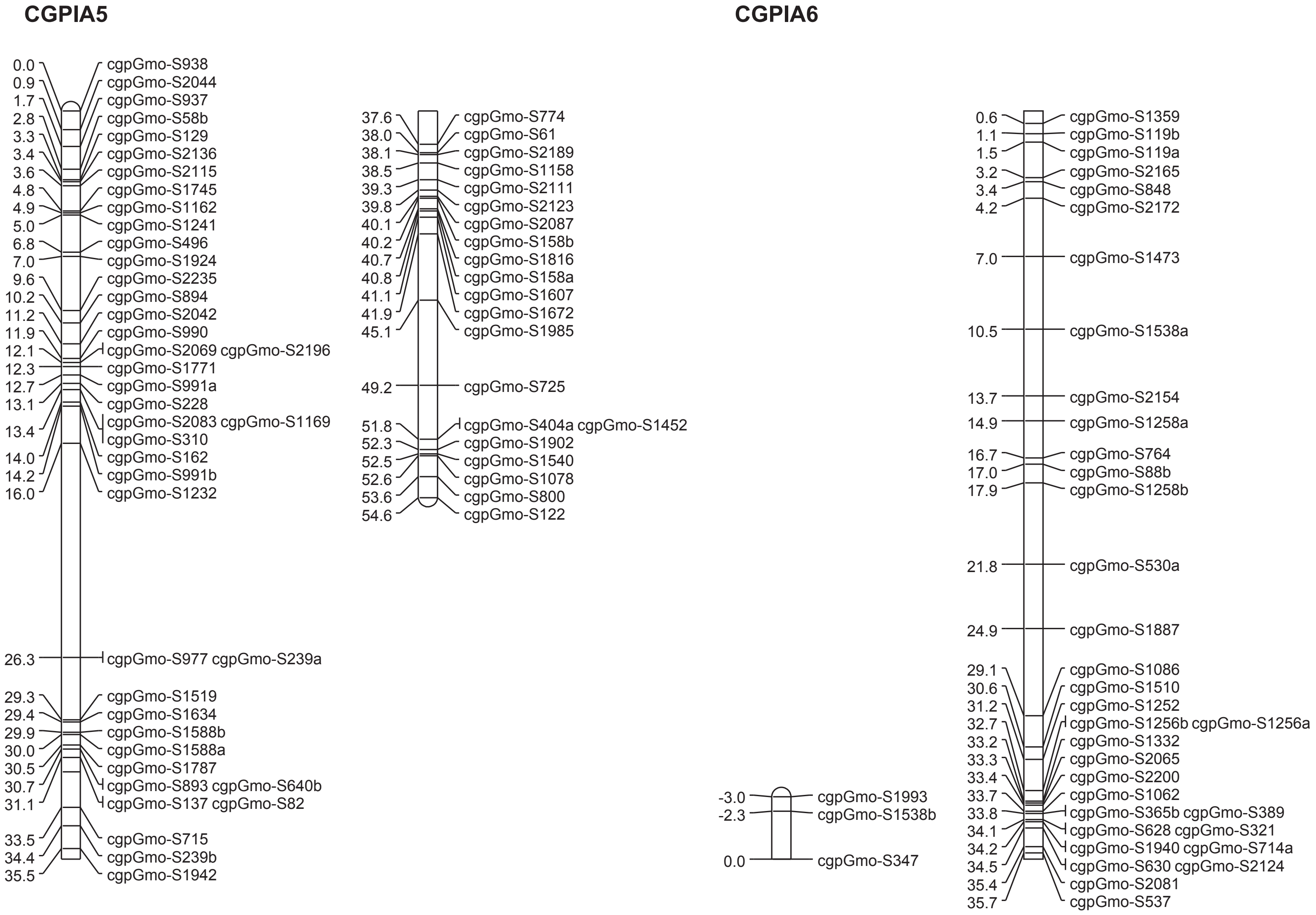
CGPIA5
0.0 cgpGmo-S938
0.9 cgpGmo-S2044
1.7 cgpGmo-S937
2.8 cgpGmo-S58b
3.3 cgpGmo-S129
3.4 cgpGmo-S2136
3.6 cgpGmo-S2115
4.8 cgpGmo-S1745
4.9 cgpGmo-S1162
5.0 cgpGmo-S1241
6.8 cgpGmo-S496
7.0 cgpGmo-S1924
9.6 cgpGmo-S2235
10.2 cgpGmo-S894
11.2 cgpGmo-S2042
11.9 cgpGmo-S990
12.1 cgpGmo-S2069 cgpGmo-S2196
12.3 cgpGmo-S1771
12.7 cgpGmo-S991a
13.1 cgpGmo-S228
13.4 cgpGmo-S2083 cgpGmo-S1169
cgpGmo-S310
14.0 cgpGmo-S162
14.2 cgpGmo-S991b
16.0 cgpGmo-S1232
26.3 cgpGmo-S977 cgpGmo-S239a
29.3 cgpGmo-S1519
29.4 cgpGmo-S1634
29.9 cgpGmo-S1588b
30.0 cgpGmo-S1588a
30.5 cgpGmo-S1787
30.7 cgpGmo-S893 cgpGmo-S640b
31.1 cgpGmo-S137 cgpGmo-S82
33.5 cgpGmo-S715
34.4 cgpGmo-S239b
35.5 cgpGmo-S1942
37.6 cgpGmo-S774
38.0 cgpGmo-S61
38.1 cgpGmo-S2189
38.5 cgpGmo-S1158
39.3 cgpGmo-S2111
39.8 cgpGmo-S2123
40.1 cgpGmo-S2087
40.2 cgpGmo-S158b
40.7 cgpGmo-S1816
40.8 cgpGmo-S158a
41.1 cgpGmo-S1607
41.9 cgpGmo-S1672
45.1 cgpGmo-S1985
49.2 cgpGmo-S725
51.8 cgpGmo-S404a cgpGmo-S1452
52.3 cgpGmo-S1902
52.5 cgpGmo-S1540
52.6 cgpGmo-S1078
53.6 cgpGmo-S800
54.6 cgpGmo-S122
CGPIA6
-3.0 cgpGmo-S1993
-2.3 cgpGmo-S1538b
0.0 cgpGmo-S347
0.6 cgpGmo-S1359
1.1 cgpGmo-S119b
1.5 cgpGmo-S119a
3.2 cgpGmo-S2165
3.4 cgpGmo-S848
4.2 cgpGmo-S2172
7.0 cgpGmo-S1473
10.5 cgpGmo-S1538a
13.7 cgpGmo-S2154
14.9 cgpGmo-S1258a
16.7 cgpGmo-S764
17.0 cgpGmo-S88b
17.9 cgpGmo-S1258b
21.8 cgpGmo-S530a
24.9 cgpGmo-S1887
29.1 cgpGmo-S1086
30.6 cgpGmo-S1510
31.2 cgpGmo-S1252
32.7 cgpGmo-S1256b cgpGmo-S1256a
33.2 cgpGmo-S1332
33.3 cgpGmo-S2065
33.4 cgpGmo-S2200
33.7 cgpGmo-S1062
33.8 cgpGmo-S365b cgpGmo-S389
34.1 cgpGmo-S628 cgpGmo-S321
34.2 cgpGmo-S1940 cgpGmo-S714a
34.5 cgpGmo-S630 cgpGmo-S2124
35.4 cgpGmo-S2081
35.7 cgpGmo-S537

LGMAP 2

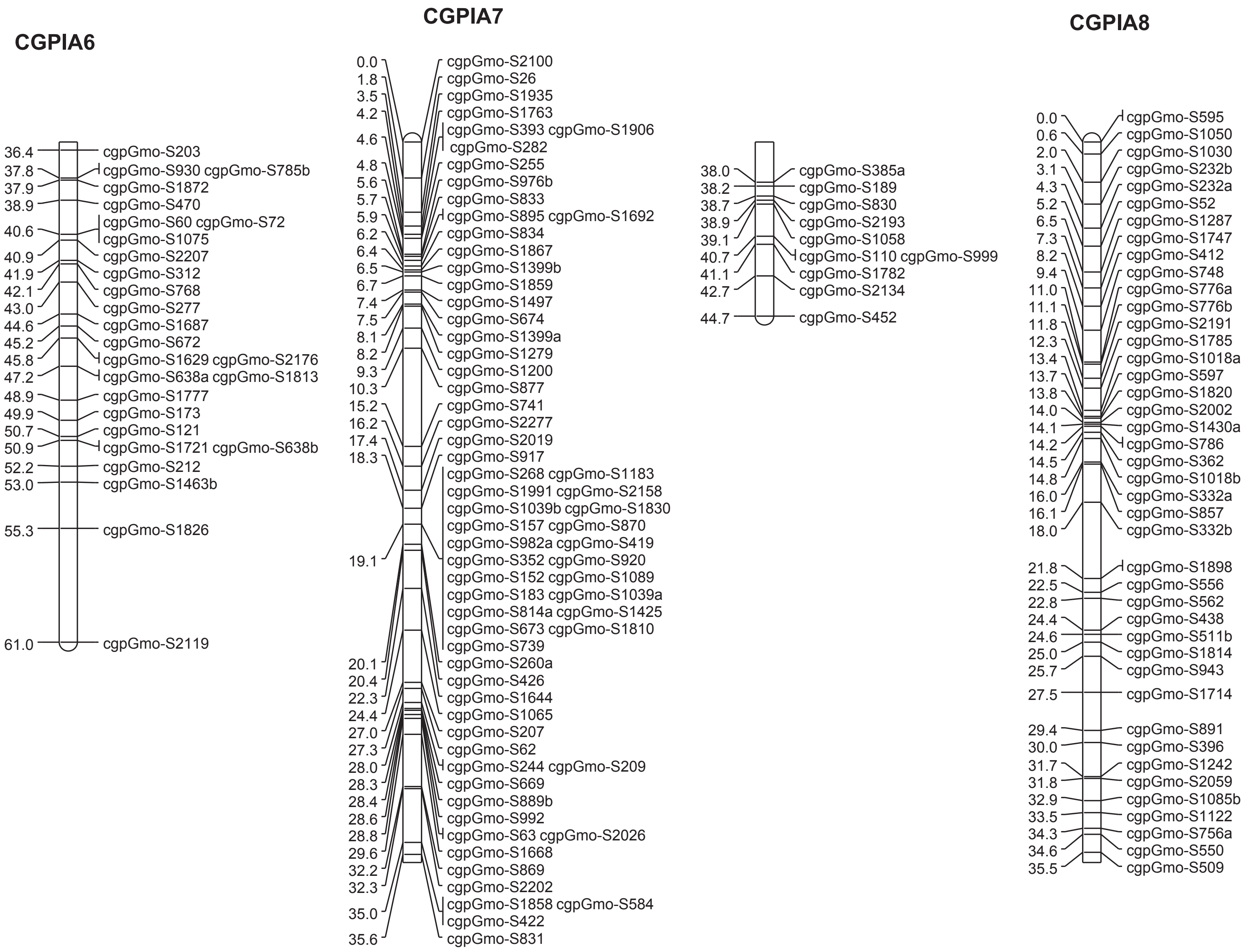
CGPIA6
36.4 cgpGmo-S203
37.8 cgpGmo-S930 cgpGmo-S785b
37.9 cgpGmo-S1872
38.9 cgpGmo-S470
40.6 cgpGmo-S60 cgpGmo-S72 cgpGmo-S1075
40.9 cgpGmo-S2207
41.9 cgpGmo-S312
42.1 cgpGmo-S768
43.0 cgpGmo-S277
44.6 cgpGmo-S1687
45.2 cgpGmo-S672
45.8 cgpGmo-S1629 cgpGmo-S2176
47.2 cgpGmo-S638a cgpGmo-S1813
48.9 cgpGmo-S1777
49.9 cgpGmo-S173
50.7 cgpGmo-S121
50.9 cgpGmo-S1721 cgpGmo-S638b
52.2 cgpGmo-S212
53.0 cgpGmo-S1463b
55.3 cgpGmo-S1826
61.0 cgpGmo-S2119
CGPIA7
0.0 cgpGmo-S2100
1.8 cgpGmo-S26
3.5 cgpGmo-S1935
4.2 cgpGmo-S1763
4.6 cgpGmo-S393 cgpGmo-S1906 cgpGmo-S282
4.8 cgpGmo-S255
5.6 cgpGmo-S976b
5.7 cgpGmo-S833
5.9 cgpGmo-S895 cgpGmo-S1692
6.2 cgpGmo-S834
6.4 cgpGmo-S1867
6.5 cgpGmo-S1399b
6.7 cgpGmo-S1859
7.4 cgpGmo-S1497
7.5 cgpGmo-S674
8.1 cgpGmo-S1399a
8.2 cgpGmo-S1279
9.3 cgpGmo-S1200
10.3 cgpGmo-S877
15.2 cgpGmo-S741
16.2 cgpGmo-S2277
17.4 cgpGmo-S2019
18.3 cgpGmo-S917
19.1 cgpGmo-S268 cgpGmo-S1183 cgpGmo-S1991 cgpGmo-S2158 cgpGmo-S1039b cgpGmo-S1830 cgpGmo-S157 cgpGmo-S870 cgpGmo-S982a cgpGmo-S419 cgpGmo-S352 cgpGmo-S920 cgpGmo-S152 cgpGmo-S1089 cgpGmo-S183 cgpGmo-S1039a cgpGmo-S814a cgpGmo-S1425 cgpGmo-S673 cgpGmo-S1810 cgpGmo-S739
20.1 cgpGmo-S260a
20.4 cgpGmo-S426
22.3 cgpGmo-S1644
24.4 cgpGmo-S1065
27.0 cgpGmo-S207
27.3 cgpGmo-S62
28.0 cgpGmo-S244 cgpGmo-S209
28.3 cgpGmo-S669
28.4 cgpGmo-S889b
28.6 cgpGmo-S992
28.8 cgpGmo-S63 cgpGmo-S2026
29.6 cgpGmo-S1668
32.2 cgpGmo-S869
32.3 cgpGmo-S2202
35.0 cgpGmo-S1858 cgpGmo-S584 cgpGmo-S422
35.6 cgpGmo-S831
38.0 cgpGmo-S385a
38.2 cgpGmo-S189
38.7 cgpGmo-S830
38.9 cgpGmo-S2193
39.1 cgpGmo-S1058
40.7 cgpGmo-S110 cgpGmo-S999
41.1 cgpGmo-S1782
42.7 cgpGmo-S2134
44.7 cgpGmo-S452
CGPIA8
0.0 cgpGmo-S595
0.6 cgpGmo-S1050
2.0 cgpGmo-S1030
3.1 cgpGmo-S232b
4.3 cgpGmo-S232a
5.2 cgpGmo-S52
6.5 cgpGmo-S1287
7.3 cgpGmo-S1747
8.2 cgpGmo-S412
9.4 cgpGmo-S748
11.0 cgpGmo-S776a
11.1 cgpGmo-S776b
11.8 cgpGmo-S2191
12.3 cgpGmo-S1785
13.4 cgpGmo-S1018a
13.7 cgpGmo-S597
13.8 cgpGmo-S1820
14.0 cgpGmo-S2002
14.1 cgpGmo-S1430a
14.2 cgpGmo-S786
14.5 cgpGmo-S362
14.8 cgpGmo-S1018b
16.0 cgpGmo-S332a
16.1 cgpGmo-S857
18.0 cgpGmo-S332b
21.8 cgpGmo-S1898
22.5 cgpGmo-S556
22.8 cgpGmo-S562
24.4 cgpGmo-S438
24.6 cgpGmo-S511b
25.0 cgpGmo-S1814
25.7 cgpGmo-S943
27.5 cgpGmo-S1714
29.4 cgpGmo-S891
30.0 cgpGmo-S396
31.7 cgpGmo-S1242
31.8 cgpGmo-S2059
32.9 cgpGmo-S1085b
33.5 cgpGmo-S1122
34.3 cgpGmo-S756a
34.6 cgpGmo-S550
35.5 cgpGmo-S509

LGMAP 2

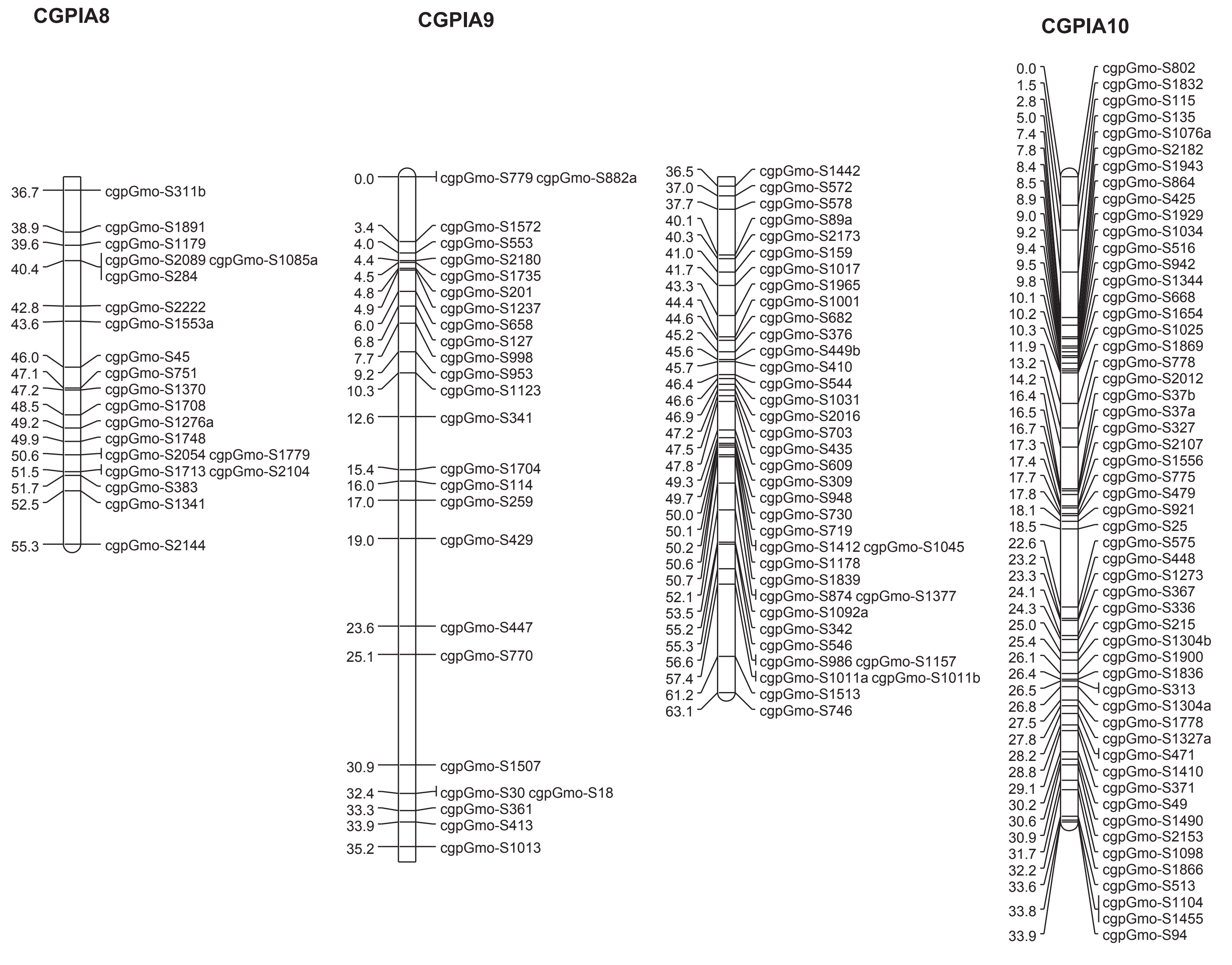

CGPIA8
36.7 cgpGmo-S311b
38.9 cgpGmo-S1891
39.6 cgpGmo-S1179
40.4 cgpGmo-S2089 cgpGmo-S1085a
cgpGmo-S284
42.8 cgpGmo-S2222
43.6 cgpGmo-S1553a
46.0 cgpGmo-S45
47.1 cgpGmo-S751
47.2 cgpGmo-S1370
48.5 cgpGmo-S1708
49.2 cgpGmo-S1276a
49.9 cgpGmo-S1748
50.6 cgpGmo-S2054 cgpGmo-S1779
51.5 cgpGmo-S1713 cgpGmo-S2104
51.7 cgpGmo-S383
52.5 cgpGmo-S1341
55.3 cgpGmo-S2144
CGPIA9
0.0 cgpGmo-S779 cgpGmo-S882a
3.4 cgpGmo-S1572
4.0 cgpGmo-S553
4.4 cgpGmo-S2180
4.5 cgpGmo-S1735
4.8 cgpGmo-S201
4.9 cgpGmo-S1237
6.0 cgpGmo-S658
6.8 cgpGmo-S127
7.7 cgpGmo-S998
9.2 cgpGmo-S953
10.3 cgpGmo-S1123
12.6 cgpGmo-S341
15.4 cgpGmo-S1704
16.0 cgpGmo-S114
17.0 cgpGmo-S259
19.0 cgpGmo-S429
23.6 cgpGmo-S447
25.1 cgpGmo-S770
30.9 cgpGmo-S1507
32.4 cgpGmo-S30 cgpGmo-S18
33.3 cgpGmo-S361
33.9 cgpGmo-S413
35.2 cgpGmo-S1013
36.5 cgpGmo-S1442
37.0 cgpGmo-S572
37.7 cgpGmo-S578
40.1 cgpGmo-S89a
40.3 cgpGmo-S2173
41.0 cgpGmo-S159
41.7 cgpGmo-S1017
43.3 cgpGmo-S1965
44.4 cgpGmo-S1001
44.6 cgpGmo-S682
45.2 cgpGmo-S376
45.6 cgpGmo-S449b
45.7 cgpGmo-S410
46.4 cgpGmo-S544
46.6 cgpGmo-S1031
46.9 cgpGmo-S2016
47.2 cgpGmo-S703
47.5 cgpGmo-S435
47.8 cgpGmo-S609
49.3 cgpGmo-S309
49.7 cgpGmo-S948
50.0 cgpGmo-S730
50.1 cgpGmo-S719
50.2 cgpGmo-S1412 cgpGmo-S1045
50.6 cgpGmo-S1178
50.7 cgpGmo-S1839
52.1 cgpGmo-S874 cgpGmo-S1377
53.5 cgpGmo-S1092a
55.2 cgpGmo-S342
55.3 cgpGmo-S546
56.6 cgpGmo-S986 cgpGmo-S1157
57.4 cgpGmo-S1011a cgpGmo-S1011b
61.2 cgpGmo-S1513
63.1 cgpGmo-S746
CGPIA10
0.0 cgpGmo-S802
1.5 cgpGmo-S1832
2.8 cgpGmo-S115
5.0 cgpGmo-S135
7.4 cgpGmo-S1076a
7.8 cgpGmo-S2182
8.4 cgpGmo-S1943
8.5 cgpGmo-S864
8.9 cgpGmo-S425
9.0 cgpGmo-S1929
9.2 cgpGmo-S1034
9.4 cgpGmo-S516
9.5 cgpGmo-S942
9.8 cgpGmo-S1344
10.1 cgpGmo-S668
10.2 cgpGmo-S1654
10.3 cgpGmo-S1025
11.9 cgpGmo-S1869
13.2 cgpGmo-S778
14.2 cgpGmo-S2012
16.4 cgpGmo-S37b
16.5 cgpGmo-S37a
16.7 cgpGmo-S327
17.3 cgpGmo-S2107
17.4 cgpGmo-S1556
17.7 cgpGmo-S775
17.8 cgpGmo-S479
18.1 cgpGmo-S921
18.5 cgpGmo-S25
22.6 cgpGmo-S575
23.2 cgpGmo-S448
23.3 cgpGmo-S1273
24.1 cgpGmo-S367
24.3 cgpGmo-S336
25.0 cgpGmo-S215
25.4 cgpGmo-S1304b
26.1 cgpGmo-S1900
26.4 cgpGmo-S1836
26.5 cgpGmo-S313
26.8 cgpGmo-S1304a
27.5 cgpGmo-S1778
27.8 cgpGmo-S1327a
28.2 cgpGmo-S471
28.8 cgpGmo-S1410
29.1 cgpGmo-S371
30.2 cgpGmo-S49
30.6 cgpGmo-S1490
30.9 cgpGmo-S2153
31.7 cgpGmo-S1098
32.2 cgpGmo-S1866
33.6 cgpGmo-S513
33.8 cgpGmo-S1104
cgpGmo-S1455
33.9 cgpGmo-S94

LGMAP 2

CGPIA11

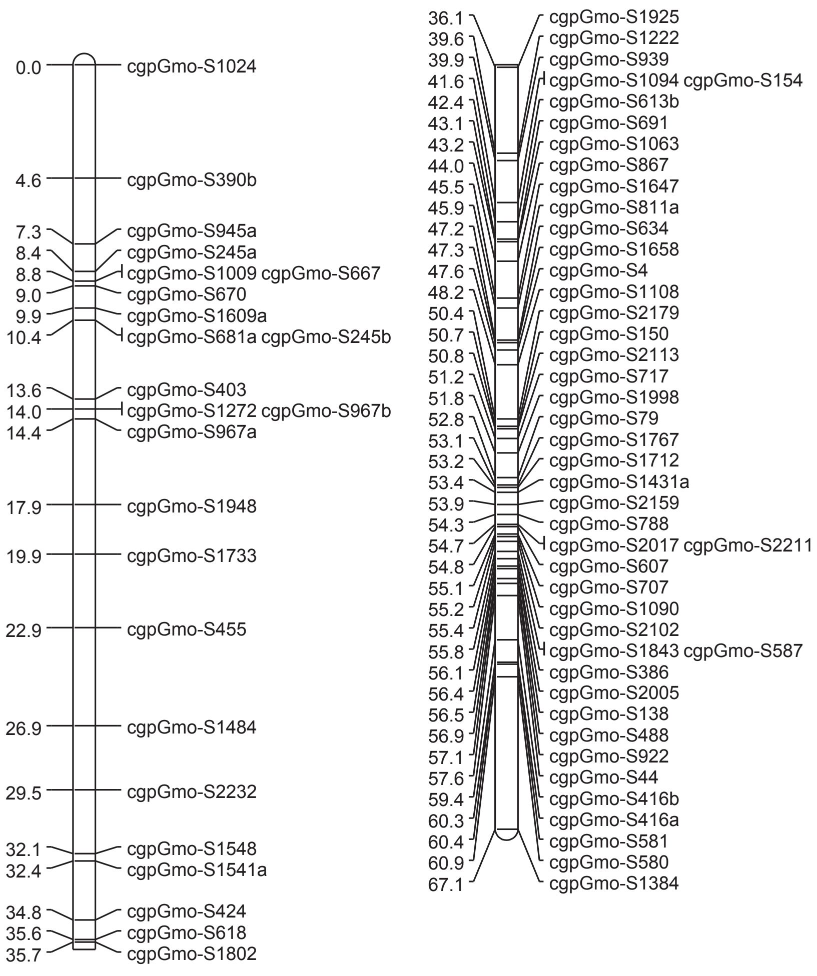

CGPIA12

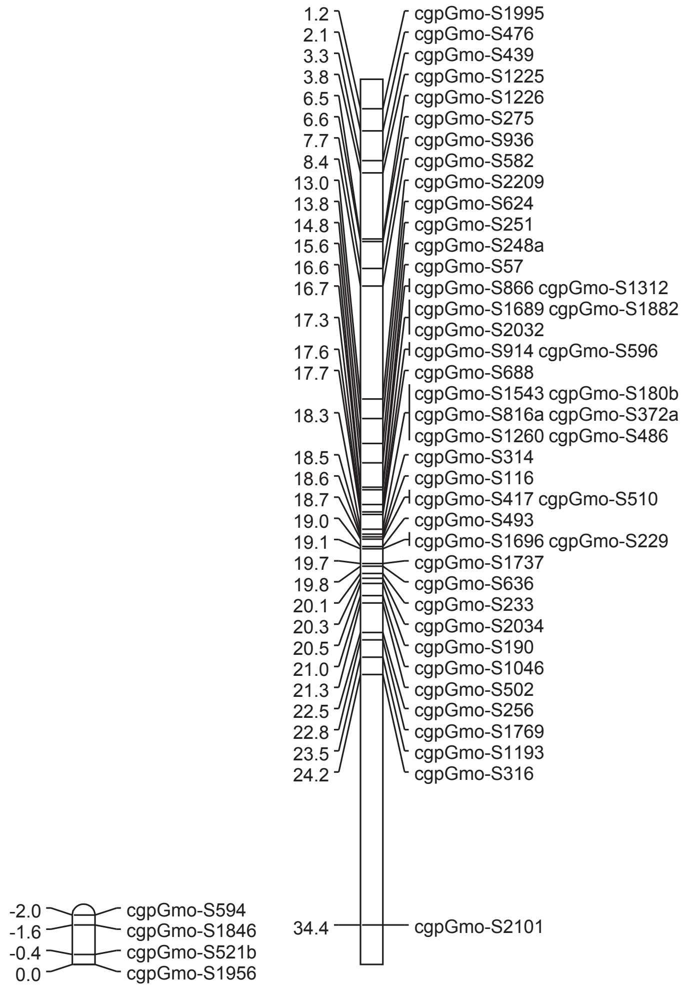

LGMAP 2

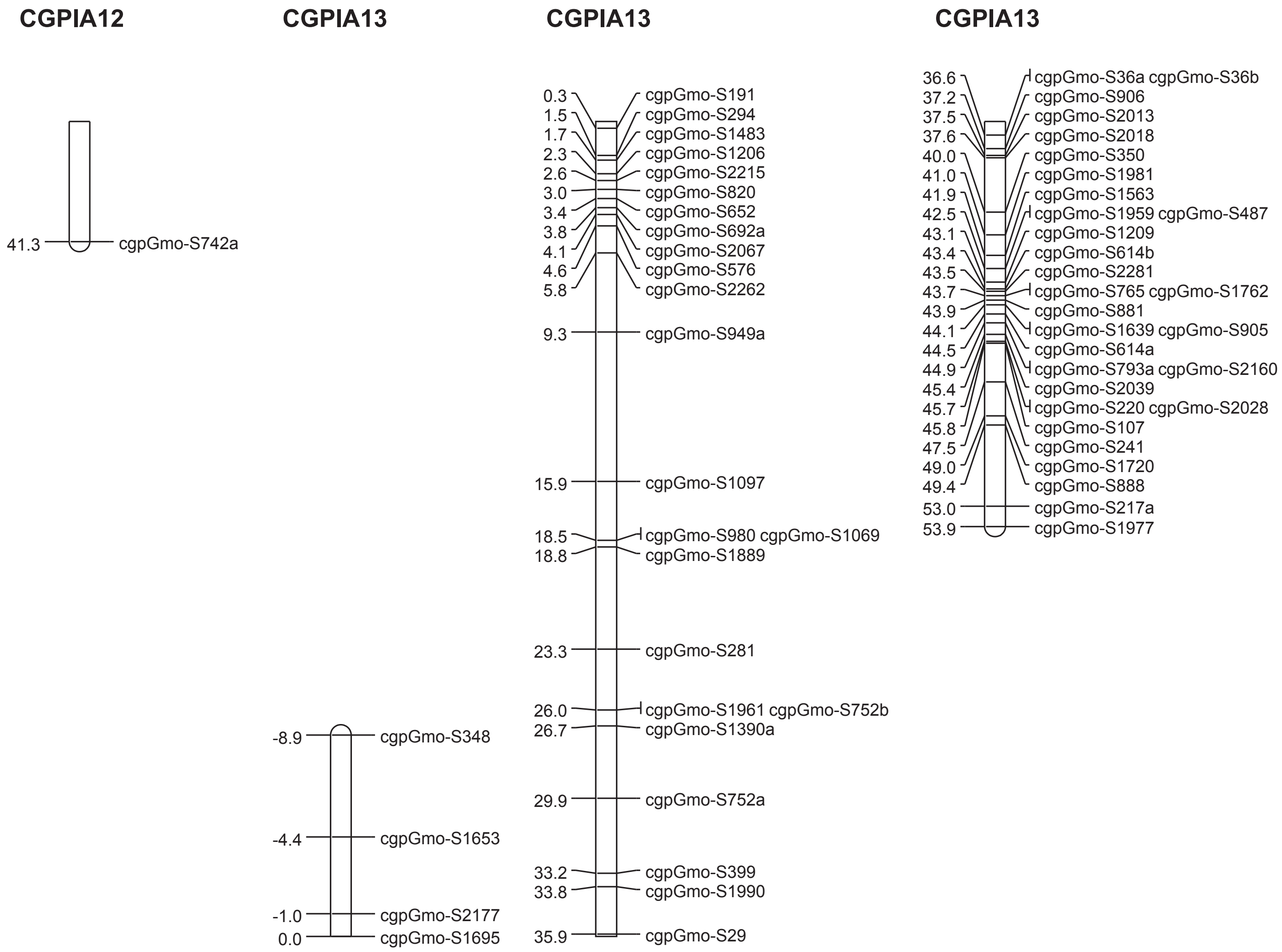

CGPIA12
41.3 cgpGmo-S742a
CGPIA13
-8.9 cgpGmo-S348
-4.4 cgpGmo-S1653
-1.0 cgpGmo-S2177
0.0 cgpGmo-S1695
CGPIA13
0.3 cgpGmo-S191
1.5 cgpGmo-S294
1.7 cgpGmo-S1483
2.3 cgpGmo-S1206
2.6 cgpGmo-S2215
3.0 cgpGmo-S820
3.4 cgpGmo-S652
3.8 cgpGmo-S692a
4.1 cgpGmo-S2067
4.6 cgpGmo-S576
5.8 cgpGmo-S2262
9.3 cgpGmo-S949a
15.9 cgpGmo-S1097
18.5 cgpGmo-S980 cgpGmo-S1069
18.8 cgpGmo-S1889
23.3 cgpGmo-S281
26.0 cgpGmo-S1961 cgpGmo-S752b
26.7 cgpGmo-S1390a
29.9 cgpGmo-S752a
33.2 cgpGmo-S399
33.8 cgpGmo-S1990
35.9 cgpGmo-S29
CGPIA13
36.6 cgpGmo-S36a cgpGmo-S36b
37.2 cgpGmo-S906
37.5 cgpGmo-S2013
37.6 cgpGmo-S2018
40.0 cgpGmo-S350
41.0 cgpGmo-S1981
41.9 cgpGmo-S1563
42.5 cgpGmo-S1959 cgpGmo-S487
43.1 cgpGmo-S1209
43.4 cgpGmo-S614b
43.5 cgpGmo-S2281
43.7 cgpGmo-S765 cgpGmo-S1762
43.9 cgpGmo-S881
44.1 cgpGmo-S1639 cgpGmo-S905
44.5 cgpGmo-S614a
44.9 cgpGmo-S793a cgpGmo-S2160
45.4 cgpGmo-S2039
45.7 cgpGmo-S220 cgpGmo-S2028
45.8 cgpGmo-S107
47.5 cgpGmo-S241
49.0 cgpGmo-S1720
49.4 cgpGmo-S888
53.0 cgpGmo-S217a
53.9 cgpGmo-S1977

LGMAP 2

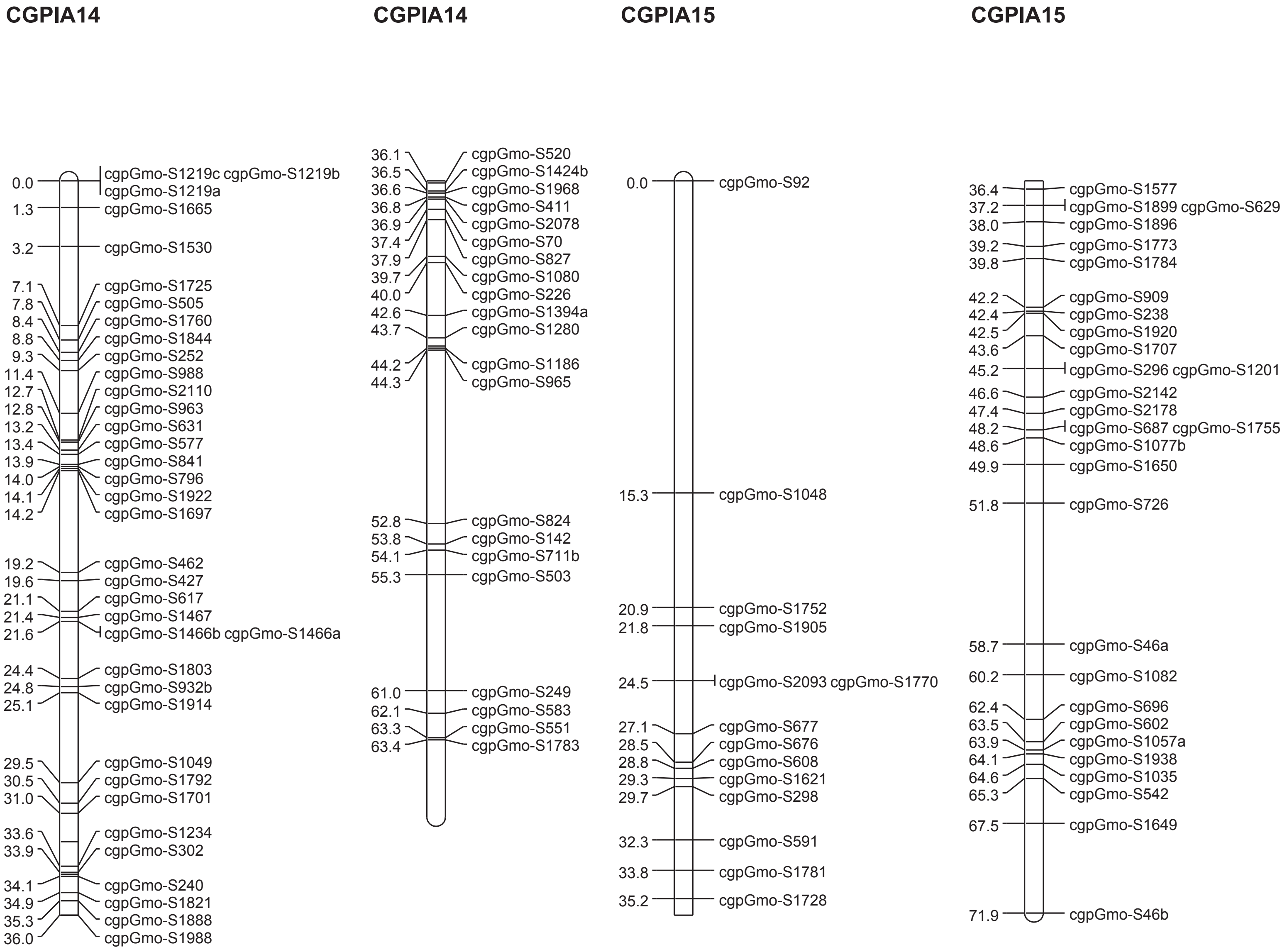
CGPIA14
0.0 cgpGmo-S1219c cgpGmo-S1219b
cgpGmo-S1219a
1.3 cgpGmo-S1665
3.2 cgpGmo-S1530
7.1 cgpGmo-S1725
7.8 cgpGmo-S505
8.4 cgpGmo-S1760
8.8 cgpGmo-S1844
9.3 cgpGmo-S252
11.4 cgpGmo-S988
12.7 cgpGmo-S2110
12.8 cgpGmo-S963
13.2 cgpGmo-S631
13.4 cgpGmo-S577
13.9 cgpGmo-S841
14.0 cgpGmo-S796
14.1 cgpGmo-S1922
14.2 cgpGmo-S1697
19.2 cgpGmo-S462
19.6 cgpGmo-S427
21.1 cgpGmo-S617
21.4 cgpGmo-S1467
21.6 cgpGmo-S1466b cgpGmo-S1466a
24.4 cgpGmo-S1803
24.8 cgpGmo-S932b
25.1 cgpGmo-S1914
29.5 cgpGmo-S1049
30.5 cgpGmo-S1792
31.0 cgpGmo-S1701
33.6 cgpGmo-S1234
33.9 cgpGmo-S302
34.1 cgpGmo-S240
34.9 cgpGmo-S1821
35.3 cgpGmo-S1888
36.0 cgpGmo-S1988
CGPIA14
36.1 cgpGmo-S520
36.5 cgpGmo-S1424b
36.6 cgpGmo-S1968
36.8 cgpGmo-S411
36.9 cgpGmo-S2078
37.4 cgpGmo-S70
37.9 cgpGmo-S827
39.7 cgpGmo-S1080
40.0 cgpGmo-S226
42.6 cgpGmo-S1394a
43.7 cgpGmo-S1280
44.2 cgpGmo-S1186
44.3 cgpGmo-S965
52.8 cgpGmo-S824
53.8 cgpGmo-S142
54.1 cgpGmo-S711b
55.3 cgpGmo-S503
61.0 cgpGmo-S249
62.1 cgpGmo-S583
63.3 cgpGmo-S551
63.4 cgpGmo-S1783
CGPIA15
0.0 cgpGmo-S92
15.3 cgpGmo-S1048
20.9 cgpGmo-S1752
21.8 cgpGmo-S1905
24.5 cgpGmo-S2093 cgpGmo-S1770
27.1 cgpGmo-S677
28.5 cgpGmo-S676
28.8 cgpGmo-S608
29.3 cgpGmo-S1621
29.7 cgpGmo-S298
32.3 cgpGmo-S591
33.8 cgpGmo-S1781
35.2 cgpGmo-S1728
CGPIA15
36.4 cgpGmo-S1577
37.2 cgpGmo-S1899 cgpGmo-S629
38.0 cgpGmo-S1896
39.2 cgpGmo-S1773
39.8 cgpGmo-S1784
42.2 cgpGmo-S909
42.4 cgpGmo-S238
42.5 cgpGmo-S1920
43.6 cgpGmo-S1707
45.2 cgpGmo-S296 cgpGmo-S1201
46.6 cgpGmo-S2142
47.4 cgpGmo-S2178
48.2 cgpGmo-S687 cgpGmo-S1755
48.6 cgpGmo-S1077b
49.9 cgpGmo-S1650
51.8 cgpGmo-S726
58.7 cgpGmo-S46a
60.2 cgpGmo-S1082
62.4 cgpGmo-S696
63.5 cgpGmo-S602
63.9 cgpGmo-S1057a
64.1 cgpGmo-S1938
64.6 cgpGmo-S1035
65.3 cgpGmo-S542
67.5 cgpGmo-S1649
71.9 cgpGmo-S46b

LGMAP 2

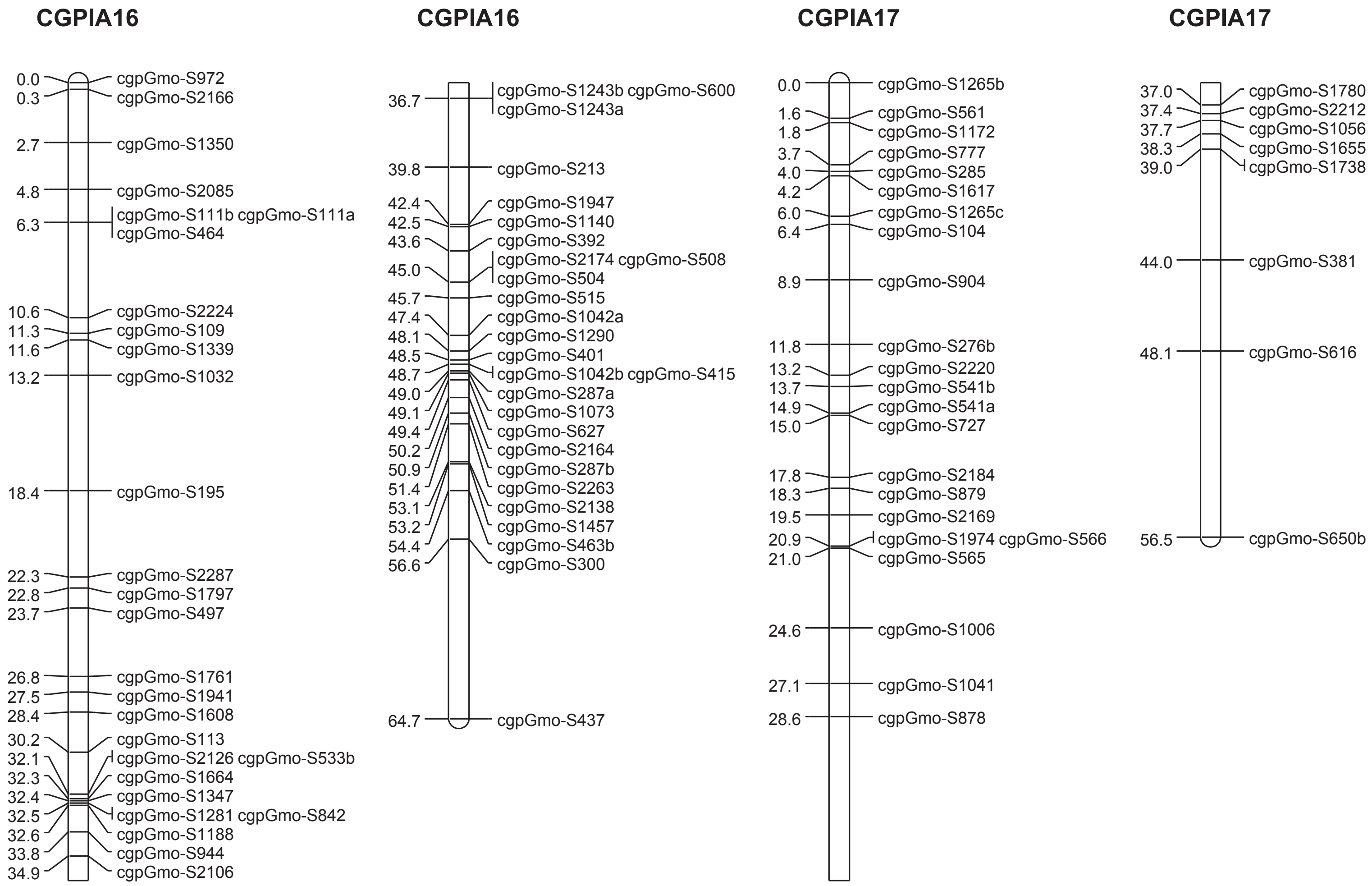
CGPIA16
0.0 cgpGmo-S972
0.3 cgpGmo-S2166
2.7 cgpGmo-S1350
4.8 cgpGmo-S2085
6.3 cgpGmo-S111b cgpGmo-S111a
cgpGmo-S464
10.6 cgpGmo-S2224
11.3 cgpGmo-S109
11.6 cgpGmo-S1339
13.2 cgpGmo-S1032
18.4 cgpGmo-S195
22.3 cgpGmo-S2287
22.8 cgpGmo-S1797
23.7 cgpGmo-S497
26.8 cgpGmo-S1761
27.5 cgpGmo-S1941
28.4 cgpGmo-S1608
30.2 cgpGmo-S113
32.1 cgpGmo-S2126 cgpGmo-S533b
32.3 cgpGmo-S1664
32.4 cgpGmo-S1347
32.5 cgpGmo-S1281 cgpGmo-S842
32.6 cgpGmo-S1188
33.8 cgpGmo-S944
34.9 cgpGmo-S2106
CGPIA16
36.7 cgpGmo-S1243b cgpGmo-S600
cgpGmo-S1243a
39.8 cgpGmo-S213
42.4 cgpGmo-S1947
42.5 cgpGmo-S1140
43.6 cgpGmo-S392
45.0 cgpGmo-S2174 cgpGmo-S508
cgpGmo-S504
45.7 cgpGmo-S515
47.4 cgpGmo-S1042a
48.1 cgpGmo-S1290
48.5 cgpGmo-S401
48.7 cgpGmo-S1042b cgpGmo-S415
49.0 cgpGmo-S287a
49.1 cgpGmo-S1073
49.4 cgpGmo-S627
50.2 cgpGmo-S2164
50.9 cgpGmo-S287b
51.4 cgpGmo-S2263
53.1 cgpGmo-S2138
53.2 cgpGmo-S1457
54.4 cgpGmo-S463b
56.6 cgpGmo-S300
64.7 cgpGmo-S437
CGPIA17
0.0 cgpGmo-S1265b
1.6 cgpGmo-S561
1.8 cgpGmo-S1172
3.7 cgpGmo-S777
4.0 cgpGmo-S285
4.2 cgpGmo-S1617
6.0 cgpGmo-S1265c
6.4 cgpGmo-S104
8.9 cgpGmo-S904
11.8 cgpGmo-S276b
13.2 cgpGmo-S2220
13.7 cgpGmo-S541b
14.9 cgpGmo-S541a
15.0 cgpGmo-S727
17.8 cgpGmo-S2184
18.3 cgpGmo-S879
19.5 cgpGmo-S2169
20.9 cgpGmo-S1974 cgpGmo-S566
21.0 cgpGmo-S565
24.6 cgpGmo-S1006
27.1 cgpGmo-S1041
28.6 cgpGmo-S878
CGPIA17
37.0 cgpGmo-S1780
37.4 cgpGmo-S2212
37.7 cgpGmo-S1056
38.3 cgpGmo-S1655
39.0 cgpGmo-S1738
44.0 cgpGmo-S381
48.1 cgpGmo-S616
56.5 cgpGmo-S650b

LGMAP 2

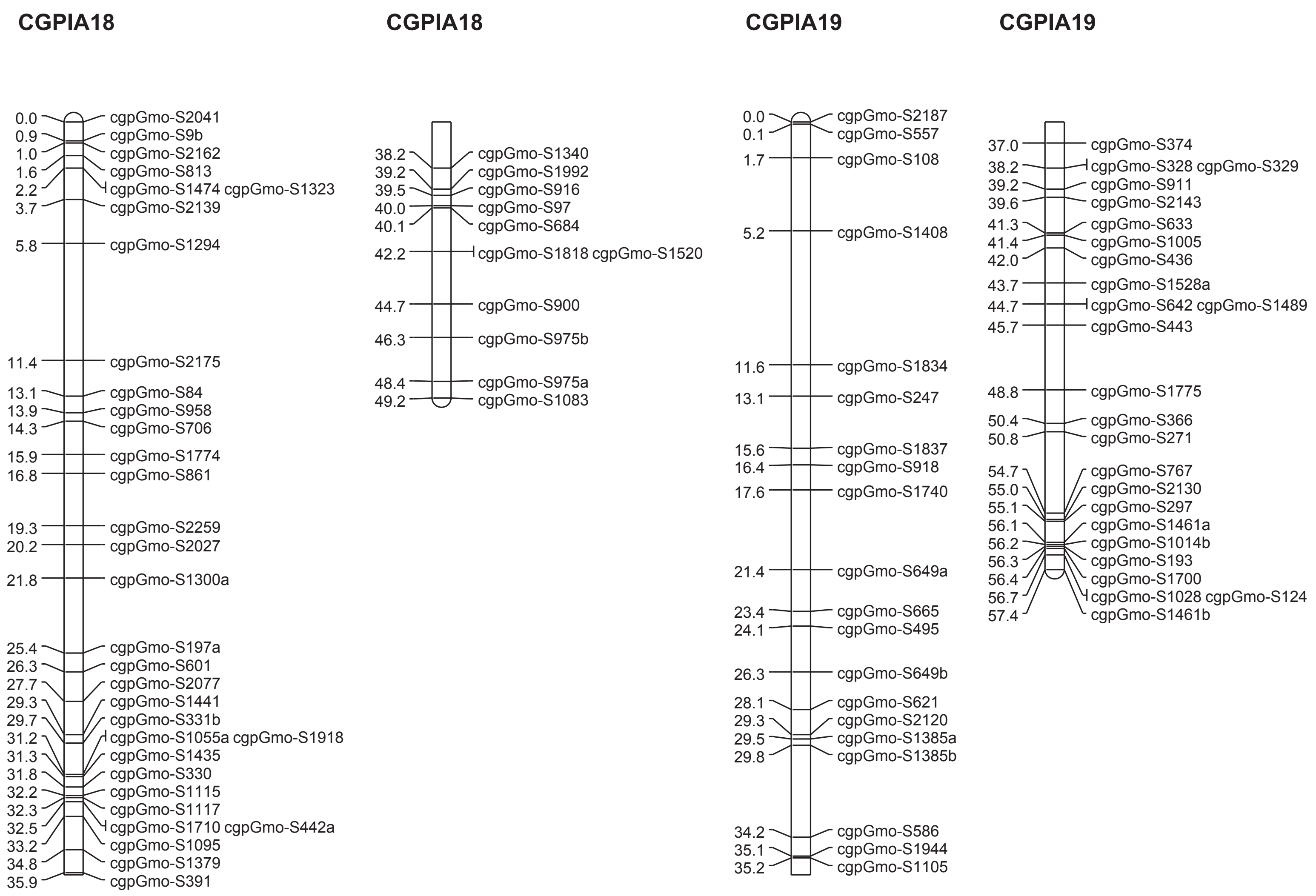
CGPIA18
CGPIA18
CGPIA19
CGPIA19
0.0 cgpGmo-S2041
0.9 cgpGmo-S9b
1.0 cgpGmo-S2162
1.6 cgpGmo-S813
2.2 cgpGmo-S1474 cgpGmo-S1323
3.7 cgpGmo-S2139
5.8 cgpGmo-S1294
11.4 cgpGmo-S2175
13.1 cgpGmo-S84
13.9 cgpGmo-S958
14.3 cgpGmo-S706
15.9 cgpGmo-S1774
16.8 cgpGmo-S861
19.3 cgpGmo-S2259
20.2 cgpGmo-S2027
21.8 cgpGmo-S1300a
25.4 cgpGmo-S197a
26.3 cgpGmo-S601
27.7 cgpGmo-S2077
29.3 cgpGmo-S1441
29.7 cgpGmo-S331b
31.2 cgpGmo-S1055a cgpGmo-S1918
31.3 cgpGmo-S1435
31.8 cgpGmo-S330
32.2 cgpGmo-S1115
32.3 cgpGmo-S1117
32.5 cgpGmo-S1710 cgpGmo-S442a
33.2 cgpGmo-S1095
34.8 cgpGmo-S1379
35.9 cgpGmo-S391
38.2 cgpGmo-S1340
39.2 cgpGmo-S1992
39.5 cgpGmo-S916
40.0 cgpGmo-S97
40.1 cgpGmo-S684
42.2 cgpGmo-S1818 cgpGmo-S1520
44.7 cgpGmo-S900
46.3 cgpGmo-S975b
48.4 cgpGmo-S975a
49.2 cgpGmo-S1083
0.0 cgpGmo-S2187
0.1 cgpGmo-S557
1.7 cgpGmo-S108
5.2 cgpGmo-S1408
11.6 cgpGmo-S1834
13.1 cgpGmo-S247
15.6 cgpGmo-S1837
16.4 cgpGmo-S918
17.6 cgpGmo-S1740
21.4 cgpGmo-S649a
23.4 cgpGmo-S665
24.1 cgpGmo-S495
26.3 cgpGmo-S649b
28.1 cgpGmo-S621
29.3 cgpGmo-S2120
29.5 cgpGmo-S1385a
29.8 cgpGmo-S1385b
34.2 cgpGmo-S586
35.1 cgpGmo-S1944
35.2 cgpGmo-S1105
37.0 cgpGmo-S374
38.2 cgpGmo-S328 cgpGmo-S329
39.2 cgpGmo-S911
39.6 cgpGmo-S2143
41.3 cgpGmo-S633
41.4 cgpGmo-S1005
42.0 cgpGmo-S436
43.7 cgpGmo-S1528a
44.7 cgpGmo-S642 cgpGmo-S1489
45.7 cgpGmo-S443
48.8 cgpGmo-S1775
50.4 cgpGmo-S366
50.8 cgpGmo-S271
54.7 cgpGmo-S767
55.0 cgpGmo-S2130
55.1 cgpGmo-S297
56.1 cgpGmo-S1461a
56.2 cgpGmo-S1014b
56.3 cgpGmo-S193
56.4 cgpGmo-S1700
56.7 cgpGmo-S1028 cgpGmo-S124
57.4 cgpGmo-S1461b

LGMAP 2

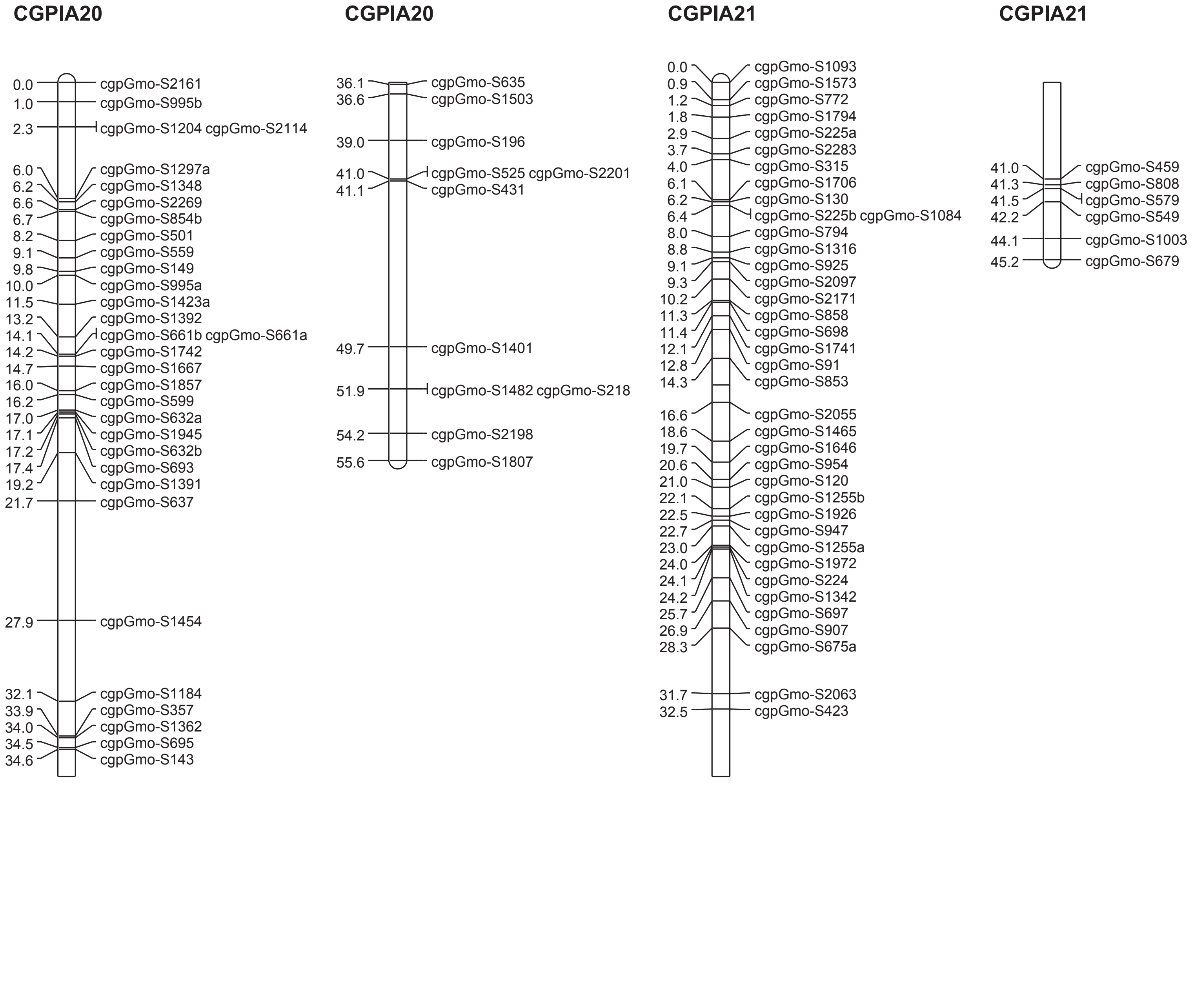
CGPIA20
0.0 cgpGmo-S2161
1.0 cgpGmo-S995b
2.3 cgpGmo-S1204 cgpGmo-S2114
6.0 cgpGmo-S1297a
6.2 cgpGmo-S1348
6.6 cgpGmo-S2269
6.7 cgpGmo-S854b
8.2 cgpGmo-S501
9.1 cgpGmo-S559
9.8 cgpGmo-S149
10.0 cgpGmo-S995a
11.5 cgpGmo-S1423a
13.2 cgpGmo-S1392
14.1 cgpGmo-S661b cgpGmo-S661a
14.2 cgpGmo-S1742
14.7 cgpGmo-S1667
16.0 cgpGmo-S1857
16.2 cgpGmo-S599
17.0 cgpGmo-S632a
17.1 cgpGmo-S1945
17.2 cgpGmo-S632b
17.4 cgpGmo-S693
19.2 cgpGmo-S1391
21.7 cgpGmo-S637
27.9 cgpGmo-S1454
32.1 cgpGmo-S1184
33.9 cgpGmo-S357
34.0 cgpGmo-S1362
34.5 cgpGmo-S695
34.6 cgpGmo-S143
CGPIA20
36.1 cgpGmo-S635
36.6 cgpGmo-S1503
39.0 cgpGmo-S196
41.0 cgpGmo-S525 cgpGmo-S2201
41.1 cgpGmo-S431
49.7 cgpGmo-S1401
51.9 cgpGmo-S1482 cgpGmo-S218
54.2 cgpGmo-S2198
55.6 cgpGmo-S1807
CGPIA21
0.0 cgpGmo-S1093
0.9 cgpGmo-S1573
1.2 cgpGmo-S772
1.8 cgpGmo-S1794
2.9 cgpGmo-S225a
3.7 cgpGmo-S2283
4.0 cgpGmo-S315
6.1 cgpGmo-S1706
6.2 cgpGmo-S130
6.4 cgpGmo-S225b cgpGmo-S1084
8.0 cgpGmo-S794
8.8 cgpGmo-S1316
9.1 cgpGmo-S925
9.3 cgpGmo-S2097
10.2 cgpGmo-S2171
11.3 cgpGmo-S858
11.4 cgpGmo-S698
12.1 cgpGmo-S1741
12.8 cgpGmo-S91
14.3 cgpGmo-S853
16.6 cgpGmo-S2055
18.6 cgpGmo-S1465
19.7 cgpGmo-S1646
20.6 cgpGmo-S954
21.0 cgpGmo-S120
22.1 cgpGmo-S1255b
22.5 cgpGmo-S1926
22.7 cgpGmo-S947
23.0 cgpGmo-S1255a
24.0 cgpGmo-S1972
24.1 cgpGmo-S224
24.2 cgpGmo-S1342
25.7 cgpGmo-S697
26.9 cgpGmo-S907
28.3 cgpGmo-S675a
31.7 cgpGmo-S2063
32.5 cgpGmo-S423
CGPIA21
41.0 cgpGmo-S459
41.3 cgpGmo-S808
41.5 cgpGmo-S579
42.2 cgpGmo-S549
44.1 cgpGmo-S1003
45.2 cgpGmo-S679

LGMAP 2

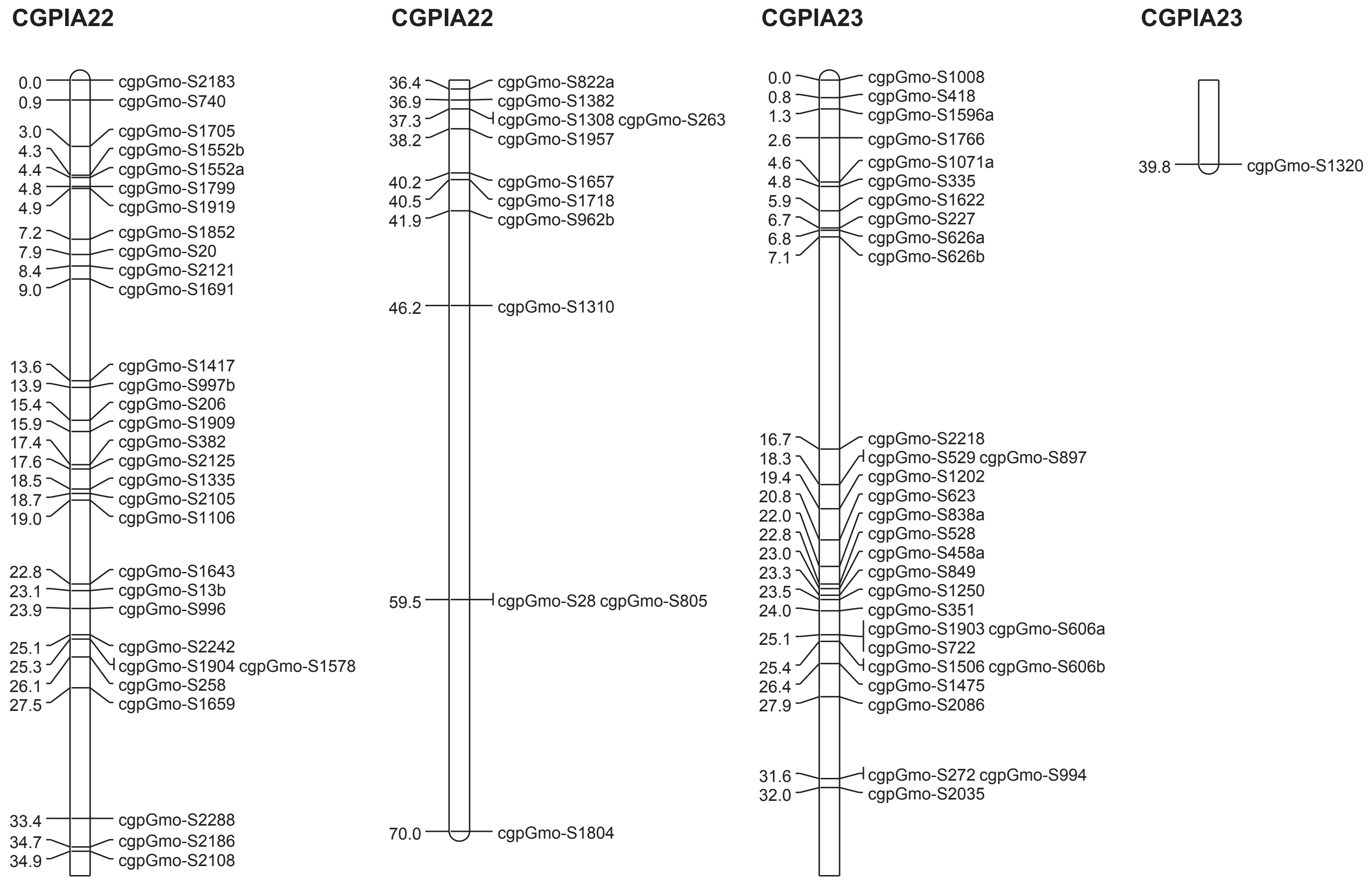
CGPIA22
0.0 cgpGmo-S2183
0.9 cgpGmo-S740
3.0 cgpGmo-S1705
4.3 cgpGmo-S1552b
4.4 cgpGmo-S1552a
4.8 cgpGmo-S1799
4.9 cgpGmo-S1919
7.2 cgpGmo-S1852
7.9 cgpGmo-S20
8.4 cgpGmo-S2121
9.0 cgpGmo-S1691
13.6 cgpGmo-S1417
13.9 cgpGmo-S997b
15.4 cgpGmo-S206
15.9 cgpGmo-S1909
17.4 cgpGmo-S382
17.6 cgpGmo-S2125
18.5 cgpGmo-S1335
18.7 cgpGmo-S2105
19.0 cgpGmo-S1106
22.8 cgpGmo-S1643
23.1 cgpGmo-S13b
23.9 cgpGmo-S996
25.1 cgpGmo-S2242
25.3 cgpGmo-S1904 cgpGmo-S1578
26.1 cgpGmo-S258
27.5 cgpGmo-S1659
33.4 cgpGmo-S2288
34.7 cgpGmo-S2186
34.9 cgpGmo-S2108
CGPIA22
36.4 cgpGmo-S822a
36.9 cgpGmo-S1382
37.3 cgpGmo-S1308 cgpGmo-S263
38.2 cgpGmo-S1957
40.2 cgpGmo-S1657
40.5 cgpGmo-S1718
41.9 cgpGmo-S962b
46.2 cgpGmo-S1310
59.5 cgpGmo-S28 cgpGmo-S805
70.0 cgpGmo-S1804
CGPIA23
0.0 cgpGmo-S1008
0.8 cgpGmo-S418
1.3 cgpGmo-S1596a
2.6 cgpGmo-S1766
4.6 cgpGmo-S1071a
4.8 cgpGmo-S335
5.9 cgpGmo-S1622
6.7 cgpGmo-S227
6.8 cgpGmo-S626a
7.1 cgpGmo-S626b
16.7 cgpGmo-S2218
18.3 cgpGmo-S529 cgpGmo-S897
19.4 cgpGmo-S1202
20.8 cgpGmo-S623
22.0 cgpGmo-S838a
22.8 cgpGmo-S528
23.0 cgpGmo-S458a
23.3 cgpGmo-S849
23.5 cgpGmo-S1250
24.0 cgpGmo-S351
25.1 cgpGmo-S1903 cgpGmo-S606a
cgpGmo-S722
25.4 cgpGmo-S1506 cgpGmo-S606b
26.4 cgpGmo-S1475
27.9 cgpGmo-S2086
31.6 cgpGmo-S272 cgpGmo-S994
32.0 cgpGmo-S2035
CGPIA23
39.8 cgpGmo-S1320
