# Supplementary material for: Genomic islands of divergence and their consequences for the resolution of spatial structure in an exploited marine fish
Source: Evol Appl. 2013 Jan 21;6(3):450–61. doi: 10.1111/eva.12026 (PMC3673473; doi:10.1111/eva.12026)
Supplement: Data S4 — Linkage maps 1 and 2. [file eva0006-0450-sd4.pdf]

Supplementary Materials Linkage Maps One and Two

LGMAP1			LGMAP2		
Locus	Group	Position	Locus	Group	Position
cgpGmo-S1438	LG1	0	cgpGmo-S1438	LG1	0
cgpGmo-S807	LG1	1.42	cgpGmo-S78	LG1	3.694
cgpGmo-S2068	LG1	1.788	cgpGmo-S564	LG1	3.963
cgpGmo-S940	LG1	3.325	cgpGmo-S2068	LG1	5.277
cgpGmo-S2254	LG1	3.513	cgpGmo-S105	LG1	5.357
cgpGmo-S1471	LG1	4.138	cgpGmo-S940	LG1	6.161
cgpGmo-S1393b	LG1	4.497	cgpGmo-S2254	LG1	6.203
cgpGmo-S1426	LG1	4.535	cgpGmo-S807	LG1	6.662
cgpGmo-S105	LG1	4.543	cgpGmo-S35a	LG1	6.97
cgpGmo-S1666	LG1	4.906	cgpGmo-S1471	LG1	7.128
cgpGmo-S1823	LG1	4.947	cgpGmo-S1393b	LG1	7.484
cgpGmo-S512	LG1	4.993	cgpGmo-S1426	LG1	7.497
cgpGmo-S1840	LG1	5.281	cgpGmo-S1666	LG1	7.932
cgpGmo-S1393a	LG1	5.397	cgpGmo-S512	LG1	7.97
cgpGmo-S387a	LG1	6.279	cgpGmo-S1823	LG1	8.004
cgpGmo-S1336	LG1	7.034	cgpGmo-S387a	LG1	8.296
cgpGmo-S1817	LG1	7.067	cgpGmo-S1393a	LG1	8.968
cgpGmo-S686a	LG1	7.429	cgpGmo-S1840	LG1	9.048
cgpGmo-S686b	LG1	7.429	cgpGmo-S1239	LG1	9.527
cgpGmo-S1369	LG1	7.527	cgpGmo-S1954	LG1	9.629
cgpGmo-S1407	LG1	8.068	cgpGmo-S1369	LG1	9.896
cgpGmo-S1239	LG1	8.434	cgpGmo-S1817	LG1	10.284
cgpGmo-S787	LG1	9.402	cgpGmo-S1336	LG1	10.786
cgpGmo-S1167	LG1	9.417	cgpGmo-S896	LG1	12.631
cgpGmo-S78	LG1	10.705	cgpGmo-S686a	LG1	12.772
cgpGmo-S270	LG1	11.952	cgpGmo-S686b	LG1	12.772
cgpGmo-S283	LG1	13.343	cgpGmo-S1788	LG1	13.57
cgpGmo-S1196b	LG1	13.75	cgpGmo-S1407	LG1	13.76
cgpGmo-S1853	LG1	14.736	cgpGmo-S1181	LG1	14.59
cgpGmo-S1365b	LG1	14.736	cgpGmo-S334	LG1	15.198
cgpGmo-S844	LG1	16.406	cgpGmo-S787	LG1	15.199
cgpGmo-S1806	LG1	18.104	cgpGmo-S1167	LG1	15.225
cgpGmo-S334	LG1	18.549	cgpGmo-S760	LG1	15.432
cgpGmo-S2021	LG1	20.544	cgpGmo-S283	LG1	15.438
cgpGmo-S1579	LG1	20.647	cgpGmo-S968	LG1	15.723
cgpGmo-S985	LG1	20.857	cgpGmo-S844	LG1	16.323
cgpGmo-S83	LG1	24.705	cgpGmo-S985	LG1	17.335
cgpGmo-S254	LG1	37.158	cgpGmo-S1703	LG1	18.073
cgpGmo-S407	LG1	41.37	cgpGmo-S1196b	LG1	18.208
cgpGmo-S360	LG1	42.326	cgpGmo-S270	LG1	18.299
cgpGmo-S603	LG1	42.942	cgpGmo-S694	LG1	18.378
cgpGmo-S2025	LG1	43.125	cgpGmo-S875b	LG1	19.072
cgpGmo-S292b	LG1	44.226	cgpGmo-S876	LG1	19.072
cgpGmo-S1969	LG1	44.616	cgpGmo-S1268	LG1	20.377

cgpGmo-S1224	LG1	45.722	cgpGmo-S1853	LG1	21.247
cgpGmo-S1845	LG1	46.206	cgpGmo-S1365b	LG1	21.247
cgpGmo-S1982	LG1	51.075	cgpGmo-S2021	LG1	22.074
cgpGmo-S1212	LG1	60.758	cgpGmo-S1806	LG1	22.49
cgpGmo-S749a	LG2	0	cgpGmo-S1365a	LG1	22.601
cgpGmo-S749b	LG2	2.192	cgpGmo-S1842	LG1	23.683
cgpGmo-S1338	LG2	2.686	cgpGmo-S291	LG1	26.33
cgpGmo-S2070	LG2	2.688	cgpGmo-S1579	LG1	26.884
cgpGmo-S535b	LG2	4.847	Pan_I_1	LG1	27.74
cgpGmo-S1274	LG2	5.279	cgpGmo-S773	LG1	28.171
cgpGmo-S1662	LG2	5.571	cgpGmo-S852	LG1	29.188
cgpGmo-S535a	LG2	6.443	cgpGmo-S605	LG1	29.406
cgpGmo-S604	LG2	8.783	cgpGmo-S83	LG1	29.419
cgpGmo-S1235	LG2	10.153	cgpGmo-S536	LG1	31.42
cgpGmo-S1879	LG2	10.268	cgpGmo-S1107	LG1	31.801
cgpGmo-S2264	LG2	10.564	cgpGmo-S1749	LG1	32.903
cgpGmo-S1997	LG2	10.885	cgpGmo-S523	LG1	34.206
cgpGmo-S2052	LG2	11.499	cgpGmo-S339	LG1	35.914
cgpGmo-S1563c	LG2	11.71	cgpGmo-S254	LG1	35.95
cgpGmo-S1677	LG2	11.998	cgpGmo-S1038	LG1	36.951
cgpGmo-S77	LG2	13.285	cgpGmo-S1801	LG1	37.353
cgpGmo-S1999	LG2	13.285	cgpGmo-S1683	LG1	37.809
cgpGmo-S1047	LG2	15.049	cgpGmo-S2082	LG1	37.809
cgpGmo-S185	LG2	15.077	cgpGmo-S603	LG1	38.36
cgpGmo-S951b	LG2	15.106	cgpGmo-S407	LG1	39.419
cgpGmo-S1743	LG2	15.234	cgpGmo-S828	LG1	39.933
cgpGmo-S1916	LG2	15.234	cgpGmo-S360	LG1	40.059
cgpGmo-S946	LG2	15.604	cgpGmo-S292b	LG1	41.148
cgpGmo-S338b	LG2	16.506	cgpGmo-S1969	LG1	41.223
cgpGmo-S155	LG2	19.127	cgpGmo-S1224	LG1	41.262
cgpGmo-S590	LG2	22.318	cgpGmo-S2025	LG1	42.231
cgpGmo-S1620	LG2	25.184	cgpGmo-S1845	LG1	42.455
cgpGmo-S1230a	LG2	26.123	cgpGmo-S1982	LG1	44.657
cgpGmo-S728	LG2	30.884	cgpGmo-S1087b	LG1	49.603
cgpGmo-S1221a	LG2	32.371	cgpGmo-S1212	LG1	56.022
cgpGmo-S1113	LG2	37.472	cgpGmo-S868	LG2	0
cgpGmo-S1112	LG2	37.472	cgpGmo-S754	LG2	1.201
cgpGmo-S1111	LG2	39.369	cgpGmo-S749a	LG2	1.248
cgpGmo-S2266	LG2	40.97	cgpGmo-S749b	LG2	2.599
cgpGmo-S318	LG2	43.058	cgpGmo-S1338	LG2	2.787
cgpGmo-S1231	LG2	43.767	cgpGmo-S305	LG2	3.239
cgpGmo-S1354	LG2	45.054	cgpGmo-S1274	LG2	4.642
cgpGmo-S810	LG2	46.758	cgpGmo-S2070	LG2	6.037
cgpGmo-S400	LG2	48.112	cgpGmo-S535b	LG2	6.469
cgpGmo-S2001	LG2	50.318	cgpGmo-S1662	LG2	7.01
cgpGmo-S333	LG2	50.692	cgpGmo-S1163	LG2	8.78
cgpGmo-S1284	LG2	51.415	cgpGmo-S535a	LG2	9.101

cgpGmo-S548	LG2	52.388	cgpGmo-S2157	LG2	9.349
cgpGmo-S1216b	LG2	52.415	cgpGmo-S604	LG2	10.259
cgpGmo-S68	LG2	56.275	cgpGmo-S2264	LG2	10.832
cgpGmo-S2112	LG2	57.182	cgpGmo-S1235	LG2	11.297
cgpGmo-S973	LG2	57.185	cgpGmo-S2052	LG2	11.651
cgpGmo-S1205	LG2	57.386	cgpGmo-S1677	LG2	12.241
cgpGmo-S1908	LG2	57.393	cgpGmo-S1997	LG2	13.113
cgpGmo-S454	LG2	57.428	cgpGmo-S1879	LG2	13.338
cgpGmo-S1026	LG2	57.444	cgpGmo-S77	LG2	13.883
cgpGmo-S1101a	LG2	57.527	cgpGmo-S1999	LG2	14.241
cgpGmo-S532	LG2	57.527	cgpGmo-S1910	LG2	14.465
cgpGmo-S1068	LG2	57.527	cgpGmo-S1563c	LG2	14.767
cgpGmo-S1456	LG2	57.527	cgpGmo-S1743	LG2	15.597
cgpGmo-S1022	LG2	57.622	cgpGmo-S1916	LG2	15.597
cgpGmo-S174	LG2	57.66	cgpGmo-S951b	LG2	15.893
cgpGmo-S184	LG2	57.66	cgpGmo-S946	LG2	16.421
cgpGmo-S1907	LG2	57.958	cgpGmo-S338b	LG2	17.12
cgpGmo-S182	LG2	57.958	cgpGmo-S185	LG2	17.501
cgpGmo-S489	LG2	58.602	cgpGmo-S16a	LG2	17.799
cgpGmo-S780	LG2	61.139	cgpGmo-S1047	LG2	18.155
cgpGmo-S444	LG3	0	cgpGmo-S155	LG2	19.509
cgpGmo-S81	LG3	5.028	cgpGmo-S1825	LG2	22.886
cgpGmo-S1652	LG3	10.711	cgpGmo-S590	LG2	23.464
cgpGmo-S2229	LG3	14.139	cgpGmo-S1230a	LG2	26.364
cgpGmo-S32	LG3	15.33	cgpGmo-S1620	LG2	27.304
cgpGmo-S2185	LG3	16.027	cgpGmo-S728	LG2	30.913
cgpGmo-S1808	LG3	20.151	cgpGmo-S1221a	LG2	34.434
cgpGmo-S171	LG3	22.675	cgpGmo-S1112	LG2	38.952
cgpGmo-S453	LG3	22.822	cgpGmo-S1113	LG2	38.952
cgpGmo-S2049	LG3	28.048	cgpGmo-S1693	LG2	39.158
cgpGmo-S716	LG3	29.875	cgpGmo-S1111	LG2	40.581
cgpGmo-S1757	LG3	30.046	HbBeta_1_Gary	LG2	40.84
cgpGmo-S769	LG3	30.803	cgpGmo-S2266	LG2	41.138
cgpGmo-S301	LG3	31.14	cgpGmo-S40	LG2	42.078
cgpGmo-S923	LG3	31.165	cgpGmo-S318	LG2	43.843
cgpGmo-S1007	LG3	31.424	cgpGmo-S1231	LG2	45.139
cgpGmo-S408	LG3	31.812	cgpGmo-S810	LG2	46.483
cgpGmo-S199	LG3	31.829	cgpGmo-S1354	LG2	46.671
cgpGmo-S1504	LG3	32.004	cgpGmo-S400	LG2	48.932
cgpGmo-S872b	LG3	32.192	cgpGmo-S2001	LG2	50.253
cgpGmo-S526	LG3	34.143	cgpGmo-S1216a	LG2	51.328
cgpGmo-S643b	LG3	36.605	cgpGmo-S1522	LG2	51.364
cgpGmo-S1927	LG3	44.739	cgpGmo-S333	LG2	51.575
cgpGmo-S478	LG3	45.823	cgpGmo-S1284	LG2	52.151
cgpGmo-S1789	LG3	45.971	cgpGmo-S1217	LG2	52.823
cgpGmo-S759	LG3	47.761	cgpGmo-S1216b	LG2	53.043
cgpGmo-S718a	LG3	47.913	cgpGmo-S548	LG2	53.149

cgpGmo-S801	LG3	47.913	cgpGmo-S68	LG2	57.131
cgpGmo-S718b	LG3	48.455	cgpGmo-S973	LG2	57.851
cgpGmo-S377	LG3	50.149	cgpGmo-S1908	LG2	57.865
cgpGmo-S1598b	LG3	50.678	cgpGmo-S2112	LG2	57.918
cgpGmo-S1967	LG3	53.534	cgpGmo-S1026	LG2	58.325
cgpGmo-S771	LG3	55.439	cgpGmo-S1205	LG2	58.399
cgpGmo-S1131	LG3	57.226	cgpGmo-S454	LG2	58.44
cgpGmo-S1656	LG3	57.355	cgpGmo-S1068	LG2	58.498
cgpGmo-S1218	LG3	59.883	cgpGmo-S1101a	LG2	58.498
cgpGmo-S1328	LG3	61.003	cgpGmo-S1456	LG2	58.498
cgpGmo-S1890	LG3	63.137	cgpGmo-S532	LG2	58.498
cgpGmo-S99	LG3	64.934	cgpGmo-S1022	LG2	58.695
cgpGmo-S799	LG3	66.886	cgpGmo-S184	LG2	58.702
cgpGmo-S734	LG3	67.432	cgpGmo-S174	LG2	58.702
cgpGmo-S1984	LG4	0	cgpGmo-S1751	LG2	58.95
cgpGmo-S204	LG4	0.981	cgpGmo-S182	LG2	59.231
cgpGmo-S552	LG4	2.251	cgpGmo-S1907	LG2	59.231
cgpGmo-S1730	LG4	3.703	cgpGmo-S2146	LG2	59.673
cgpGmo-S1739	LG4	3.921	cgpGmo-S489	LG2	60.088
cgpGmo-S657a	LG4	4.018	cgpGmo-S780	LG2	61.947
cgpGmo-S2155	LG4	6.432	cgpGmo-S444	LG3	0
cgpGmo-S2156	LG4	6.432	cgpGmo-S81	LG3	4.972
cgpGmo-S1491b	LG4	8.001	cgpGmo-S1652	LG3	5.891
cgpGmo-S1491a	LG4	9.233	cgpGmo-S514	LG3	8.209
cgpGmo-S1197a	LG4	10.852	cgpGmo-S1296	LG3	8.32
cgpGmo-S126a	LG4	11.52	cgpGmo-S466	LG3	9.136
cgpGmo-S1091	LG4	12.101	cgpGmo-S666	LG3	9.471
cgpGmo-S1833	LG4	13.105	cgpGmo-S1070	LG3	11.353
cgpGmo-S126b	LG4	14.6	cgpGmo-S2229	LG3	11.367
cgpGmo-S837	LG4	15.138	cgpGmo-S32	LG3	11.814
cgpGmo-S267	LG4	17.065	cgpGmo-S2185	LG3	12.691
cgpGmo-S395	LG4	17.214	cgpGmo-S646	LG3	15.256
cgpGmo-S1445	LG4	19.075	cgpGmo-S1808	LG3	17.731
cgpGmo-S1079	LG4	22.435	cgpGmo-S453	LG3	18.451
cgpGmo-S2279	LG4	24.832	cgpGmo-S171	LG3	21.033
cgpGmo-S1360b	LG4	25.092	cgpGmo-S172	LG3	21.092
cgpGmo-S1841	LG4	25.593	cgpGmo-S872a	LG3	21.099
cgpGmo-S1360a	LG4	25.617	cgpGmo-S491	LG3	21.815
cgpGmo-S791	LG4	25.617	cgpGmo-S526	LG3	22.227
5279C2CO1.498	LG4	26.563	cgpGmo-S430b	LG3	24.661
cgpGmo-S1979	LG4	26.859	cgpGmo-S1504	LG3	25.075
cgpGmo-S819b	LG4	27.284	cgpGmo-S408	LG3	25.377
cgpGmo-S434a	LG4	30.159	cgpGmo-S1007	LG3	25.722
cgpGmo-S701	LG4	32.947	cgpGmo-S872b	LG3	25.982
cgpGmo-S93	LG4	33.198	cgpGmo-S199	LG3	26.178
cgpGmo-S615	LG4	34.186	cgpGmo-S2049	LG3	28.242
cgpGmo-S354	LG4	34.303	cgpGmo-S301	LG3	28.417

cgpGmo-S1010	LG4	35.059	cgpGmo-S923	LG3	28.797
cgpGmo-S720	LG4	35.279	cgpGmo-S769	LG3	28.83
cgpGmo-S306a	LG4	35.983	cgpGmo-S1757	LG3	31.334
cgpGmo-S306b	LG4	35.983	cgpGmo-S1263	LG3	31.509
cgpGmo-S1768	LG4	36.255	cgpGmo-S716	LG3	32.058
cgpGmo-S1301	LG4	36.636	cgpGmo-S689	LG3	33.067
cgpGmo-S2079	LG4	37.559	cgpGmo-S643b	LG3	35.461
cgpGmo-S1865	LG4	37.717	cgpGmo-S398	LG3	37.446
cgpGmo-S420	LG4	37.802	cgpGmo-S755	LG3	38.783
cgpGmo-S543	LG4	37.802	cgpGmo-S1927	LG3	40.643
cgpGmo-S1744	LG4	38.346	cgpGmo-S643a	LG3	41.374
cgpGmo-S1856	LG4	38.843	cgpGmo-S478	LG3	41.852
cgpGmo-S167	LG4	43.499	cgpGmo-S1789	LG3	41.912
cgpGmo-S1698	LG4	58.836	cgpGmo-S759	LG3	43.129
cgpGmo-S2132	LG4	69.344	cgpGmo-S718a	LG3	43.313
cgpGmo-S2115	LG5	0	cgpGmo-S801	LG3	43.313
cgpGmo-S937	LG5	1.081	cgpGmo-S718b	LG3	43.801
cgpGmo-S129	LG5	2.91	cgpGmo-S1469	LG3	44.357
cgpGmo-S1745	LG5	3.366	cgpGmo-S1598b	LG3	46.11
cgpGmo-S58b	LG5	3.542	cgpGmo-S377	LG3	46.32
cgpGmo-S1241	LG5	4.33	cgpGmo-S22b	LG3	47.903
cgpGmo-S496	LG5	5.902	cgpGmo-S1967	LG3	49.118
cgpGmo-S1924	LG5	5.928	cgpGmo-S771	LG3	50.168
cgpGmo-S894	LG5	9.643	cgpGmo-S1656	LG3	52.481
cgpGmo-S2235	LG5	11.138	cgpGmo-S1131	LG3	52.907
cgpGmo-S2042	LG5	11.156	cgpGmo-S223	LG3	53.516
cgpGmo-S991a	LG5	12.299	cgpGmo-S644	LG3	55.18
cgpGmo-S162	LG5	12.564	cgpGmo-S1978	LG3	55.766
cgpGmo-S2083	LG5	12.886	cgpGmo-S1328	LG3	56.81
cgpGmo-S991b	LG5	13.144	cgpGmo-S1218	LG3	56.978
cgpGmo-S2196	LG5	13.15	cgpGmo-S1890	LG3	57.495
cgpGmo-S1771	LG5	13.193	cgpGmo-S2255	LG3	57.601
cgpGmo-S228	LG5	13.276	cgpGmo-S99	LG3	61.318
cgpGmo-S1169	LG5	13.346	cgpGmo-S799	LG3	62.346
cgpGmo-S310	LG5	13.349	cgpGmo-S734	LG3	63.426
cgpGmo-S1232	LG5	15.954	cgpGmo-S1984	LG4	0
cgpGmo-S239a	LG5	17.296	cgpGmo-S204	LG4	1.54
cgpGmo-S1519	LG5	30.185	cgpGmo-S552	LG4	1.94
cgpGmo-S1588b	LG5	31.167	cgpGmo-S1739	LG4	3.408
cgpGmo-S893	LG5	31.925	cgpGmo-S1730	LG4	3.441
cgpGmo-S640b	LG5	32.051	cgpGmo-S657a	LG4	4.177
cgpGmo-S137	LG5	32.861	cgpGmo-S2155	LG4	6.492
cgpGmo-S239b	LG5	35.711	cgpGmo-S2156	LG4	6.492
cgpGmo-S1942	LG5	36.763	cgpGmo-S1491b	LG4	7.972
cgpGmo-S2189	LG5	37.055	cgpGmo-S1491a	LG4	9.299
cgpGmo-S774	LG5	39.054	cgpGmo-S1197a	LG4	10.838
cgpGmo-S158b	LG5	39.296	cgpGmo-S126a	LG4	11.58

cgpGmo-S2123	LG5	40.404	cgpGmo-S1091	LG4	13.386
cgpGmo-S1158	LG5	41.163	cgpGmo-S1833	LG4	13.677
cgpGmo-S158a	LG5	42.159	cgpGmo-S126b	LG4	15.367
cgpGmo-S1672	LG5	43.556	cgpGmo-S837	LG4	16.181
cgpGmo-S1985	LG5	45.289	cgpGmo-S395	LG4	17.275
cgpGmo-S1452	LG5	53.834	cgpGmo-S267	LG4	17.494
cgpGmo-S404a	LG5	53.947	cgpGmo-S1445	LG4	19.231
cgpGmo-S1540	LG5	54.072	cgpGmo-S1079	LG4	22.325
cgpGmo-S1902	LG5	54.718	cgpGmo-S1841	LG4	24.971
cgpGmo-S800	LG5	54.879	cgpGmo-S2279	LG4	25.478
cgpGmo-S122	LG5	55.375	cgpGmo-S791	LG4	25.832
cgpGmo-S1078	LG5	55.577	cgpGmo-S1360a	LG4	25.832
cgpGmo-S1993	LG6	0	cgpGmo-S1360b	LG4	26.073
cgpGmo-S1538b	LG6	0.37	cgpGmo-S819b	LG4	26.476
cgpGmo-S1473	LG6	7.984	cgpGmo-S250	LG4	26.604
cgpGmo-S119a	LG6	8.656	5279C2CO1.498	LG4	26.863
cgpGmo-S848	LG6	10.484	cgpGmo-S1979	LG4	28.111
cgpGmo-S2165	LG6	10.673	cgpGmo-S792	LG4	30.108
cgpGmo-S2154	LG6	19.582	cgpGmo-S2056	LG4	30.153
cgpGmo-S1258a	LG6	20.891	cgpGmo-S205	LG4	30.606
cgpGmo-S764	LG6	23.855	cgpGmo-S434a	LG4	31.453
cgpGmo-S1086	LG6	35.942	cgpGmo-S434b	LG4	31.783
cgpGmo-S1252	LG6	38.232	cgpGmo-S93	LG4	34.024
cgpGmo-S1256b	LG6	39.623	cgpGmo-S701	LG4	34.099
cgpGmo-S1256a	LG6	39.631	cgpGmo-S1865	LG4	34.251
cgpGmo-S1332	LG6	40.057	cgpGmo-S1768	LG4	34.539
cgpGmo-S2065	LG6	40.121	cgpGmo-S354	LG4	34.811
cgpGmo-S537	LG6	40.29	cgpGmo-S615	LG4	35.236
cgpGmo-S2200	LG6	40.345	cgpGmo-S720	LG4	35.634
cgpGmo-S203	LG6	40.636	cgpGmo-S850	LG4	35.941
cgpGmo-S1062	LG6	40.639	cgpGmo-S1010	LG4	35.998
cgpGmo-S321	LG6	40.735	cgpGmo-S2079	LG4	36.198
cgpGmo-S389	LG6	40.826	cgpGmo-S306a	LG4	36.421
cgpGmo-S628	LG6	40.878	cgpGmo-S306b	LG4	36.421
cgpGmo-S714a	LG6	41.037	cgpGmo-S1301	LG4	37.29
cgpGmo-S2124	LG6	41.057	cgpGmo-S818a	LG4	37.32
cgpGmo-S1940	LG6	41.143	cgpGmo-S543	LG4	37.732
cgpGmo-S630	LG6	41.158	cgpGmo-S420	LG4	37.732
cgpGmo-S365b	LG6	41.335	cgpGmo-S1558	LG4	38.222
cgpGmo-S2081	LG6	41.675	cgpGmo-S1856	LG4	38.861
cgpGmo-S1872	LG6	43.845	cgpGmo-S134	LG4	39.085
cgpGmo-S1075	LG6	46.652	cgpGmo-S1744	LG4	39.515
cgpGmo-S72	LG6	46.652	cgpGmo-S2015	LG4	39.726
cgpGmo-S60	LG6	46.861	cgpGmo-S167	LG4	44.848
cgpGmo-S312	LG6	47.657	cgpGmo-S1698	LG4	59.164
cgpGmo-S2207	LG6	47.932	cgpGmo-S2132	LG4	69.706
cgpGmo-S768	LG6	48.954	cgpGmo-S938	LG5	0

cgpGmo-S277	LG6	49.802	cgpGmo-S2044	LG5	0.872
cgpGmo-S1463b	LG6	57.562	cgpGmo-S937	LG5	1.675
cgpGmo-S638b	LG6	58.253	cgpGmo-S58b	LG5	2.844
cgpGmo-S1721	LG6	58.479	cgpGmo-S129	LG5	3.287
cgpGmo-S173	LG6	59.482	cgpGmo-S2136	LG5	3.409
cgpGmo-S212	LG6	60.341	cgpGmo-S2115	LG5	3.57
cgpGmo-S1826	LG6	61.399	cgpGmo-S1745	LG5	4.848
cgpGmo-S2100	LG7	0	cgpGmo-S1162	LG5	4.865
cgpGmo-S1935	LG7	3.073	cgpGmo-S1241	LG5	5.046
cgpGmo-S26	LG7	3.484	cgpGmo-S496	LG5	6.768
cgpGmo-S1763	LG7	3.5	cgpGmo-S1924	LG5	7.038
cgpGmo-S255	LG7	4.126	cgpGmo-S2235	LG5	9.6
cgpGmo-S1906	LG7	4.379	cgpGmo-S894	LG5	10.205
cgpGmo-S1692	LG7	5.124	cgpGmo-S2042	LG5	11.241
cgpGmo-S895	LG7	5.319	cgpGmo-S990	LG5	11.923
cgpGmo-S282	LG7	5.432	cgpGmo-S2069	LG5	12.113
cgpGmo-S1399b	LG7	5.748	cgpGmo-S2196	LG5	12.122
cgpGmo-S393	LG7	5.9	cgpGmo-S1771	LG5	12.288
cgpGmo-S833	LG7	7.059	cgpGmo-S991a	LG5	12.726
cgpGmo-S1497	LG7	7.67	cgpGmo-S228	LG5	13.115
cgpGmo-S674	LG7	7.67	cgpGmo-S2083	LG5	13.39
cgpGmo-S1200	LG7	9.179	cgpGmo-S1169	LG5	13.392
cgpGmo-S877	LG7	10.037	cgpGmo-S310	LG5	13.394
cgpGmo-S2277	LG7	18.957	cgpGmo-S162	LG5	14.023
cgpGmo-S741	LG7	19.487	cgpGmo-S991b	LG5	14.227
cgpGmo-S917	LG7	24.996	cgpGmo-S1232	LG5	16
cgpGmo-S260a	LG7	28.281	cgpGmo-S977	LG5	26.336
cgpGmo-S426	LG7	29.735	cgpGmo-S239a	LG5	26.336
cgpGmo-S1644	LG7	31.937	cgpGmo-S1519	LG5	29.305
cgpGmo-S1065	LG7	32.691	cgpGmo-S1634	LG5	29.382
cgpGmo-S1668	LG7	36.457	cgpGmo-S1588b	LG5	29.919
cgpGmo-S207	LG7	36.576	cgpGmo-S1588a	LG5	30.015
cgpGmo-S992	LG7	37.531	cgpGmo-S1787	LG5	30.546
cgpGmo-S669	LG7	37.697	cgpGmo-S893	LG5	30.665
cgpGmo-S244	LG7	37.923	cgpGmo-S640b	LG5	30.741
cgpGmo-S62	LG7	38.596	cgpGmo-S137	LG5	31.141
cgpGmo-S63	LG7	38.724	cgpGmo-S82	LG5	31.141
cgpGmo-S2026	LG7	38.768	cgpGmo-S715	LG5	33.509
cgpGmo-S209	LG7	39.18	cgpGmo-S239b	LG5	34.351
cgpGmo-S889b	LG7	39.6	cgpGmo-S1942	LG5	35.493
cgpGmo-S869	LG7	41.712	cgpGmo-S774	LG5	37.607
cgpGmo-S2202	LG7	42.772	cgpGmo-S61	LG5	37.992
cgpGmo-S422	LG7	45.597	cgpGmo-S2189	LG5	38.06
cgpGmo-S831	LG7	46.386	cgpGmo-S1158	LG5	38.533
cgpGmo-S189	LG7	47.821	cgpGmo-S2111	LG5	39.324
cgpGmo-S385a	LG7	48.85	cgpGmo-S2123	LG5	39.769
cgpGmo-S830	LG7	48.971	cgpGmo-S2087	LG5	40.073

cgpGmo-S1858	LG7	49.03
cgpGmo-S2193	LG7	49.71
cgpGmo-S999	LG7	51.183
cgpGmo-S110	LG7	51.183
cgpGmo-S1782	LG7	51.967
cgpGmo-S452	LG7	65.233
cgpGmo-S595	LG8	0
cgpGmo-S1358	LG8	0.028
cgpGmo-S1050	LG8	0.55
cgpGmo-S1030	LG8	2.059
cgpGmo-S232b	LG8	3.137
cgpGmo-S232a	LG8	4.27
cgpGmo-S52	LG8	5.176
cgpGmo-S1287	LG8	6.462
cgpGmo-S412	LG8	8.116
cgpGmo-S748	LG8	9.356
cgpGmo-S776b	LG8	10.996
cgpGmo-S776a	LG8	11.182
cgpGmo-S2191	LG8	11.877
cgpGmo-S1785	LG8	12.29
cgpGmo-S1430a	LG8	13.398
cgpGmo-S1018a	LG8	13.627
cgpGmo-S597	LG8	13.73
cgpGmo-S1820	LG8	13.893
cgpGmo-S2002	LG8	14.026
cgpGmo-S421	LG8	14.035
cgpGmo-S786	LG8	14.035
cgpGmo-S1018b	LG8	14.521
cgpGmo-S362	LG8	14.623
cgpGmo-S332a	LG8	15.955
cgpGmo-S857	LG8	15.961
cgpGmo-S332b	LG8	18.028
cgpGmo-S1430b	LG8	21.521
cgpGmo-S1898	LG8	21.582
cgpGmo-S556	LG8	22.934
cgpGmo-S562	LG8	23.204
cgpGmo-S438	LG8	24.32
cgpGmo-S511b	LG8	24.564
cgpGmo-S1814	LG8	25.059
cgpGmo-S943	LG8	25.873
cgpGmo-S1714	LG8	26.788
cgpGmo-S891	LG8	29.198
cgpGmo-S396	LG8	30.314
cgpGmo-S1242	LG8	31.435
cgpGmo-S2059	LG8	31.699
cgpGmo-S1122	LG8	33.064
cgpGmo-S756a	LG8	33.759
cgpGmo-S158b	LG5	40.244
cgpGmo-S1816	LG5	40.666
cgpGmo-S158a	LG5	40.75
cgpGmo-S1607	LG5	41.075
cgpGmo-S1672	LG5	41.927
cgpGmo-S1985	LG5	45.067
cgpGmo-S725	LG5	49.237
cgpGmo-S404a	LG5	51.757
cgpGmo-S1452	LG5	51.801
cgpGmo-S1902	LG5	52.335
cgpGmo-S1540	LG5	52.52
cgpGmo-S1078	LG5	52.626
cgpGmo-S800	LG5	53.602
cgpGmo-S122	LG5	54.637
cgpGmo-S1993	LG6	0
cgpGmo-S1538b	LG6	0.696
cgpGmo-S347	LG6	2.959
cgpGmo-S1359	LG6	3.576
cgpGmo-S119b	LG6	4.11
cgpGmo-S119a	LG6	4.451
cgpGmo-S2165	LG6	6.176
cgpGmo-S848	LG6	6.334
cgpGmo-S2172	LG6	7.086
cgpGmo-S1473	LG6	9.907
cgpGmo-S1538a	LG6	13.475
cgpGmo-S2154	LG6	16.657
cgpGmo-S1258a	LG6	17.945
cgpGmo-S764	LG6	19.612
cgpGmo-S88b	LG6	19.965
cgpGmo-S1258b	LG6	20.829
cgpGmo-S530a	LG6	24.762
cgpGmo-S1887	LG6	27.868
cgpGmo-S1086	LG6	32.008
cgpGmo-S1510	LG6	33.653
cgpGmo-S1252	LG6	34.53
cgpGmo-S1256b	LG6	35.75
cgpGmo-S1256a	LG6	35.752
cgpGmo-S1332	LG6	36.028
cgpGmo-S2124	LG6	36.162
cgpGmo-S2200	LG6	36.406
cgpGmo-S2065	LG6	36.417
cgpGmo-S1062	LG6	36.725
cgpGmo-S321	LG6	37.073
cgpGmo-S1940	LG6	37.095
cgpGmo-S628	LG6	37.183
cgpGmo-S714a	LG6	37.215
cgpGmo-S630	LG6	37.241

cgpGmo-S550	LG8	34.83	cgpGmo-S365b	LG6	37.531
cgpGmo-S509	LG8	35.592	cgpGmo-S389	LG6	37.607
cgpGmo-S311b	LG8	36.247	cgpGmo-S537	LG6	38.848
cgpGmo-S1891	LG8	38.932	cgpGmo-S2081	LG6	38.927
cgpGmo-S1179	LG8	39.663	cgpGmo-S203	LG6	39.797
cgpGmo-S284	LG8	39.818	cgpGmo-S1872	LG6	40.625
cgpGmo-S1085a	LG8	39.818	cgpGmo-S930	LG6	40.725
cgpGmo-S2089	LG8	40.775	cgpGmo-S785b	LG6	40.725
cgpGmo-S45	LG8	46.181	cgpGmo-S470	LG6	41.955
cgpGmo-S751	LG8	47.108	cgpGmo-S1075	LG6	43.595
cgpGmo-S1708	LG8	48.931	cgpGmo-S72	LG6	43.595
cgpGmo-S1276a	LG8	49.719	cgpGmo-S60	LG6	43.763
cgpGmo-S1748	LG8	50.039	cgpGmo-S2207	LG6	43.922
cgpGmo-S2054	LG8	50.394	cgpGmo-S312	LG6	44.736
cgpGmo-S1779	LG8	50.394	cgpGmo-S768	LG6	45.066
cgpGmo-S1713	LG8	51.163	cgpGmo-S277	LG6	46.003
cgpGmo-S383	LG8	51.451	cgpGmo-S1687	LG6	47.678
cgpGmo-S2104	LG8	51.779	cgpGmo-S672	LG6	48.209
cgpGmo-S1572	LC9	0	cgpGmo-S1629	LG6	48.779
cgpGmo-S2180	LC9	0.958	cgpGmo-S2176	LG6	48.797
cgpGmo-S553	LC9	1.007	cgpGmo-S638a	LG6	50.216
cgpGmo-S1735	LC9	1.259	cgpGmo-S1813	LG6	50.216
cgpGmo-S201	LC9	1.373	cgpGmo-S1777	LG6	52.203
cgpGmo-S658	LC9	2.487	cgpGmo-S1463b	LG6	53.421
cgpGmo-S1237	LC9	3.283	cgpGmo-S638b	LG6	54.132
cgpGmo-S127	LC9	3.872	cgpGmo-S1721	LG6	54.26
cgpGmo-S998	LC9	4.288	cgpGmo-S121	LG6	54.335
cgpGmo-S953	LC9	5.553	cgpGmo-S173	LG6	54.612
cgpGmo-S1123	LC9	7.257	cgpGmo-S212	LG6	55.854
cgpGmo-S341	LC9	9.057	cgpGmo-S1826	LG6	58.537
cgpGmo-S1704	LC9	11.181	cgpGmo-S2119	LG6	63.958
cgpGmo-S114	LC9	12.469	cgpGmo-S2100	LG7	0
cgpGmo-S259	LC9	13.675	cgpGmo-S26	LG7	2.09
cgpGmo-S429	LC9	14.824	cgpGmo-S1935	LG7	3.488
cgpGmo-S770	LC9	20.426	cgpGmo-S1763	LG7	4.414
cgpGmo-S447	LC9	20.786	cgpGmo-S255	LG7	4.615
cgpGmo-S30	LC9	26.956	cgpGmo-S1906	LG7	4.785
cgpGmo-S1507	LC9	27.601	cgpGmo-S282	LG7	4.905
cgpGmo-S18	LC9	28.583	cgpGmo-S393	LG7	5.048
cgpGmo-S361	LC9	29.878	cgpGmo-S1692	LG7	5.624
cgpGmo-S1013	LC9	30.955	cgpGmo-S976b	LG7	5.895
cgpGmo-S413	LC9	31.132	cgpGmo-S895	LG7	5.898
cgpGmo-S1442	LC9	32.522	cgpGmo-S833	LG7	6.281
cgpGmo-S1017	LC9	38.072	cgpGmo-S1497	LG7	6.557
cgpGmo-S1965	LC9	41.27	cgpGmo-S674	LG7	6.557
cgpGmo-S730	LC9	41.653	cgpGmo-S834	LG7	6.578
cgpGmo-S410	LC9	42.208	cgpGmo-S1399b	LG7	7.291

cgpGmo-S1001	LC9	42.239	cgpGmo-S1859	LG7	7.4
cgpGmo-S376	LC9	42.735	cgpGmo-S1867	LG7	7.584
cgpGmo-S544	LC9	42.824	cgpGmo-S1399a	LG7	7.748
cgpGmo-S449b	LC9	43.283	cgpGmo-S1279	LG7	8.19
cgpGmo-S1031	LC9	43.536	cgpGmo-S1200	LG7	9.353
cgpGmo-S703	LC9	43.728	cgpGmo-S877	LG7	10.198
cgpGmo-S435	LC9	44.021	cgpGmo-S741	LG7	15.669
cgpGmo-S2016	LC9	44.301	cgpGmo-S2277	LG7	16.328
cgpGmo-S682	LC9	44.4	cgpGmo-S2019	LG7	17.655
cgpGmo-S609	LC9	44.814	cgpGmo-S917	LG7	18.442
cgpGmo-S1412	LC9	45.507	cgpGmo-S870	LG7	19.4
cgpGmo-S1045	LC9	45.507	cgpGmo-S268	LG7	19.4
cgpGmo-S1178	LC9	46.127	cgpGmo-S1991	LG7	19.4
cgpGmo-S719	LC9	47.13	cgpGmo-S152	LG7	19.4
cgpGmo-S948	LC9	47.392	cgpGmo-S1810	LG7	19.4
cgpGmo-S1377	LC9	47.498	cgpGmo-S1830	LG7	19.4
cgpGmo-S342	LC9	51.858	cgpGmo-S739	LG7	19.4
cgpGmo-S1011b	LC9	53.744	cgpGmo-S157	LG7	19.4
cgpGmo-S1011a	LC9	53.744	cgpGmo-S1183	LG7	19.4
cgpGmo-S1513	LC9	62.294	cgpGmo-S814a	LG7	19.4
cgpGmo-S746	LC9	65.337	cgpGmo-S1089	LG7	19.4
cgpGmo-S802	LG10	0	cgpGmo-S183	LG7	19.4
cgpGmo-S864	LG10	19.907	cgpGmo-S673	LG7	19.4
cgpGmo-S1832	LG10	22.436	cgpGmo-S1039a	LG7	19.4
cgpGmo-S135	LG10	36.83	cgpGmo-S2158	LG7	19.4
cgpGmo-S1929	LG10	38.104	cgpGmo-S419	LG7	19.4
cgpGmo-S1836	LG10	40.574	cgpGmo-S920	LG7	19.4
cgpGmo-S1304b	LG10	45.489	cgpGmo-S1039b	LG7	19.4
cgpGmo-S775	LG10	49.341	cgpGmo-S352	LG7	19.4
cgpGmo-S2107	LG10	50.84	cgpGmo-S1425	LG7	19.4
cgpGmo-S1556	LG10	51.325	cgpGmo-S982a	LG7	19.4
cgpGmo-S37a	LG10	51.423	cgpGmo-S260a	LG7	20.315
cgpGmo-S37b	LG10	51.427	cgpGmo-S426	LG7	20.599
cgpGmo-S327	LG10	51.793	cgpGmo-S1644	LG7	22.53
cgpGmo-S479	LG10	52.101	cgpGmo-S1065	LG7	24.679
cgpGmo-S921	LG10	52.396	cgpGmo-S207	LG7	27.244
cgpGmo-S25	LG10	54.848	cgpGmo-S1668	LG7	27.434
cgpGmo-S575	LG10	55.6	cgpGmo-S62	LG7	27.458
cgpGmo-S336	LG10	57.193	cgpGmo-S244	LG7	28.137
cgpGmo-S367	LG10	59.019	cgpGmo-S209	LG7	28.284
cgpGmo-S94	LG10	60.487	cgpGmo-S669	LG7	28.602
cgpGmo-S513	LG10	60.63	cgpGmo-S889b	LG7	28.732
cgpGmo-S668	LG10	62.556	cgpGmo-S63	LG7	29.038
cgpGmo-S1866	LG10	62.656	cgpGmo-S992	LG7	29.04
cgpGmo-S363	LG10	78.189	cgpGmo-S2026	LG7	29.112
cgpGmo-S1104	LG10	78.189	cgpGmo-S2202	LG7	32.49
cgpGmo-S1455	LG10	78.189	cgpGmo-S869	LG7	32.557

cgpGmo-S1024	LG11	0	cgpGmo-S1858	LG7	34.991
cgpGmo-S390b	LG11	4.595	cgpGmo-S584	LG7	35.231
cgpGmo-S945a	LG11	5.887	cgpGmo-S422	LG7	35.232
cgpGmo-S245b	LG11	7.488	cgpGmo-S831	LG7	35.796
cgpGmo-S245a	LG11	7.733	cgpGmo-S385a	LG7	38.236
cgpGmo-S670	LG11	8.98	cgpGmo-S189	LG7	38.446
cgpGmo-S1609a	LG11	9.293	cgpGmo-S830	LG7	38.902
cgpGmo-S403	LG11	14.467	cgpGmo-S2193	LG7	39.108
cgpGmo-S1272	LG11	14.473	cgpGmo-S1058	LG7	39.303
cgpGmo-S967b	LG11	14.473	cgpGmo-S999	LG7	40.877
cgpGmo-S967a	LG11	15.24	cgpGmo-S110	LG7	40.877
cgpGmo-S1948	LG11	16.782	cgpGmo-S1782	LG7	41.329
cgpGmo-S1733	LG11	19.468	cgpGmo-S2134	LG7	42.946
cgpGmo-S1484	LG11	28.514	cgpGmo-S452	LG7	44.968
cgpGmo-S939	LG11	30.067	cgpGmo-S595	LG8	0
cgpGmo-S424	LG11	36.518	cgpGmo-S1358	LG8	0.031
cgpGmo-S618	LG11	37.79	cgpGmo-S1050	LG8	0.551
cgpGmo-S1222	LG11	41.831	cgpGmo-S1030	LG8	2.041
cgpGmo-S691	LG11	45.616	cgpGmo-S232b	LG8	3.145
cgpGmo-S867	LG11	46.121	cgpGmo-S232a	LG8	4.257
cgpGmo-S1647	LG11	47.52	cgpGmo-S52	LG8	5.173
cgpGmo-S811a	LG11	47.952	cgpGmo-S1287	LG8	6.491
cgpGmo-S1658	LG11	48.778	cgpGmo-S1747	LG8	7.262
cgpGmo-S4	LG11	49.777	cgpGmo-S412	LG8	8.235
cgpGmo-S634	LG11	50.204	cgpGmo-S748	LG8	9.413
cgpGmo-S150	LG11	51.303	cgpGmo-S776a	LG8	11.039
cgpGmo-S2179	LG11	51.606	cgpGmo-S776b	LG8	11.088
cgpGmo-S1998	LG11	52.324	cgpGmo-S2191	LG8	11.801
cgpGmo-S717	LG11	52.839	cgpGmo-S1785	LG8	12.304
cgpGmo-S1767	LG11	52.935	cgpGmo-S1018a	LG8	13.355
cgpGmo-S79	LG11	53.745	cgpGmo-S597	LG8	13.683
cgpGmo-S1431a	LG11	55.138	cgpGmo-S1820	LG8	13.794
cgpGmo-S2017	LG11	55.178	cgpGmo-S2002	LG8	14.001
cgpGmo-S1712	LG11	55.182	cgpGmo-S1430a	LG8	14.054
cgpGmo-S788	LG11	55.197	cgpGmo-S786	LG8	14.226
cgpGmo-S2211	LG11	55.392	cgpGmo-S421	LG8	14.226
cgpGmo-S1843	LG11	55.587	cgpGmo-S362	LG8	14.51
cgpGmo-S2159	LG11	55.726	cgpGmo-S1018b	LG8	14.824
cgpGmo-S707	LG11	55.76	cgpGmo-S332a	LG8	16.007
cgpGmo-S2005	LG11	56.033	cgpGmo-S857	LG8	16.084
cgpGmo-S1090	LG11	56.362	cgpGmo-S332b	LG8	17.992
cgpGmo-S587	LG11	56.639	cgpGmo-S1898	LG8	21.791
cgpGmo-S2102	LG11	56.671	cgpGmo-S1430b	LG8	21.848
cgpGmo-S607	LG11	56.688	cgpGmo-S556	LG8	22.503
cgpGmo-S386	LG11	57.898	cgpGmo-S562	LG8	22.807
cgpGmo-S488	LG11	57.936	cgpGmo-S438	LG8	24.379
cgpGmo-S922	LG11	58.456	cgpGmo-S511b	LG8	24.609

cgpGmo-S44	LG11	58.763	cgpGmo-S1814	LG8	25.025
cgpGmo-S416b	LG11	59.854	cgpGmo-S943	LG8	25.705
cgpGmo-S416a	LG11	60.585	cgpGmo-S1714	LG8	27.497
cgpGmo-S581	LG11	60.629	cgpGmo-S891	LG8	29.387
cgpGmo-S580	LG11	61.141	cgpGmo-S396	LG8	30.044
cgpGmo-S1384	LG11	68.647	cgpGmo-S1242	LG8	31.708
cgpGmo-S1956	LG12	0	cgpGmo-S2059	LG8	31.817
cgpGmo-S521b	LG12	0.55	cgpGmo-S1085b	LG8	32.884
cgpGmo-S476	LG12	1.557	cgpGmo-S1122	LG8	33.503
cgpGmo-S1226	LG12	6.164	cgpGmo-S756a	LG8	34.303
cgpGmo-S275	LG12	7.229	cgpGmo-S550	LG8	34.63
cgpGmo-S582	LG12	7.661	cgpGmo-S509	LG8	35.495
cgpGmo-S936	LG12	8.363	cgpGmo-S311b	LG8	36.696
cgpGmo-S57	LG12	17.5	cgpGmo-S1891	LG8	38.924
cgpGmo-S914	LG12	18.233	cgpGmo-S1179	LG8	39.572
cgpGmo-S688	LG12	18.403	cgpGmo-S2089	LG8	40.427
cgpGmo-S2034	LG12	18.679	cgpGmo-S1085a	LG8	40.441
cgpGmo-S314	LG12	18.795	cgpGmo-S284	LG8	40.441
cgpGmo-S1543	LG12	19.001	cgpGmo-S2222	LG8	42.779
cgpGmo-S1260	LG12	19.001	cgpGmo-S1553a	LG8	43.633
cgpGmo-S816a	LG12	19.001	cgpGmo-S45	LG8	46.025
cgpGmo-S372a	LG12	19.001	cgpGmo-S751	LG8	47.099
cgpGmo-S180b	LG12	19.001	cgpGmo-S1370	LG8	47.158
cgpGmo-S486	LG12	19.001	cgpGmo-S1708	LG8	48.478
cgpGmo-S233	LG12	19.155	cgpGmo-S1276a	LG8	49.201
cgpGmo-S116	LG12	19.257	cgpGmo-S1748	LG8	49.941
cgpGmo-S510	LG12	19.448	cgpGmo-S2054	LG8	50.585
cgpGmo-S1737	LG12	19.615	cgpGmo-S1779	LG8	50.585
cgpGmo-S493	LG12	19.794	cgpGmo-S1713	LG8	51.467
cgpGmo-S417	LG12	19.952	cgpGmo-S2104	LG8	51.507
cgpGmo-S229	LG12	19.999	cgpGmo-S383	LG8	51.719
cgpGmo-S1882	LG12	20.003	cgpGmo-S1341	LG8	52.455
cgpGmo-S1689	LG12	20.003	cgpGmo-S2144	LG8	55.333
cgpGmo-S2032	LG12	20.009	cgpGmo-S779	LC9	-0.005
cgpGmo-S190	LG12	20.74	cgpGmo-S882a	LC9	0
cgpGmo-S1696	LG12	21.244	cgpGmo-S1572	LC9	3.318
cgpGmo-S502	LG12	22.041	cgpGmo-S553	LC9	4.049
cgpGmo-S1769	LG12	22.44	cgpGmo-S2180	LC9	4.291
cgpGmo-S256	LG12	23.772	cgpGmo-S1735	LC9	4.487
cgpGmo-S742a	LG12	45.919	cgpGmo-S201	LC9	4.709
cgpGmo-S348	LG13	0	cgpGmo-S658	LC9	5.904
cgpGmo-S2177	LG13	6.37	cgpGmo-S1237	LC9	6.431
cgpGmo-S1653	LG13	7.985	cgpGmo-S127	LC9	6.934
cgpGmo-S294	LG13	9.378	cgpGmo-S998	LC9	7.645
cgpGmo-S1483	LG13	9.808	cgpGmo-S953	LC9	9.15
cgpGmo-S1206	LG13	10.567	cgpGmo-S1123	LC9	10.257
cgpGmo-S2215	LG13	11.36	cgpGmo-S341	LC9	12.431

cgpGmo-S191	LG13	11.614	cgpGmo-S1704	LC9	15.166
cgpGmo-S652	LG13	12.227	cgpGmo-S114	LC9	15.765
cgpGmo-S1695	LG13	12.432	cgpGmo-S259	LC9	16.791
cgpGmo-S692a	LG13	12.751	cgpGmo-S429	LC9	18.741
cgpGmo-S2067	LG13	13.645	cgpGmo-S447	LC9	23.617
cgpGmo-S576	LG13	15.252	cgpGmo-S770	LC9	24.84
cgpGmo-S949a	LG13	18.506	cgpGmo-S1507	LC9	30.893
cgpGmo-S1097	LG13	25.022	cgpGmo-S30	LC9	31
cgpGmo-S1069	LG13	27.422	cgpGmo-S18	LC9	32.5
cgpGmo-S980	LG13	27.422	cgpGmo-S361	LC9	33.437
cgpGmo-S1889	LG13	28.108	cgpGmo-S413	LC9	33.953
cgpGmo-S2058	LG13	32.207	cgpGmo-S1013	LC9	35.193
cgpGmo-S1066	LG13	33.81	cgpGmo-S1442	LC9	36.517
cgpGmo-S752b	LG13	34.58	cgpGmo-S572	LC9	37.238
cgpGmo-S1390a	LG13	36.092	cgpGmo-S578	LC9	37.346
cgpGmo-S1961	LG13	36.104	cgpGmo-S89a	LC9	40.308
cgpGmo-S752a	LG13	38.48	cgpGmo-S2173	LC9	40.573
cgpGmo-S399	LG13	39.357	cgpGmo-S159	LC9	40.96
cgpGmo-S1990	LG13	42.322	cgpGmo-S1017	LC9	41.614
cgpGmo-S2018	LG13	45.268	cgpGmo-S1965	LC9	43.286
cgpGmo-S29	LG13	45.436	cgpGmo-S1001	LC9	44.5
cgpGmo-S906	LG13	46.488	cgpGmo-S682	LC9	44.607
cgpGmo-S36b	LG13	46.77	cgpGmo-S376	LC9	45.233
cgpGmo-S2013	LG13	47.746	cgpGmo-S449b	LC9	45.606
cgpGmo-S350	LG13	48.81	cgpGmo-S410	LC9	45.623
cgpGmo-S1981	LG13	49.898	cgpGmo-S544	LC9	46.289
cgpGmo-S487	LG13	50.439	cgpGmo-S1031	LC9	46.507
cgpGmo-S765	LG13	51.57	cgpGmo-S2016	LC9	46.814
cgpGmo-S1959	LG13	51.702	cgpGmo-S703	LC9	47.066
cgpGmo-S2281	LG13	52.071	cgpGmo-S435	LC9	47.516
cgpGmo-S1563	LG13	52.37	cgpGmo-S609	LC9	47.914
cgpGmo-S1762	LG13	52.651	cgpGmo-S948	LC9	49.022
cgpGmo-S881	LG13	52.733	cgpGmo-S309	LC9	49.315
cgpGmo-S1639	LG13	52.784	cgpGmo-S730	LC9	49.91
cgpGmo-S905	LG13	52.784	cgpGmo-S719	LC9	50.048
cgpGmo-S1209	LG13	53.23	cgpGmo-S1045	LC9	50.106
cgpGmo-S614b	LG13	53.447	cgpGmo-S1412	LC9	50.106
cgpGmo-S107	LG13	53.573	cgpGmo-S1178	LC9	50.557
cgpGmo-S614a	LG13	53.886	cgpGmo-S1839	LC9	50.684
cgpGmo-S793a	LG13	54.24	cgpGmo-S874	LC9	51.985
cgpGmo-S2160	LG13	54.246	cgpGmo-S1377	LC9	52.061
cgpGmo-S2028	LG13	54.579	cgpGmo-S1092a	LC9	53.383
cgpGmo-S220	LG13	55.357	cgpGmo-S342	LC9	55.144
cgpGmo-S1720	LG13	58.278	cgpGmo-S546	LC9	55.207
cgpGmo-S2039	LG13	58.481	cgpGmo-S986	LC9	56.378
cgpGmo-S217a	LG13	69.705	cgpGmo-S1157	LC9	56.38
cgpGmo-S1977	LG13	70.268	cgpGmo-S1011a	LC9	57.453

cgpGmo-S505	LG14	0	cgpGmo-S1011b	LC9	57.453
cgpGmo-S1760	LG14	2.424	cgpGmo-S1513	LC9	62.189
cgpGmo-S988	LG14	3.445	cgpGmo-S746	LC9	63.268
cgpGmo-S963	LG14	4.838	cgpGmo-S802	LG10	0
cgpGmo-S841	LG14	5.439	cgpGmo-S1832	LG10	1.5
cgpGmo-S796	LG14	5.629	cgpGmo-S115	LG10	2.829
cgpGmo-S1697	LG14	6.474	cgpGmo-S135	LG10	5.041
cgpGmo-S577	LG14	6.77	cgpGmo-S1076a	LG10	7.425
cgpGmo-S631	LG14	6.925	cgpGmo-S2182	LG10	7.816
cgpGmo-S2110	LG14	8.467	cgpGmo-S1943	LG10	8.36
cgpGmo-S1922	LG14	9.659	cgpGmo-S864	LG10	8.477
cgpGmo-S252	LG14	10.941	cgpGmo-S425	LG10	8.904
cgpGmo-S427	LG14	12.038	cgpGmo-S1929	LG10	8.952
cgpGmo-S462	LG14	12.387	cgpGmo-S1034	LG10	9.187
cgpGmo-S1467	LG14	13.581	cgpGmo-S516	LG10	9.354
cgpGmo-S617	LG14	14.159	cgpGmo-S942	LG10	9.47
cgpGmo-S1466a	LG14	16.566	cgpGmo-S1344	LG10	9.777
cgpGmo-S1466b	LG14	16.566	cgpGmo-S668	LG10	10.113
cgpGmo-S1914	LG14	17.373	cgpGmo-S1654	LG10	10.179
cgpGmo-S932b	LG14	18.079	cgpGmo-S1025	LG10	10.336
cgpGmo-S1049	LG14	23.336	cgpGmo-S1869	LG10	11.874
cgpGmo-S1792	LG14	24.069	cgpGmo-S778	LG10	13.246
cgpGmo-S1701	LG14	24.647	cgpGmo-S2012	LG10	14.223
cgpGmo-S1234	LG14	26.958	cgpGmo-S37b	LG10	16.423
cgpGmo-S302	LG14	27.718	cgpGmo-S37a	LG10	16.539
cgpGmo-S240	LG14	27.783	cgpGmo-S327	LG10	16.744
cgpGmo-S1888	LG14	29.002	cgpGmo-S2107	LG10	17.336
cgpGmo-S1988	LG14	29.111	cgpGmo-S1556	LG10	17.362
cgpGmo-S520	LG14	29.635	cgpGmo-S775	LG10	17.696
cgpGmo-S1424b	LG14	30.118	cgpGmo-S479	LG10	17.821
cgpGmo-S411	LG14	30.581	cgpGmo-S921	LG10	18.14
cgpGmo-S2078	LG14	30.692	cgpGmo-S25	LG10	18.485
cgpGmo-S70	LG14	31.003	cgpGmo-S575	LG10	22.589
cgpGmo-S827	LG14	31.632	cgpGmo-S448	LG10	23.205
cgpGmo-S1080	LG14	33.044	cgpGmo-S1273	LG10	23.337
cgpGmo-S226	LG14	33.5	cgpGmo-S367	LG10	24.096
cgpGmo-S1394a	LG14	36.201	cgpGmo-S336	LG10	24.31
cgpGmo-S1280	LG14	36.926	cgpGmo-S215	LG10	24.95
cgpGmo-S965	LG14	37.621	cgpGmo-S1304b	LG10	25.379
1057C1CO1.398	LG14	37.84	cgpGmo-S1900	LG10	26.066
cgpGmo-S1186	LG14	38.132	cgpGmo-S1836	LG10	26.368
cgpGmo-S824	LG14	46.559	cgpGmo-S313	LG10	26.489
cgpGmo-S711b	LG14	47.315	cgpGmo-S153a	LG10	26.539
cgpGmo-S142	LG14	47.493	cgpGmo-S1304a	LG10	26.814
cgpGmo-S503	LG14	49.433	cgpGmo-S1778	LG10	27.496
cgpGmo-S249	LG14	54.945	cgpGmo-S1327a	LG10	27.766
cgpGmo-S583	LG14	56.1	cgpGmo-S471	LG10	28.152

cgpGmo-S551	LG14	57.293	cgpGmo-S1334	LG10	28.175
cgpGmo-S1783	LG14	57.663	cgpGmo-S1410	LG10	28.763
cgpGmo-S92	LG15	3.029	cgpGmo-S371	LG10	29.08
cgpGmo-S1048	LG15	12.046	cgpGmo-S49	LG10	30.238
cgpGmo-S1770	LG15	28.895	cgpGmo-S1490	LG10	30.604
cgpGmo-S2093	LG15	28.895	cgpGmo-S2153	LG10	30.9
cgpGmo-S677	LG15	28.941	cgpGmo-S1098	LG10	31.66
cgpGmo-S298	LG15	31.846	cgpGmo-S1866	LG10	32.181
cgpGmo-S608	LG15	32.345	cgpGmo-S513	LG10	33.648
cgpGmo-S676	LG15	32.572	cgpGmo-S1104	LG10	33.773
cgpGmo-S1621	LG15	33.007	cgpGmo-S363	LG10	33.773
cgpGmo-S591	LG15	33.464	cgpGmo-S1455	LG10	33.773
cgpGmo-S1781	LG15	37.058	cgpGmo-S94	LG10	33.918
cgpGmo-S1728	LG15	38.628	cgpGmo-S1024	LG11	0
cgpGmo-S629	LG15	39.329	cgpGmo-S390b	LG11	4.573
cgpGmo-S1577	LG15	39.849	cgpGmo-S945a	LG11	6.296
cgpGmo-S1899	LG15	39.985	cgpGmo-S245b	LG11	8.294
cgpGmo-S1896	LG15	41.486	cgpGmo-S245a	LG11	8.629
cgpGmo-S1773	LG15	42.31	cgpGmo-S667	LG11	8.783
cgpGmo-S1784	LG15	42.752	cgpGmo-S1009	LG11	8.783
cgpGmo-S1920	LG15	45.044	cgpGmo-S681a	LG11	9.048
cgpGmo-S909	LG15	45.489	cgpGmo-S670	LG11	9.744
cgpGmo-S1707	LG15	45.823	cgpGmo-S1609a	LG11	10.378
cgpGmo-S238	LG15	45.976	cgpGmo-S403	LG11	14.032
cgpGmo-S296	LG15	46.908	cgpGmo-S1272	LG11	14.723
cgpGmo-S1201	LG15	48.499	cgpGmo-S967b	LG11	14.724
cgpGmo-S2142	LG15	49.232	cgpGmo-S967a	LG11	16.672
cgpGmo-S1755	LG15	49.541	cgpGmo-S1948	LG11	19.232
cgpGmo-S2178	LG15	50.802	cgpGmo-S1733	LG11	20.566
cgpGmo-S1077b	LG15	51.528	cgpGmo-S455	LG11	23.584
cgpGmo-S687	LG15	51.884	cgpGmo-S1484	LG11	27.515
cgpGmo-S1650	LG15	53.678	cgpGmo-S2232	LG11	30.826
cgpGmo-S726	LG15	55.546	cgpGmo-S1548	LG11	32.681
cgpGmo-S1082	LG15	60.371	cgpGmo-S1541a	LG11	33.134
cgpGmo-S46a	LG15	60.751	cgpGmo-S424	LG11	35.339
cgpGmo-S696	LG15	62.833	cgpGmo-S1802	LG11	36.023
cgpGmo-S1938	LG15	65.444	cgpGmo-S618	LG11	36.253
cgpGmo-S1035	LG15	66.349	cgpGmo-S1925	LG11	36.694
cgpGmo-S46b	LG15	72.226	cgpGmo-S1222	LG11	40.23
cgpGmo-S2166	LG16	0	cgpGmo-S939	LG11	41.522
cgpGmo-S972	LG16	0.011	cgpGmo-S1094	LG11	42.366
cgpGmo-S1350	LG16	1.218	cgpGmo-S154	LG11	42.366
cgpGmo-S464	LG16	5.297	cgpGmo-S613b	LG11	43.183
cgpGmo-S111b	LG16	5.435	cgpGmo-S691	LG11	43.775
cgpGmo-S111a	LG16	5.435	cgpGmo-S1063	LG11	43.934
cgpGmo-S2085	LG16	9.148	cgpGmo-S867	LG11	44.611
cgpGmo-S1032	LG16	9.29	cgpGmo-S1647	LG11	46.2

cgpGmo-S2224	LG16	9.659
cgpGmo-S109	LG16	12.028
cgpGmo-S944	LG16	29.104
cgpGmo-S113	LG16	29.676
cgpGmo-S497	LG16	30.083
cgpGmo-S1347	LG16	31.095
cgpGmo-S1664	LG16	31.219
cgpGmo-S2126	LG16	31.367
cgpGmo-S1281	LG16	31.882
cgpGmo-S842	LG16	31.882
cgpGmo-S533b	LG16	32.183
cgpGmo-S1941	LG16	32.992
cgpGmo-S1761	LG16	33.064
cgpGmo-S1188	LG16	36.463
cgpGmo-S213	LG16	40.063
cgpGmo-S1140	LG16	42.467
cgpGmo-S504	LG16	45.074
cgpGmo-S415	LG16	49.075
cgpGmo-S1073	LG16	49.599
cgpGmo-S287a	LG16	49.648
cgpGmo-S401	LG16	49.83
cgpGmo-S1042a	LG16	50.088
cgpGmo-S1290	LG16	50.499
cgpGmo-S508	LG16	50.995
cgpGmo-S2174	LG16	51.011
cgpGmo-S392	LG16	51.246
cgpGmo-S287b	LG16	51.536
cgpGmo-S2263	LG16	53.761
cgpGmo-S2138	LG16	55.436
cgpGmo-S1457	LG16	55.927
cgpGmo-S463b	LG16	57.787
cgpGmo-S1265b	LG17	0
cgpGmo-S561	LG17	2.55
cgpGmo-S1617	LG17	4.403
cgpGmo-S777	LG17	4.597
cgpGmo-S104	LG17	6.948
cgpGmo-S904	LG17	9.256
cgpGmo-S276b	LG17	12.399
cgpGmo-S2220	LG17	13.835
cgpGmo-S541b	LG17	14.625
cgpGmo-S727	LG17	15.249
cgpGmo-S541a	LG17	16.635
cgpGmo-S879	LG17	18.089
cgpGmo-S2184	LG17	18.11
cgpGmo-S2169	LG17	20.286
cgpGmo-S1974	LG17	20.954
cgpGmo-S566	LG17	21.241
cgpGmo-S811a	LG11	46.597
cgpGmo-S634	LG11	47.869
cgpGmo-S1658	LG11	47.923
cgpGmo-S4	LG11	48.222
cgpGmo-S1108	LG11	48.93
cgpGmo-S2179	LG11	51.114
cgpGmo-S150	LG11	51.357
cgpGmo-S2113	LG11	51.505
cgpGmo-S717	LG11	51.813
cgpGmo-S1998	LG11	52.439
cgpGmo-S79	LG11	53.521
cgpGmo-S1767	LG11	53.874
cgpGmo-S1431a	LG11	54.037
cgpGmo-S1712	LG11	54.09
cgpGmo-S2159	LG11	54.481
cgpGmo-S788	LG11	54.871
cgpGmo-S2211	LG11	55.251
cgpGmo-S607	LG11	55.341
cgpGmo-S2017	LG11	55.401
cgpGmo-S138	LG11	55.42
cgpGmo-S707	LG11	55.713
cgpGmo-S2102	LG11	55.913
cgpGmo-S1090	LG11	56.107
cgpGmo-S587	LG11	56.506
cgpGmo-S1843	LG11	56.56
cgpGmo-S386	LG11	56.563
cgpGmo-S2005	LG11	57.082
cgpGmo-S922	LG11	57.524
cgpGmo-S488	LG11	57.781
cgpGmo-S44	LG11	58.372
cgpGmo-S416b	LG11	60.138
cgpGmo-S581	LG11	60.939
cgpGmo-S416a	LG11	61.019
cgpGmo-S580	LG11	61.575
cgpGmo-S1384	LG11	67.558
cgpGmo-S594	LG12	-2.022
cgpGmo-S1846	LG12	-1.583
cgpGmo-S521b	LG12	-0.385
cgpGmo-S1956	LG12	0
cgpGmo-S1995	LG12	1.166
cgpGmo-S476	LG12	2.1
cgpGmo-S439	LG12	3.326
cgpGmo-S1225	LG12	3.842
cgpGmo-S1226	LG12	6.532
cgpGmo-S275	LG12	6.635
cgpGmo-S936	LG12	7.653
cgpGmo-S582	LG12	8.435

cgpGmo-S565	LG17	21.408	cgpGmo-S2209	LG12	12.952
cgpGmo-S1006	LG17	25.51	cgpGmo-S624	LG12	13.754
cgpGmo-S1041	LG17	27.255	cgpGmo-S251	LG12	14.796
cgpGmo-S878	LG17	28.613	cgpGmo-S248a	LG12	15.582
cgpGmo-S1780	LG17	36.764	cgpGmo-S57	LG12	16.59
cgpGmo-S955	LG17	40.254	cgpGmo-S866	LG12	16.674
cgpGmo-S616	LG17	44.423	cgpGmo-S1312	LG12	16.674
cgpGmo-S1864	LG17	44.765	cgpGmo-S1689	LG12	17.322
334C1CO1.411	LG17	44.765	cgpGmo-S1882	LG12	17.322
cgpGmo-S813	LG18	0	cgpGmo-S2032	LG12	17.327
cgpGmo-S2162	LG18	0.259	cgpGmo-S914	LG12	17.582
cgpGmo-S1323	LG18	2.067	cgpGmo-S596	LG12	17.647
cgpGmo-S2139	LG18	2.824	cgpGmo-S688	LG12	17.687
cgpGmo-S1294	LG18	6.382	cgpGmo-S1543	LG12	18.327
cgpGmo-S2175	LG18	10.523	cgpGmo-S180b	LG12	18.327
cgpGmo-S84	LG18	11.406	cgpGmo-S816a	LG12	18.327
cgpGmo-S706	LG18	12.252	cgpGmo-S372a	LG12	18.327
cgpGmo-S958	LG18	12.897	cgpGmo-S1260	LG12	18.327
cgpGmo-S1774	LG18	14.43	cgpGmo-S486	LG12	18.327
cgpGmo-S861	LG18	15.209	cgpGmo-S314	LG12	18.496
cgpGmo-S2259	LG18	17.901	cgpGmo-S116	LG12	18.552
cgpGmo-S2027	LG18	18.973	cgpGmo-S417	LG12	18.685
cgpGmo-S1300a	LG18	20.538	cgpGmo-S510	LG12	18.726
cgpGmo-S601	LG18	25.144	cgpGmo-S493	LG12	18.952
cgpGmo-S2077	LG18	26.418	cgpGmo-S1696	LG12	19.081
cgpGmo-S1441	LG18	28.49	cgpGmo-S229	LG12	19.149
cgpGmo-S331b	LG18	28.662	cgpGmo-S1737	LG12	19.69
cgpGmo-S1055a	LG18	30.204	cgpGmo-S636	LG12	19.814
cgpGmo-S1918	LG18	30.204	cgpGmo-S233	LG12	20.055
cgpGmo-S1435	LG18	30.362	cgpGmo-S2034	LG12	20.328
cgpGmo-S330	LG18	30.579	cgpGmo-S190	LG12	20.489
cgpGmo-S1115	LG18	31.046	cgpGmo-S1046	LG12	20.994
cgpGmo-S1117	LG18	31.371	cgpGmo-S502	LG12	21.31
cgpGmo-S1710	LG18	31.509	cgpGmo-S256	LG12	22.468
cgpGmo-S442a	LG18	31.528	cgpGmo-S1769	LG12	22.837
cgpGmo-S1095	LG18	31.997	cgpGmo-S1193	LG12	23.505
cgpGmo-S1379	LG18	33.299	cgpGmo-S316	LG12	24.168
cgpGmo-S391	LG18	34.204	cgpGmo-S2101	LG12	34.413
cgpGmo-S1340	LG18	36.968	cgpGmo-S742a	LG12	41.271
cgpGmo-S1992	LG18	37.609	cgpGmo-S348	LG13	0
cgpGmo-S916	LG18	38.116	cgpGmo-S2262	LG13	1.395
cgpGmo-S97	LG18	38.571	cgpGmo-S2177	LG13	3.791
cgpGmo-S1520	LG18	40.865	cgpGmo-S820	LG13	5.265
cgpGmo-S1818	LG18	40.87	cgpGmo-S294	LG13	6.199
cgpGmo-S900	LG18	42.993	cgpGmo-S1483	LG13	6.238
cgpGmo-S975b	LG18	44.927	cgpGmo-S1653	LG13	7.081
cgpGmo-S975a	LG18	46.983	cgpGmo-S1206	LG13	7.417

cgpGmo-S2187	LG19	0	cgpGmo-S2215	LG13	8.558
cgpGmo-S557	LG19	0.464	cgpGmo-S652	LG13	9.304
cgpGmo-S108	LG19	1.653	cgpGmo-S692a	LG13	10.072
cgpGmo-S1408	LG19	5.24	cgpGmo-S1695	LG13	10.427
cgpGmo-S1834	LG19	11.454	cgpGmo-S191	LG13	10.928
cgpGmo-S247	LG19	12.72	cgpGmo-S2067	LG13	11.398
cgpGmo-S1837	LG19	14.931	cgpGmo-S576	LG13	13.749
cgpGmo-S918	LG19	16.02	cgpGmo-S949a	LG13	15.449
cgpGmo-S1740	LG19	17.253	cgpGmo-S1097	LG13	21.73
cgpGmo-S649a	LG19	21.467	cgpGmo-S1069	LG13	25.235
cgpGmo-S495	LG19	22.939	cgpGmo-S980	LG13	25.235
cgpGmo-S649b	LG19	25.912	cgpGmo-S1889	LG13	26.776
cgpGmo-S621	LG19	28.232	cgpGmo-S281	LG13	29.942
cgpGmo-S1385b	LG19	29.336	cgpGmo-S2058	LG13	30.201
cgpGmo-S2120	LG19	29.648	cgpGmo-S1066	LG13	31.979
cgpGmo-S1385a	LG19	29.807	cgpGmo-S752b	LG13	32.271
cgpGmo-S1944	LG19	35.272	cgpGmo-S1961	LG13	33.206
cgpGmo-S1105	LG19	35.738	cgpGmo-S1390a	LG13	33.845
cgpGmo-S374	LG19	36.997	cgpGmo-S752a	LG13	35.405
cgpGmo-S329	LG19	38.111	cgpGmo-S399	LG13	37.081
cgpGmo-S328	LG19	38.111	cgpGmo-S1990	LG13	40.16
cgpGmo-S2143	LG19	39.444	cgpGmo-S29	LG13	42.535
cgpGmo-S1005	LG19	41.119	cgpGmo-S36a	LG13	43.083
cgpGmo-S633	LG19	41.652	cgpGmo-S36b	LG13	43.271
cgpGmo-S436	LG19	41.994	cgpGmo-S906	LG13	43.789
cgpGmo-S1528a	LG19	43.753	cgpGmo-S2018	LG13	43.931
cgpGmo-S1489	LG19	45.075	cgpGmo-S2013	LG13	44.233
cgpGmo-S642	LG19	45.075	cgpGmo-S350	LG13	46.343
cgpGmo-S443	LG19	45.58	cgpGmo-S1981	LG13	47.364
cgpGmo-S1775	LG19	48.338	cgpGmo-S487	LG13	47.976
cgpGmo-S366	LG19	50.46	cgpGmo-S1959	LG13	48.882
cgpGmo-S2130	LG19	54.762	cgpGmo-S765	LG13	48.967
cgpGmo-S767	LG19	55.24	cgpGmo-S1563	LG13	49.327
cgpGmo-S297	LG19	55.466	cgpGmo-S881	LG13	49.717
cgpGmo-S1461a	LG19	55.941	cgpGmo-S614b	LG13	49.882
cgpGmo-S1028	LG19	56.308	cgpGmo-S2281	LG13	50.029
cgpGmo-S193	LG19	56.366	cgpGmo-S1209	LG13	50.212
cgpGmo-S124	LG19	56.723	cgpGmo-S1762	LG13	50.247
cgpGmo-S1461b	LG19	56.907	cgpGmo-S905	LG13	50.638
cgpGmo-S1700	LG19	56.954	cgpGmo-S1639	LG13	50.638
cgpGmo-S1204	LG20	0	cgpGmo-S614a	LG13	50.926
cgpGmo-S2114	LG20	0	cgpGmo-S793a	LG13	51.512
cgpGmo-S2161	LG20	1.356	cgpGmo-S2160	LG13	51.52
cgpGmo-S995b	LG20	2.438	cgpGmo-S107	LG13	52.133
cgpGmo-S1297a	LG20	4.86	cgpGmo-S2028	LG13	52.232
cgpGmo-S1348	LG20	5.008	cgpGmo-S220	LG13	52.402
cgpGmo-S2269	LG20	5.291	cgpGmo-S2039	LG13	52.423

cgpGmo-S854b	LG20	5.565	cgpGmo-S241	LG13	54.058
cgpGmo-S501	LG20	6.95	cgpGmo-S1720	LG13	55.612
cgpGmo-S559	LG20	7.868	cgpGmo-S888	LG13	55.898
cgpGmo-S149	LG20	8.472	cgpGmo-S217a	LG13	59.672
cgpGmo-S995a	LG20	8.654	cgpGmo-S1977	LG13	60.54
cgpGmo-S1423a	LG20	10.209	cgpGmo-S1219c	LG14	0
cgpGmo-S1392	LG20	11.946	cgpGmo-S1219b	LG14	0.025
cgpGmo-S661a	LG20	12.961	cgpGmo-S1219a	LG14	0.025
cgpGmo-S661b	LG20	12.961	cgpGmo-S1665	LG14	1.25
cgpGmo-S1742	LG20	12.999	cgpGmo-S1530	LG14	3.15
cgpGmo-S1667	LG20	13.396	cgpGmo-S1725	LG14	7.134
cgpGmo-S1857	LG20	14.601	cgpGmo-S505	LG14	7.783
cgpGmo-S599	LG20	14.88	cgpGmo-S1760	LG14	8.372
cgpGmo-S693	LG20	15.708	cgpGmo-S1844	LG14	8.778
cgpGmo-S632a	LG20	15.834	cgpGmo-S252	LG14	9.333
cgpGmo-S632b	LG20	16.003	cgpGmo-S988	LG14	11.355
cgpGmo-S1391	LG20	17.914	cgpGmo-S2110	LG14	12.673
cgpGmo-S637	LG20	20.103	cgpGmo-S963	LG14	12.822
cgpGmo-S1184	LG20	29.981	cgpGmo-S631	LG14	13.242
cgpGmo-S1362	LG20	32.868	cgpGmo-S577	LG14	13.376
cgpGmo-S357	LG20	32.968	cgpGmo-S841	LG14	13.87
cgpGmo-S695	LG20	33.104	cgpGmo-S796	LG14	14.022
cgpGmo-S143	LG20	33.503	cgpGmo-S1922	LG14	14.09
cgpGmo-S635	LG20	35.073	cgpGmo-S1697	LG14	14.197
cgpGmo-S1503	LG20	35.746	cgpGmo-S462	LG14	19.181
cgpGmo-S196	LG20	37.395	cgpGmo-S427	LG14	19.551
cgpGmo-S431	LG20	38.348	cgpGmo-S617	LG14	21.131
cgpGmo-S525	LG20	40.291	cgpGmo-S1467	LG14	21.42
cgpGmo-S2201	LG20	40.904	cgpGmo-S1466b	LG14	21.627
cgpGmo-S1401	LG20	49.238	cgpGmo-S1466a	LG14	21.627
cgpGmo-S1482	LG20	51.022	cgpGmo-S1803	LG14	24.353
cgpGmo-S218	LG20	51.253	cgpGmo-S932b	LG14	24.805
cgpGmo-S2198	LG20	53.669	cgpGmo-S1914	LG14	25.094
cgpGmo-S1807	LG20	55.006	cgpGmo-S1049	LG14	29.476
cgpGmo-S1093	LG21	0	cgpGmo-S1792	LG14	30.507
cgpGmo-S772	LG21	1.182	cgpGmo-S1701	LG14	30.96
cgpGmo-S1794	LG21	1.773	cgpGmo-S1234	LG14	33.594
cgpGmo-S225a	LG21	2.899	cgpGmo-S302	LG14	33.872
cgpGmo-S2283	LG21	3.503	cgpGmo-S240	LG14	34.123
cgpGmo-S315	LG21	3.902	cgpGmo-S1821	LG14	34.921
cgpGmo-S130	LG21	6.188	cgpGmo-S1888	LG14	35.323
cgpGmo-S1084	LG21	6.36	cgpGmo-S1988	LG14	36.018
cgpGmo-S225b	LG21	6.412	cgpGmo-S520	LG14	36.056
cgpGmo-S1706	LG21	6.539	cgpGmo-S1424b	LG14	36.474
cgpGmo-S794	LG21	7.975	cgpGmo-S1968	LG14	36.573
cgpGmo-S1316	LG21	8.777	cgpGmo-S411	LG14	36.8
cgpGmo-S925	LG21	9.163	cgpGmo-S2078	LG14	36.922

cgpGmo-S2097	LG21	9.275	cgpGmo-S70	LG14	37.423
cgpGmo-S2171	LG21	10.157	cgpGmo-S827	LG14	37.903
cgpGmo-S858	LG21	11.338	cgpGmo-S1080	LG14	39.732
cgpGmo-S698	LG21	11.512	cgpGmo-S226	LG14	39.967
cgpGmo-S91	LG21	11.758	cgpGmo-S1394a	LG14	42.56
cgpGmo-S853	LG21	12.007	cgpGmo-S1280	LG14	43.692
cgpGmo-S1741	LG21	12.074	1057C1CO1.398	LG14	44.092
cgpGmo-S2055	LG21	16.8	cgpGmo-S1186	LG14	44.159
cgpGmo-S1465	LG21	18.477	cgpGmo-S965	LG14	44.317
cgpGmo-S1646	LG21	18.508	cgpGmo-S824	LG14	52.754
cgpGmo-S120	LG21	21.162	cgpGmo-S142	LG14	53.824
cgpGmo-S954	LG21	21.975	cgpGmo-S711b	LG14	54.095
cgpGmo-S1255b	LG21	22.301	cgpGmo-S503	LG14	55.34
cgpGmo-S1926	LG21	22.871	cgpGmo-S249	LG14	61.013
cgpGmo-S947	LG21	23.07	cgpGmo-S583	LG14	62.095
cgpGmo-S1255a	LG21	23.933	cgpGmo-S551	LG14	63.269
cgpGmo-S1972	LG21	24.42	cgpGmo-S1783	LG14	63.361
cgpGmo-S1342	LG21	24.48	cgpGmo-S92	LG15	0
cgpGmo-S224	LG21	25.197	cgpGmo-S1048	LG15	15.284
cgpGmo-S697	LG21	26.341	cgpGmo-S1752	LG15	20.943
cgpGmo-S907	LG21	27.278	cgpGmo-S1905	LG15	21.78
cgpGmo-S2063	LG21	33.074	cgpGmo-S2093	LG15	24.461
cgpGmo-S423	LG21	33.738	cgpGmo-S1770	LG15	24.461
cgpGmo-S459	LG21	42.039	cgpGmo-S677	LG15	27.108
cgpGmo-S808	LG21	42.33	cgpGmo-S676	LG15	28.475
cgpGmo-S1702	LG21	42.614	cgpGmo-S608	LG15	28.788
cgpGmo-S579	LG21	42.845	cgpGmo-S1621	LG15	29.322
cgpGmo-S549	LG21	43.231	cgpGmo-S298	LG15	29.717
cgpGmo-S1003	LG21	45.905	cgpGmo-S591	LG15	32.334
cgpGmo-S679	LG21	46.436	cgpGmo-S1781	LG15	33.806
cgpGmo-S2183	LG22	0	cgpGmo-S1728	LG15	35.152
cgpGmo-S740	LG22	0.995	cgpGmo-S1577	LG15	36.43
cgpGmo-S1552b	LG22	4.381	cgpGmo-S1899	LG15	37.159
cgpGmo-S1919	LG22	4.744	cgpGmo-S629	LG15	37.173
cgpGmo-S1552a	LG22	4.783	cgpGmo-S1896	LG15	38.025
cgpGmo-S1799	LG22	5.808	cgpGmo-S1773	LG15	39.18
cgpGmo-S1852	LG22	7.185	cgpGmo-S1784	LG15	39.771
cgpGmo-S1691	LG22	8.026	cgpGmo-S909	LG15	42.239
cgpGmo-S2121	LG22	8.206	cgpGmo-S238	LG15	42.397
cgpGmo-S20	LG22	8.751	cgpGmo-S1920	LG15	42.535
cgpGmo-S997b	LG22	12.263	cgpGmo-S1707	LG15	43.61
cgpGmo-S1417	LG22	14.065	cgpGmo-S296	LG15	45.183
cgpGmo-S206	LG22	14.931	cgpGmo-S1201	LG15	45.228
cgpGmo-S1909	LG22	16.008	cgpGmo-S2142	LG15	46.618
cgpGmo-S2125	LG22	17.693	cgpGmo-S2178	LG15	47.391
cgpGmo-S1106	LG22	18.802	cgpGmo-S687	LG15	48.153
cgpGmo-S2105	LG22	19.243	cgpGmo-S1755	LG15	48.215

cgpGmo-S1578	LG22	23.632	cgpGmo-S1077b	LG15	48.647
cgpGmo-S1904	LG22	23.632	cgpGmo-S1650	LG15	49.878
cgpGmo-S13b	LG22	23.964	cgpGmo-S726	LG15	51.839
cgpGmo-S1643	LG22	23.995	cgpGmo-S46a	LG15	58.665
155C1CO1.193	LG22	24.361	cgpGmo-S1082	LG15	60.22
cgpGmo-S996	LG22	24.371	cgpGmo-S696	LG15	62.392
cgpGmo-S2242	LG22	25.838	cgpGmo-S602	LG15	63.507
cgpGmo-S258	LG22	26.721	cgpGmo-S1057a	LG15	63.863
cgpGmo-S1659	LG22	27.897	cgpGmo-S1938	LG15	64.068
cgpGmo-S2288	LG22	31.856	cgpGmo-S1035	LG15	64.599
cgpGmo-S2186	LG22	33.199	cgpGmo-S542	LG15	65.308
cgpGmo-S263	LG22	34.918	cgpGmo-S1649	LG15	67.501
cgpGmo-S1308	LG22	34.918	cgpGmo-S46b	LG15	71.949
cgpGmo-S1382	LG22	36.663	cgpGmo-S972	LG16	0
cgpGmo-S1957	LG22	38.312	cgpGmo-S2166	LG16	0.303
cgpGmo-S822a	LG22	38.338	cgpGmo-S1350	LG16	2.678
cgpGmo-S1657	LG22	42.251	cgpGmo-S2085	LG16	4.777
cgpGmo-S962b	LG22	43.219	cgpGmo-S111b	LG16	6.3
cgpGmo-S1310	LG22	48.367	cgpGmo-S111a	LG16	6.3
cgpGmo-S28	LG22	61.815	cgpGmo-S464	LG16	6.329
cgpGmo-S805	LG22	61.815	cgpGmo-S2224	LG16	10.638
cgpGmo-S1804	LG22	68.501	cgpGmo-S109	LG16	11.333
cgpGmo-S418	LG23	0	cgpGmo-S1339	LG16	11.622
cgpGmo-S1596a	LG23	0.953	cgpGmo-S1032	LG16	13.163
cgpGmo-S1071a	LG23	3.71	cgpGmo-S195	LG16	18.386
5911C1CO1.447	LG23	5.456	cgpGmo-S2287	LG16	22.321
cgpGmo-S1622	LG23	5.767	cgpGmo-S1797	LG16	22.831
cgpGmo-S626b	LG23	5.818	cgpGmo-S497	LG16	23.681
cgpGmo-S626a	LG23	6.172	cgpGmo-S1761	LG16	26.779
cgpGmo-S227	LG23	9.797	cgpGmo-S1941	LG16	27.456
cgpGmo-S1202	LG23	19.065	cgpGmo-S1608	LG16	28.367
cgpGmo-S1250	LG23	20.518	cgpGmo-S113	LG16	30.156
cgpGmo-S623	LG23	20.627	cgpGmo-S2126	LG16	32.081
cgpGmo-S838a	LG23	20.814	cgpGmo-S533b	LG16	32.095
cgpGmo-S528	LG23	21.88	cgpGmo-S1664	LG16	32.309
cgpGmo-S458a	LG23	22.176	cgpGmo-S1347	LG16	32.418
cgpGmo-S849	LG23	22.572	cgpGmo-S1281	LG16	32.488
cgpGmo-S351	LG23	23.115	cgpGmo-S842	LG16	32.488
cgpGmo-S606b	LG23	23.482	cgpGmo-S1188	LG16	32.637
cgpGmo-S1903	LG23	24.195	cgpGmo-S944	LG16	33.803
cgpGmo-S606a	LG23	24.195	cgpGmo-S2106	LG16	34.909
cgpGmo-S722	LG23	24.195	cgpGmo-S1243b	LG16	36.685
cgpGmo-S1506	LG23	24.237	cgpGmo-S600	LG16	36.685
cgpGmo-S1475	LG23	25.199	cgpGmo-S1243a	LG16	36.685
2311C1CO1.535	LG23	27.3	cgpGmo-S213	LG16	39.835
cgpGmo-S994	LG23	28.607	cgpGmo-S1947	LG16	42.392
cgpGmo-S272	LG23	31.918	cgpGmo-S1140	LG16	42.479

cgpGmo-S2035	LG23	32.129	cgpGmo-S392	LG16	43.582
			cgpGmo-S2174	LG16	44.961
			cgpGmo-S508	LG16	44.962
			cgpGmo-S504	LG16	44.964
			cgpGmo-S515	LG16	45.708
			cgpGmo-S1042a	LG16	47.38
			cgpGmo-S1290	LG16	48.108
			cgpGmo-S401	LG16	48.548
			cgpGmo-S1042b	LG16	48.667
			cgpGmo-S415	LG16	48.715
			cgpGmo-S287a	LG16	49.034
			cgpGmo-S1073	LG16	49.051
			cgpGmo-S627	LG16	49.394
			cgpGmo-S2164	LG16	50.165
			cgpGmo-S287b	LG16	50.901
			cgpGmo-S2263	LG16	51.414
			cgpGmo-S2138	LG16	53.067
			cgpGmo-S1457	LG16	53.208
			cgpGmo-S463b	LG16	54.433
			cgpGmo-S300	LG16	56.582
			cgpGmo-S437	LG16	64.659
			cgpGmo-S1265b	LG17	0
			cgpGmo-S285	LG17	0.679
			cgpGmo-S1172	LG17	0.763
			cgpGmo-S561	LG17	2.367
			cgpGmo-S1617	LG17	4.043
			cgpGmo-S777	LG17	4.283
			cgpGmo-S1265c	LG17	5.994
			cgpGmo-S104	LG17	6.449
			cgpGmo-S904	LG17	8.757
			cgpGmo-S276b	LG17	11.683
			cgpGmo-S2220	LG17	13.332
			cgpGmo-S541b	LG17	13.748
			cgpGmo-S727	LG17	14.716
			cgpGmo-S541a	LG17	16.128
			cgpGmo-S2184	LG17	17.961
			cgpGmo-S879	LG17	18.351
			cgpGmo-S2169	LG17	19.589
			cgpGmo-S565	LG17	20.918
			cgpGmo-S566	LG17	20.946
			cgpGmo-S1974	LG17	20.959
			cgpGmo-S1006	LG17	24.62
			cgpGmo-S1041	LG17	27.17
			cgpGmo-S878	LG17	28.67
			cgpGmo-S1780	LG17	37.092
			cgpGmo-S2212	LG17	37.404
			cgpGmo-S1056	LG17	37.776

cgpGmo-S1655	LG17	38.353
cgpGmo-S1738	LG17	39.003
cgpGmo-S955	LG17	39.045
cgpGmo-S381	LG17	44.027
cgpGmo-S1864	LG17	47.016
334C1CO1.411	LG17	47.016
cgpGmo-S616	LG17	48.179
cgpGmo-S650b	LG17	56.591
cgpGmo-S2041	LG18	0
cgpGmo-S9b	LG18	0.881
cgpGmo-S2162	LG18	1.049
cgpGmo-S813	LG18	1.509
cgpGmo-S1474	LG18	2.207
cgpGmo-S1323	LG18	2.229
cgpGmo-S2139	LG18	3.652
cgpGmo-S1294	LG18	5.806
cgpGmo-S2175	LG18	11.38
cgpGmo-S84	LG18	13.158
cgpGmo-S958	LG18	13.914
cgpGmo-S706	LG18	14.307
cgpGmo-S1774	LG18	15.954
cgpGmo-S861	LG18	16.783
cgpGmo-S2259	LG18	19.303
cgpGmo-S2027	LG18	20.189
cgpGmo-S1300a	LG18	21.775
cgpGmo-S197a	LG18	25.359
cgpGmo-S601	LG18	26.353
cgpGmo-S2077	LG18	27.651
cgpGmo-S1441	LG18	29.279
cgpGmo-S331b	LG18	29.663
cgpGmo-S1918	LG18	31.186
cgpGmo-S1055a	LG18	31.186
cgpGmo-S1435	LG18	31.269
cgpGmo-S330	LG18	31.782
cgpGmo-S1115	LG18	32.185
cgpGmo-S1117	LG18	32.342
cgpGmo-S1710	LG18	32.463
cgpGmo-S442a	LG18	32.466
cgpGmo-S1095	LG18	33.187
cgpGmo-S1379	LG18	34.774
cgpGmo-S391	LG18	35.792
cgpGmo-S1340	LG18	38.173
cgpGmo-S1992	LG18	39.198
cgpGmo-S916	LG18	39.561
cgpGmo-S97	LG18	40.023
cgpGmo-S684	LG18	40.121
cgpGmo-S1818	LG18	42.225

cgpGmo-S1520	LG18	42.229
cgpGmo-S900	LG18	44.668
cgpGmo-S975b	LG18	46.327
cgpGmo-S975a	LG18	48.484
cgpGmo-S1083	LG18	49.226
cgpGmo-S2187	LG19	0
cgpGmo-S557	LG19	0.053
cgpGmo-S108	LG19	1.707
cgpGmo-S1408	LG19	5.157
cgpGmo-S1834	LG19	11.603
cgpGmo-S247	LG19	13.05
cgpGmo-S1837	LG19	15.633
cgpGmo-S918	LG19	16.422
cgpGmo-S1740	LG19	17.604
cgpGmo-S649a	LG19	21.388
cgpGmo-S665	LG19	23.427
cgpGmo-S495	LG19	24.147
cgpGmo-S649b	LG19	26.271
cgpGmo-S621	LG19	28.113
cgpGmo-S2120	LG19	29.31
cgpGmo-S1385a	LG19	29.483
cgpGmo-S1385b	LG19	29.793
cgpGmo-S586	LG19	34.172
cgpGmo-S1944	LG19	35.093
cgpGmo-S1105	LG19	35.205
cgpGmo-S374	LG19	37.04
cgpGmo-S328	LG19	38.165
cgpGmo-S329	LG19	38.165
cgpGmo-S911	LG19	39.153
cgpGmo-S2143	LG19	39.59
cgpGmo-S633	LG19	41.345
cgpGmo-S1005	LG19	41.366
cgpGmo-S436	LG19	41.98
cgpGmo-S1528a	LG19	43.698
cgpGmo-S642	LG19	44.72
cgpGmo-S1489	LG19	44.72
cgpGmo-S443	LG19	45.652
cgpGmo-S1775	LG19	48.839
cgpGmo-S366	LG19	50.409
cgpGmo-S271	LG19	50.773
cgpGmo-S767	LG19	54.661
cgpGmo-S2130	LG19	54.96
cgpGmo-S297	LG19	55.085
cgpGmo-S1461a	LG19	56.108
cgpGmo-S1014b	LG19	56.211
cgpGmo-S193	LG19	56.329
cgpGmo-S1700	LG19	56.378

cgpGmo-S1028	LG19	56.652
cgpGmo-S124	LG19	56.723
cgpGmo-S1461b	LG19	57.424
cgpGmo-S2161	LG20	0
cgpGmo-S995b	LG20	1.042
cgpGmo-S1204	LG20	2.276
cgpGmo-S2114	LG20	2.276
cgpGmo-S1297a	LG20	5.974
cgpGmo-S1348	LG20	6.158
cgpGmo-S2269	LG20	6.558
cgpGmo-S854b	LG20	6.651
cgpGmo-S501	LG20	8.103
cgpGmo-S559	LG20	9.033
cgpGmo-S149	LG20	9.695
cgpGmo-S995a	LG20	9.828
cgpGmo-S1423a	LG20	11.374
cgpGmo-S1392	LG20	13.089
cgpGmo-S661b	LG20	14.073
cgpGmo-S661a	LG20	14.073
cgpGmo-S1742	LG20	14.129
cgpGmo-S1667	LG20	14.612
cgpGmo-S1857	LG20	15.885
cgpGmo-S599	LG20	16.065
cgpGmo-S632a	LG20	16.995
cgpGmo-S632b	LG20	17.106
cgpGmo-S1945	LG20	17.225
cgpGmo-S693	LG20	17.294
cgpGmo-S1391	LG20	19.171
cgpGmo-S637	LG20	21.642
cgpGmo-S1454	LG20	27.825
cgpGmo-S1184	LG20	32.041
cgpGmo-S1362	LG20	33.811
cgpGmo-S357	LG20	33.836
cgpGmo-S695	LG20	34.339
cgpGmo-S143	LG20	34.593
cgpGmo-S635	LG20	36.087
cgpGmo-S1503	LG20	36.822
cgpGmo-S196	LG20	38.313
cgpGmo-S431	LG20	39.485
cgpGmo-S525	LG20	41.375
cgpGmo-S2201	LG20	41.731
cgpGmo-S1401	LG20	50.075
cgpGmo-S1482	LG20	52.047
cgpGmo-S218	LG20	52.241
cgpGmo-S2198	LG20	54.71
cgpGmo-S1807	LG20	56.06
cgpGmo-S1093	LG21	0

cgpGmo-S1573	LG21	0.934
cgpGmo-S772	LG21	1.23
cgpGmo-S1794	LG21	1.779
cgpGmo-S225a	LG21	2.934
cgpGmo-S2283	LG21	3.655
cgpGmo-S315	LG21	3.993
cgpGmo-S1706	LG21	6.054
cgpGmo-S130	LG21	6.247
cgpGmo-S225b	LG21	6.392
cgpGmo-S1084	LG21	6.417
cgpGmo-S794	LG21	7.995
cgpGmo-S1316	LG21	8.771
cgpGmo-S925	LG21	9.125
cgpGmo-S2097	LG21	9.261
cgpGmo-S2171	LG21	10.156
cgpGmo-S858	LG21	11.297
cgpGmo-S698	LG21	11.433
cgpGmo-S1741	LG21	12.138
cgpGmo-S91	LG21	12.756
cgpGmo-S853	LG21	14.259
cgpGmo-S2055	LG21	16.574
cgpGmo-S1465	LG21	18.629
cgpGmo-S1646	LG21	19.653
cgpGmo-S954	LG21	20.57
cgpGmo-S120	LG21	20.958
cgpGmo-S1255b	LG21	22.052
cgpGmo-S1926	LG21	22.525
cgpGmo-S947	LG21	22.69
cgpGmo-S1255a	LG21	22.967
cgpGmo-S1972	LG21	24.019
cgpGmo-S224	LG21	24.079
cgpGmo-S1342	LG21	24.219
cgpGmo-S697	LG21	25.675
cgpGmo-S907	LG21	26.917
cgpGmo-S675a	LG21	28.335
cgpGmo-S2063	LG21	31.746
cgpGmo-S423	LG21	32.505
cgpGmo-S459	LG21	41.011
cgpGmo-S808	LG21	41.279
cgpGmo-S579	LG21	41.493
cgpGmo-S1702	LG21	41.539
cgpGmo-S549	LG21	42.157
cgpGmo-S1003	LG21	44.071
cgpGmo-S679	LG21	45.185
cgpGmo-S2183	LG22	0
cgpGmo-S740	LG22	0.894
cgpGmo-S1705	LG22	2.974

cgpGmo-S1552b	LG22	4.344
cgpGmo-S1552a	LG22	4.379
cgpGmo-S1799	LG22	4.807
cgpGmo-S1919	LG22	4.866
cgpGmo-S1852	LG22	7.229
cgpGmo-S20	LG22	7.87
cgpGmo-S2121	LG22	8.356
cgpGmo-S1691	LG22	9.014
cgpGmo-S1417	LG22	13.602
cgpGmo-S997b	LG22	13.917
cgpGmo-S206	LG22	15.369
cgpGmo-S1909	LG22	15.853
cgpGmo-S382	LG22	17.351
cgpGmo-S2125	LG22	17.566
cgpGmo-S1335	LG22	18.541
cgpGmo-S2105	LG22	18.672
cgpGmo-S1106	LG22	18.966
cgpGmo-S1643	LG22	22.843
cgpGmo-S13b	LG22	23.068
cgpGmo-S996	LG22	23.939
155C1CO1.193	LG22	24.01
cgpGmo-S2242	LG22	25.091
cgpGmo-S1904	LG22	25.305
cgpGmo-S1578	LG22	25.305
cgpGmo-S258	LG22	26.054
cgpGmo-S1659	LG22	27.523
cgpGmo-S2288	LG22	33.444
cgpGmo-S2186	LG22	34.653
cgpGmo-S2108	LG22	34.909
cgpGmo-S822a	LG22	36.419
cgpGmo-S1382	LG22	36.896
cgpGmo-S1308	LG22	37.333
cgpGmo-S263	LG22	37.333
cgpGmo-S1957	LG22	38.172
5681C1CO1.227	LG22	39.997
cgpGmo-S1657	LG22	40.184
cgpGmo-S1718	LG22	40.477
cgpGmo-S962b	LG22	41.857
cgpGmo-S1310	LG22	46.161
cgpGmo-S28	LG22	59.459
cgpGmo-S805	LG22	59.459
cgpGmo-S1804	LG22	69.959
cgpGmo-S1008	LG23	0
cgpGmo-S418	LG23	0.802
cgpGmo-S1596a	LG23	1.273
cgpGmo-S1766	LG23	2.647
cgpGmo-S1071a	LG23	4.577

cgpGmo-S335	LG23	4.779
cgpGmo-S1622	LG23	5.906
cgpGmo-S227	LG23	6.682
cgpGmo-S626a	LG23	6.752
cgpGmo-S626b	LG23	7.065
5911C1CO1.447	LG23	7.758
cgpGmo-S2218	LG23	16.686
cgpGmo-S529	LG23	18.251
cgpGmo-S897	LG23	18.251
cgpGmo-S1202	LG23	19.399
cgpGmo-S623	LG23	20.777
cgpGmo-S838a	LG23	22.042
cgpGmo-S528	LG23	22.84
cgpGmo-S458a	LG23	22.988
cgpGmo-S849	LG23	23.314
cgpGmo-S1250	LG23	23.479
cgpGmo-S351	LG23	23.983
cgpGmo-S1903	LG23	25.086
cgpGmo-S606a	LG23	25.086
cgpGmo-S722	LG23	25.086
cgpGmo-S1506	LG23	25.405
cgpGmo-S606b	LG23	25.423
cgpGmo-S1475	LG23	26.35
cgpGmo-S2086	LG23	27.898
2311C1CO1.535	LG23	28.516
cgpGmo-S272	LG23	31.582
cgpGmo-S994	LG23	31.644
cgpGmo-S2035	LG23	32.016
cgpGmo-S1320	LG23	39.83
